# Performance of Multiplex Commercial Kits to Quantify Cytokine and Chemokine Responses in Culture Supernatants from *Plasmodium falciparum* Stimulations

**DOI:** 10.1371/journal.pone.0052587

**Published:** 2013-01-02

**Authors:** Gemma Moncunill, John J. Aponte, Augusto J. Nhabomba, Carlota Dobaño

**Affiliations:** 1 Barcelona Centre for International Health Research (CRESIB, Hospital Clínic-Universitat de Barcelona), Barcelona, Catalonia, Spain; 2 Centro de Investigação em Saúde de Manhiça, Maputo, Mozambique; London School of Hygiene and Tropical Medicine, United Kingdom

## Abstract

**Background:**

Cytokines and chemokines are relevant biomarkers of pathology and immunity to infectious diseases such as malaria. Several commercially available kits based on quantitative suspension array technologies allow the profiling of multiple cytokines and chemokines in small volumes of sample. However, kits are being continuously improved and information on their performance is lacking.

**Methodology/Principal Findings:**

Different cytokine/chemokine kits, two flow cytometry-based (eBioscience® FlowCytomix™ and BD™ Cytometric Bead Array Human Enhanced Sensitivity) and four Luminex®-based (Invitrogen™ Human Cytokine 25-Plex Panel, Invitrogen™ Human Cytokine Magnetic 30-Plex Panel, Bio-Rad® Bio-Plex Pro™ Human Cytokine Plex Assay and Millipore™ MILLIPLEX® MAP Plex Kit) were compared. Samples tested were supernatants of peripheral blood mononuclear cells of malaria-exposed children stimulated with *Plasmodium falciparum* parasite lysates. Number of responses in range that could be detected was determined and reproducibility of duplicates was evaluated by the Bland-Altman test. Luminex® kits performed better than flow cytometry kits in number of responses in range and reproducibility. Luminex® kits were more reproducible when magnetic beads were used. However, within each methodology overall performance depended on the analyte tested in each kit. Within the Luminex® kits, the Invitrogen™ with polystyrene beads had the poorer performance, whereas Invitrogen™ with magnetic beads had the higher percentage of cytokines/chemokines with both readings in range (40%), followed by Bio-Rad® with magnetic beads (35%). Regarding reproducibility, the Millipore™ kit had the highest percentage (60%) of cytokines/chemokines with acceptable limits of agreement (<30%), followed by the Invitrogen™ with magnetic beads (40%) that had tighter limits of agreement.

**Conclusions/Significance:**

Currently available kits for cytokine and chemokine quantification differ in reproducibility and concentration range of accurate detection. Luminex®-based kits with magnetic beads perform the best. Data highlights the importance of testing different kits before each study to choose the most appropriate, depending on the priority of the cytokines assessed.

## Introduction

Complex infectious diseases such as malaria induce intricate immune responses that may result in pathogenesis or protection. It is unlikely that single immune mediators on their own correlate with disease risk or protective immunity. Instead, multiple cytokines and chemokines are involved in pathological and immunological processes [1], reflecting the different capacities and functions of immune cells such as immunoregulation, proliferation, activation or cytotoxicity. Immune assays such as ELIspot, intracellular cytokine staining and flow cytometry or ELISA are limited in the number of variables able to measure at the same time in a single assay, whereas quantitative suspension array technology allows the simultaneous measurement of different cytokines/chemokines. This technology is based on a capture-detection sandwich type assay using fluorescent microspheres, and only requires small volumes of samples. The possibility of measuring multiple variables is of importance for malaria and other major infectious diseases in which no immune correlates of protection have been identified [2], [3]. Quantitative suspension array technology allows a more in depth characterization of antigen specific responses, increasing the chances to detect cytokine/chemokine responses associated with protective immunity.

The introduction of fluorescent bead-based technology represents a significant advancement for cytokine/chemokine profiling. The assessment of multiple analytes permits to discover patterns and relevant correlations and may be more powerful to capture the emergence of complex responses associated to protection or pathology. The requirement of a reduced sample volume and time saving-advantages make it an attractive method to study immune responses and biomarkers in large-scale pediatric field studies. Multiplexing technologies have proved crucial in deciphering patterns and provide insight to pathology and immunity in a number of studies [4]–[9]. However, there are different methods (Luminex®-based or flow cytometry-based) and several available commercial kits that vary in absolute cytokine concentration, sensitivity, reproducibility and cost [10]–[16]. Khan *et al.* compared four different Luminex®-based kits [14] and found that despite non-comparable absolute concentrations, cytokines may follow similar patterns between different kits. However, only five analytes were compared in this study. It has been recommended to use the same method if comparisons are to be made between different data sets and to validate them for reproducibility and precision to ensure accurate protein identification [17].

To date, most of the studies comparing different multiplex kits have measured cytokines in serum or plasma samples [10]–[12], [14], [16]. In this study, we evaluate cytokine/chemokine concentrations in supernatants of stimulated peripheral blood mononuclear cells (PBMC). We previously compared two Luminex® methods, one from Bio-Rad® and another from Invitrogen™, and two flow cytometry methods, one from Becton Dickinson™ (BD™) and one from Bender MedSystems® that were commercially available in 2007 [15]. However, this technology is continuously evolving and to our knowledge data of newest available commercial kits are missing in the literature, particularly regarding the performance of magnetic beads. Therefore, the aim of this study is to compare the performance of six human cytokine/chemokine multiplex kits currently available using culture supernatants from PBMC stimulated with malaria parasite lysates. The kits from the following manufacturers were tested: eBiosciences® (formerly Bender MedSystems®), Bio-Rad®, Millipore™, Invitrogen™ and BD®.

## Methods

### Ethics Statement

The study has been approved by the ethics committees of the Hospital Clinic of Barcelona (Spain) and the National Health Bioethics Committee of Mozambique. Written informed consent was obtained from parents or guardians.

### Samples

Culture supernatants had been harvested after fresh stimulation of 200,000 PBMC with a *P. falciparum* schizont lysate (3D7 strain), corresponding to 0.6 million infected erythrocytes, for 48 h and 72 h, as described [15]. Briefly, PBMC obtained from children were isolated using a Lymphoprep gradient and resuspended in RPMI 1640 medium with 10% FCS, 100 IU/ml penicillin, 0.1 mg/ml streptomycin (all Sigma®), and 2 mM L-glutamine (GIBCO/Invitrogen™). Blood samples had been collected into EDTA microtainers by finger-prick from children <2 years old living in a malaria endemic area in the context of a study conducted at the Centro de Investigaçao em Saúde da Manhiça, southern Mozambique, between 2005 and 2009 (ClinicalTrials.gov NCT00231452) [18]. Following the incubation period, the supernatants were stored at -80°C in Manhiça and shipped in dry ice to Barcelona for analysis. Before the tests, supernatants were thawed, aliquoted and frozen again.

### Cytokine and Chemokine Measurement by Multiplex Bead Array Kits

Initially we evaluated four different kits: (i) the Human Th1/Th2 11-Plex FlowCytomix™ kit plus 3 extra cytokines from eBioscience® (formerly Bender MedSystems®, flow cytometry, non-magnetic beads), (ii) the Human Cytokine 25-Plex panel from Invitrogen™ (Luminex®, non-magnetic beads), (iii) the Bio-Plex Pro™ Human Cytokine 17-Plex Assay from Bio-Rad® (Luminex®, magnetic beads) and (iv) the MILLIPLEX® MAP 13-Plex Kit from Millipore™ (Luminex®, magnetic beads). During the testing, Invitrogen™ made available a new improved multiplex kit with magnetic beads, the Human cytokine Magnetic 30-Plex Panel (Luminex®) that was included in a second round of tests together with a BD™ Cytometric Bead Array (CBA) Human Enhanced Sensitivity 3-Plex kit (flow cytometry, non-magnetic beads). Therefore, two rounds of tests were performed in this study. In the first round, 37 samples from 48 h stimulations were analyzed in duplicates, whereas in the second round 40 samples from 72 h stimulations were analyzed also in duplicates. The second round included all 37 samples from the first round, but at a different stimulation time, plus 3 additional new samples. Nevertheless, only 20 of these samples could be analyzed with the BD™ CBA kit. In addition, a negative control of only media was added in each plate. The cytokines/chemokines tested in each kit are detailed in Table 1 and Table 2. The Bio-Plex assay kit from Bio-Rad®, the Invitrogen™ Human cytokine Magnetic 30-plex panel, and the BD™ CBA assays were carried out in the presence of a representative from the manufacturer. All methods were performed by the same operator according to manufacturers’ instructions. All kits supplied lyophilized standards that were reconstituted and diluted at 7 serial concentrations following manufacturer’s instructions (standard curves). Standards included all recombinant cytokines tested and were considered as positive controls for the procedure. However, for the Bio-Rad®, Invitrogen™ and Millipore™ kits that recommend 50 µl, samples were diluted 1∶2 in culture media, to be able to compare the performance of the kits using minimum equivalent volumes (25 µl). Bead fluorescence readings were done by Luminex® (Bio-Plex® 100) or by a flow cytometry (BD™ FACSCanto II) apparatus.

**Table 1:** Kits characteristics.

Characteristics	CBA Human Enhanced Sensitivity	FlowCytomix™ Multiplex	Bio-Plex Pro™ Assays	MILLIPLEX® MAP Plex	Invitrogen™25-Plex	Invitrogen™ Magnetic 30-Plex
Vendor	BD™	eBioscience®	Bio-Rad®	Millipore™	Invitrogen™	Invitrogen™
Reading method	Flow cytometry	Flow cytometry	Luminex®	Luminex®	Luminex®	Luminex®
Magnetic beads	No	No	Yes	Yes	No	yes
Cost^a^	1	3	2	1	4	4
Incubation time	5 h	3 h 30 min	1 h 10 min	3 h 30 min	3 h 30 min	3 h 30 min
Plate reading time	2 h 30 min	2 h 30 min	30 min	30 min	1 h	30 min
Amount sample	50 µl	25 µl	50 µl	25 µl	50 µl	50 µl
Amount tested sample	25 µl	25 µl	25 µl	25 µl	25 µl	25 µl
N° of tested cytokines	3	12	17	13	25	30

a Cost: 1 = highest cost; 4 = lowest cost.

**Table 2:** Proportion of duplicate readings in range.

	Round 1				Round 2		
Analyte	FlowCytomix™	Bio-Rad®	Invitrogen™	Millipore™	INV-MAG	BD™ CBA	Best perfomance
EGF	.	.	.	.	**95**	.	INV-MAG
EOTAXIN	.	.	0	.	0	.	–
FGF-Basic	.	.	.	.	47.5	.	INV-MAG
G-CSF	.	**86.5**	.	.	65	.	Bio-Rad®
GM-CSF	.	**97.3**	8.1	.	10	.	Bio-Rad®
HGF	.	.	.	.	**95.5**	.	INV-MAG
IFN-α	.	.	8.1	.	**87.5**	.	INV-MAG
IFN-γ	18.9	70.3	10.8	24.3	5	0	Bio-Rad®
IL-10	21.6	**86.5**	10.8	62.5	62.5	.	Bio-Rad®
IL-12	0	64.9	54.1	13.5	**97.5**	.	INV-MAG
IL-13	5.4	67.6	2.7	.	**80**	.	INV-MAG
IL-15	.	.	0	.	55	.	INV-MAG
IL-17	0	59.5	2.7	2.7	27.5	.	Bio-Rad®
IL-1β	59.5	**81.1**	45.9	**78.4**	67.5	.	Bio-Rad®
IL-1Ra	.	-	56.8	-	**97.5**	.	INV-MAG
IL-2	0	45.9	0	5.4	47.5	35	INV-MAG
IL-22	0	.	.	.	.	.	–
IL-2R	.	.	37.8	.	**75**	.	INV-MAG
IL-4	0	**89.2**	0	48.6	10	15	Bio-Rad®
IL-5	0	32.4	0	5.4	2.7	.	Bio-Rad®
IL-6	**78.4**	56.8	56.8	67.6	**75**	.	FlowCytomix™
IL-7	.	**97.3**	0	0	17.5	.	Bio-Rad®
IL-8	**81.1**	10.8	40.5	35.1	22.5	.	FlowCytomix™
IP-10	.	.	18.9	.	**100**	.	INV-MAG
MCP-1	.	40.5	56.8	.	60	.	INV-MAG
MIG	.	.	0	.	47.5	.	INV-MAG
MIP-1α	.	.	56.8	.	**97.5**	.	INV-MAG
MIP-1β	.	54.1	56.8	.	67.5	.	INV-MAG
RANTES	.	-	**89.2**	.	**100**	.	INV-MAG
TNF	43.2	54.1	13.5	**75.7**	60	.	Millipore™
TNF-β	0	.	.	10.8	-	.	Millipore™
VEGF	.	.	.	.	**97.5**	.	INV-MAG
N° Analytes >75%/total analytes (%)	2/14 (14.3%)	6/17 (35.3%)	1/25 (4.0%)	2/13 (15.4%)	12/30 (40.0%)	0/3 (0.0%)	INV-MAG

. (not tested).

Proportions of both readings in range higher than 75% are highlighted in bold.

INV-MAG: Invitrogen™ kit with magnetic beads.

### Statistical Analysis

Data for each kit was analyzed as recommended by the manufacturers. Concentration of each analyte was obtained by interpolating fluorescence intensity to at least 7-point dilution standard curve supplied by the kit and calculated by the Bio-Plex® software (Bio-Plex® Manager version 4.0), by the FlowCytomix™ Pro 2.2.1 software (eBiosciences®) or the BD FCAP Array™ Software, depending on the kit. The proportion of samples that had both readings within the accepted recovery range (between 70–130%) was determined. The reproducibility of the different multiplexed methods was evaluated by describing the limits of agreement between duplicates in range for each combination of method and analyte using the Bland-Altman test. Mean difference dot plots with the limits of agreement were constructed [19]. Comparison of variability and bias between the two readings was evaluated using the MethComp package [20] in R statistical software (version 2.12.2) [21]. P-values lower than 0.05 were considered significant.

## Results and Discussion

In this study we compared six different kits (Table 1) using supernatants of antigen-stimulated PBMC from children to measure cytokine and chemokine responses specific to *P. falciparum* malaria. Quantitative suspension array technology allowing the multiplex detection of up to 39 cytokines (depending on kit) is a unique tool to study immune responses in small sample volumes. It is particularly relevant in malaria research since no immune correlates of protection have yet been identified. The quantitative microsphere-based suspension array system provides a more comprehensive representation of the immune responses than assays assessing individual biomarkers and it is useful to address the complexity of immunity to infectious diseases.

**Figure 1:**
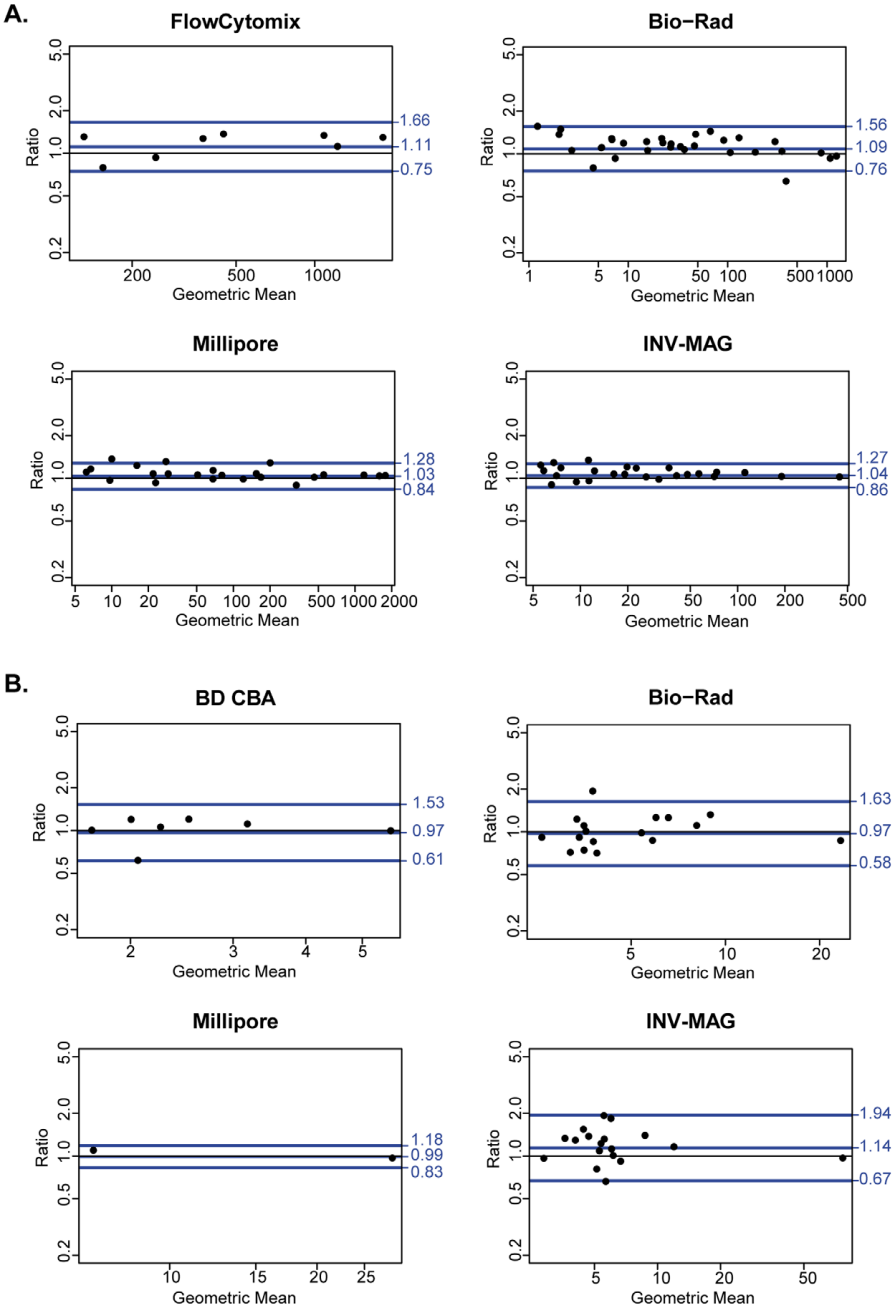
Mean difference dot plots of IL-10 and IL-2 cytokines for each kit tested. A) IL-10 and B) IL-2 disagreement plots show the difference between the duplicates against the geometric mean of both values of a sample tested with eBioscience® FlowCytomix™ (FlowCytomix), Millipore™ MILLIPLEX® MAP Plex Kit (Millipore), Bio-Rad® Bio-Plex Pro™ Human Cytokine Plex Assay (Bio-Rad), Invitrogen™ Human Cytokine Magnetic 30-Plex Panel (INV-MAG) and BD™ Cytometric Bead Array Human Enhanced Sensitivity kit (BD CBA). The middle line is the mean difference and the two extreme lines are the limits of agreement (values are truncated to the first decimal) calculated by Bland-Altman test.

We evaluated the performance of each kit by calculating the number of samples that had both readings in the accurate range of detection (Table 2) and the reproducibility (Table 3) of each analyte tested for each kit. Differences of less than 30% in the limits of agreement were considered acceptable. Tables 1 and 2 show the number of analytes tested as well as the specific cytokines/chemokines analyzed, respectively. There are no gold standards available to evaluate the accuracy and precision of the absolute concentrations measured by the kits. In each kit the cytokines were chosen by us based on the analytes available, the total number of analytes included by the manufacturers and the cytokine priorities based on the malaria scientific literature. As an example of the Bland-Altman plots generated for each cytokine in each tested kit, the graphs for IL-10 and IL-2 are represented in Figure 1, whereas the rest are shown in Supporting Information (Figure S1, FS2, FS3, FS4, FS5, FS6, FS7, FS8, FS9, FS10, FS11, FS12, FS13, FS14, FS15, FS16, FS17, FS18, FS19, FS20, FS21, FS22, FS23, FS24, FS25, FS26, FS27, FS28, FS29, S30).

**Table 3:** Limits of agreement for each cytokine/chemokine tested.

Analyte	Round 1				Round 2		Best perfomance
	FlowCytomix™	Bio-Rad®	Invitrogen™	Millipore™	INV-MAG	BD™ CBA	
EGF	.	.	.	.	0,71–1,36	.	INV-MAG
EOTAXIN	.	.	–	.	–	.	–
FGF-Basic	.	.	.	.	0.66–1.81	.	INV-MAG
G-CSF	.	0.70–1.51*	.	.	0.56–1.47	.	INV-MAG
GM-CSF	.	**0.83–1.20**	0.30–1.93	.	**0.85–1.10**	.	INV-MAG
HGF	.	.	.	.	**0.73–1.31**	.	INV-MAG
IFN-α	.	.	0.23–1.92	.	0.64–1.66	.	INV-MAG
IFN-γ	0.64–1.66	0.62–1.71	0.25–5.05	**0.73–1.28**	–	–	Millipore™
IL-10	0.75–1.66*	0.76–1.56†	0.15–7.68	**0.84–1.28**	**0.86–1.27** * ‡	.	INV-MAG
IL-12	–	0.40–1.97	0.19–9.29	0.80–1.46	**0.64–1.16** *	.	INV-MAG
IL-13	–	0.65–1.66	–	.	0.54–1.62*	.	Bio-Rad®
IL-15	.	.	–	.	0.76–1.53*	.	INV-MAG
IL-17	–	0.74–1.54*	–	–	0.31–2.06	.	INV-MAG
IL-1β	0.64–1.74	0.72–1.47	0.46–2.71	**0.77–1.27** †	**0.88–1.23** * ‡	.	INV-MAG
IL-1Ra	.	.	0.45–2.75	.	0.57–1.66*	.	INV-MAG
IL-2	–	0.58–1.63	–	**0.83–1.18**	0.67–1.94	0.61–1.53	Millipore™
IL-22	–	.	.	.	.	.	–
IL-2R	.	.	0.48–2.23†	.	0.64–1.45	.	INV-MAG
IL-4	–	0.51–1.62*	–	0.61–1.61	0.27–1.47	0.42–1.60	INV-MAG
IL-5	–	0.52–2.78	–	–	–	.	Bio-Rad®
IL-6	0.68–1.52	0.72–1.40	0.51–2.43	**0.77–1.25** *	**0.89–1.18** * †	.	INV-MAG
IL-7	.	0.38–2.91*	.	.	**1.11–1.36** ‡	.	Bio-Rad®
IL-8	0.58–1.57*	**0.79–1.15**	0.47–2.40†	**0.81–1.14**	**0.93–1.17**	.	INV-MAG
IP-10	.	.	0.45–2.52	.	**0.79–1.36**	.	INV-MAG
MCP-1	.	0.73–1.45	0.39–2.30	.	**0.81–1.28**	.	INV-MAG
MIG	.	.	–	.	0.58–1.59*	.	INV-MAG
MIP-1α	.	.	0.46–2.65	.	**0.80–1.22** *	.	INV-MAG
MIP-1β	.	0.72–1.37†	0.47–2.30	.	0.69–1.39*	.	Bio-Rad
RANTES	.	.	0.49–2.17	.	**0.73–1.33**	.	INV-MAG
TNF	0.62–1.97†	0.77–1.56‡	0.31–7.56	**0.82–1.22**	0.58–1.45	.	Millipore™
TNF-β	–	.	.	**0.81–1.09**	.	.	Millipore™
VEGF	.	.	.	.	0.70–1.47†	.	INV-MAG
N° analytes with acceptable limits of agreement/total analytes (%)	0/14 (0.0%)	2/17 (11.8%)	0/25 (0.0%)	8/13 (62%)	12/30 (40.0%)	0/3 (0.0%)	Millipore

When differences are <30% the limits of agreement are considered acceptable and are highlighted in bold.

. (Cytokine/chemokine not tested).

– (less than 8.1% of both readings in range).

* Constant variance p value <0.05.

† Constant ratio p value <0.05.

‡ Ratio is 1 p value <0.05.

INV-MAG: Invitrogen™ kit with magnetic beads.

In general we found that the proportions of both readings in range were very low (Table 2) as in most analytes less than 75% of both duplicates were in range independently of the kit. This was mainly due to low cytokine and chemokine concentrations in supernatant samples that were below the limits of detection (e.g. IL-2), although in some cases the analyte concentration was over the accurate range (e.g. IL-8 and IL-6 for Invitrogen™). This was expected as in our previous malaria studies antigen-specific responses in children were rather low [15], [22].

The FlowCytomix™ kit had low percentages of both readings in range with the exception of IL-6 and IL-8 that had >75% of readings in range (Table 2), although the reproducibility among duplicates was poorer and none of the cytokines/chemokines had acceptable limits of agreement (Table 3). The Bio-Rad® kit had a higher proportion of both readings in range for overall cytokines/chemokines being >75% for 35% of the cytokines/chemokines tested (G-CSF, GM-CSF, IL-10, IL-1β, IL-4 and IL-7), although only 2 (GM-CSF and IL-8) out of 17 cytokines/chemokines (11.8%) had acceptable limits of agreement. The Invitrogen™ kit with polystyrene beads had low percentages of both readings in range (only RANTES had >75%) and low reproducibility (none of the cytokines/chemokines fulfilled the criteria of <30%). In contrast, the Invitrogen™ kit with magnetic beads (INV-MAG) detected many more cytokines in range (40% of cytokines/chemokines had >75% of both readings in range: EGF, HGF, IFN-α, IL-12, IL-13, IL-1Rα, IL-2R, IL-6, IP-10, MIP-1α, RANTES and VEGF), although some other cytokines/chemokines of interest did not perform as well (e.g. IFN-γ). The reproducibility of INV-MAG was greater than the other kits (40% of cytokines/chemokines had acceptable and tighter limits of agreement), but data could not be compared directly as the tests were done at different incubation time points. The Millipore™ kit did not show a high proportion of both readings in range with the exception of IL-1β and TNF that had >75% (15.38% of cytokines/chemokines tested), and overall showed a good reproducibility (62% of cytokines/chemokines tested fulfilled our criteria). The Enhanced Sensitivity CBA did not demonstrate to have more sensitivity than the INV-MAG kit and performed poorly.

Overall, we found that each kit performed differently in number of samples with duplicate readings in range and reproducibility depending on the cytokine/chemokine tested. In our hands, the performance of Luminex® methods was better than the flow cytometry methods in terms of proportion of samples in range and reproducibility, as previously observed [10]. These data differ from our previous observations that flow cytometry methods were more reproducible [15], but the Luminex® kits used in both studies were different. Within the Luminex® kits, the Invitrogen™ kit with non-magnetic beads had the poorer performance, whereas the Invitrogen™ kit with magnetic beads had the higher percentage of cytokines/chemokines with both readings in range, followed by the Bio-Rad® kit with magnetic beads. Regarding reproducibility, the Millipore™ kit had the highest percentage (60%) of cytokines/chemokines with acceptable limits of agreement, followed by INV-MAG™ kit (40%) that had tighter limits of agreement. However, we could only test 13 cytokines/chemokines with the Millipore™ kit, in contrast to the 30 cytokines/chemokines assessed with the INV-MAG. In general, kits with magnetic beads performed better than kits with polystyrene beads. Magnetic beads may decrease bead loss during washes and avoid plate clogging and leaks, which occasionally happen when performing non-magnetic bead assays. In addition, magnetic beads reduce non-specific binding, increasing reproducibility and robustness of the assay.

Several reasons may account for the variation in the performance of multiplex kits from different manufacturers as well as the heterogeneity in analytes within each kit. Probably the most important factor is the different antibody used for capture and detection of individual analytes by each manufacturer. This has also been described in a study comparing ELISA kits [23] in which it was suggested that the nature of the pairs of monoclonal antibodies employed in each ELISA kit did not permit comparable recognition of cytokines in samples. In addition, there may be differences among purified recombinant proteins used to generate the standard curves as well as in the assay buffers supplied by the manufacturers.

In addition to the individual cytokine performance in each kit, other factors may be important in the final selection of a multiplex cytokine profiling method. In Table 1 we compare some of these factors such as the number of cytokines measured within each kit, the volume of sample needed or the time and cost of the assays. Overall, Luminex®-based kits are the fastest, considering the incubation and reading time. Invitrogen™ was the most cost effective at the place and time the study was performed. Unfortunately, other commercially available kits such as Procarta® Cytokine Assays (Affymetrix®) or Fluorokine® MultiAnalayte Profiling Kits (R&D Systems®) could not be tested because they were not provided by the suppliers at the time the study was conducted.

### Conclusions

Differences in the number of samples detected in accurate range and reproducibility were observed depending on the method used and even the cytokine detected, although Luminex®-based kits with magnetic beads proved to be better. This suggests that the same kit should be used throughout a given study. When selecting the most suitable multiplex cytokine profiling method, a prioritization of analytes of interest should be done and other factors such as the number of cytokines measured with each kit, the time and cost of the assays may be also considered as they vary widely. Although it would be advisable to test different kits before starting any study and qualify or validate them, our data can help the decision making process to select the most appropriate multiplex suspension array kit in similar studies.

## Supporting Information

Figure S1 **Mean difference dot plot of EGF.** Disagreement plot shows the difference between the duplicates against the geometric mean of both values of a sample tested with Invitrogen™ Human Cytokine Magnetic 30-Plex Panel (INV-MAG). The middle line is the mean difference and the two extreme lines are the limits of agreement calculated by Bland-Altman test.
(PDF)

Figure S2 **Mean difference dot plot of FGF-Basic.** Disagreement plot shows the difference between the duplicates against the geometric mean of both values of a sample tested with Invitrogen™ Human Cytokine Magnetic 30-Plex Panel (INV-MAG). The middle line is the mean difference and the two extreme lines are the limits of agreement calculated by Bland-Altman test.
(PDF)

Figure S3 **Mean difference dot plots of G-CSF for each kit tested.** Disagreement plots show the difference between the duplicates against the geometric mean of both values of a sample tested with A) Bio-Rad® Bio-Plex Pro™ Human Cytokine Plex Assay (Bio-Rad), and B) Invitrogen™ Human Cytokine Magnetic 30-Plex Panel (INV-MAG). The middle line is the mean difference and the two extreme lines are the limits of agreement calculated by Bland-Altman test.
(PDF)

Figure S4 **Mean difference dot plots of GM-CSF for each kit tested.** Disagreement plots show the difference between the duplicates against the geometric mean of both values of a sample tested with A) Bio-Rad® Bio-Plex Pro™ Human Cytokine Plex Assay (Bio-Rad), B) Human Cytokine 25-Plex panel from Invitrogen™ (non-magnetic beads) and C) Invitrogen™ Human Cytokine Magnetic 30-Plex Panel (INV-MAG). The middle line is the mean difference and the two extreme lines are the limits of agreement calculated by Bland-Altman test.
(PDF)

Figure S5 **Mean difference dot plot of HGF.** Disagreement plot shows the difference between the duplicates against the geometric mean of both values of a sample tested with Invitrogen™ Human Cytokine Magnetic 30-Plex Panel (INV-MAG). The middle line is the mean difference and the two extreme lines are the limits of agreement calculated by Bland-Altman test.
(PDF)

Figure S6 **Mean difference dot plots of IFN-α for each kit tested.** Disagreement plots show the difference between the duplicates against the geometric mean of both values of a sample tested with A) Human Cytokine 25-Plex panel from Invitrogen™ (non-magnetic beads) and B) Invitrogen™ Human Cytokine Magnetic 30-Plex Panel (INV-MAG). The middle line is the mean difference and the two extreme lines are the limits of agreement calculated by Bland-Altman test.
(PDF)

Figure S7 **Mean difference dot plots of IFN-γ for each kit tested.** Disagreement plots show the difference between the duplicates against the geometric mean of both values of a sample tested with A) eBioscience® FlowCytomix™ (Bender), B) Bio-Rad® Bio-Plex Pro™ Human Cytokine Plex Assay (Bio-Rad), C) Human Cytokine 25-Plex panel from Invitrogen™ (non-magnetic beads), D) Invitrogen™ Human Cytokine Magnetic 30-Plex Panel (INV-MAG), and E) Millipore™ MILLIPLEX® MAP Plex Kit (Millipore). The middle line is the mean difference and the two extreme lines are the limits of agreement calculated by Bland-Altman test.
(PDF)

Figure S8 **Mean difference dot plots of IL-10 for each kit tested.** Disagreement plots show the difference between the duplicates against the geometric mean of both values of a sample tested with A) eBioscience® FlowCytomix™ (Bender), B) Bio-Rad® Bio-Plex Pro™ Human Cytokine Plex Assay (Bio-Rad), C) Human Cytokine 25-Plex panel from Invitrogen™ (non-magnetic beads), D) Invitrogen™ Human Cytokine Magnetic 30-Plex Panel (INV-MAG), and E) Millipore™ MILLIPLEX® MAP Plex Kit (Millipore). The middle line is the mean difference and the two extreme lines are the limits of agreement calculated by Bland-Altman test.
(PDF)

Figure S9 **Mean difference dot plots of IL-12 for each kit tested.** Disagreement plots show the difference between the duplicates against the geometric mean of both values of a sample tested with A) Bio-Rad® Bio-Plex Pro™ Human Cytokine Plex Assay (Bio-Rad), B) Human Cytokine 25-Plex panel from Invitrogen™ (non-magnetic beads), C) Invitrogen™ Human Cytokine Magnetic 30-Plex Panel (INV-MAG), and D) Millipore™ MILLIPLEX® MAP Plex Kit (Millipore). The middle line is the mean difference and the two extreme lines are the limits of agreement calculated by Bland-Altman test.
(PDF)

Figure S10 **Mean difference dot plots of IL-13 for each kit tested.** Disagreement plots show the difference between the duplicates against the geometric mean of both values of a sample tested with A) eBioscience® FlowCytomix™ (Bender), B) Bio-Rad® Bio-Plex Pro™ Human Cytokine Plex Assay (Bio-Rad) and C) Invitrogen™ Human Cytokine Magnetic 30-Plex Panel (INV-MAG). The middle line is the mean difference and the two extreme lines are the limits of agreement calculated by Bland-Altman test.
(PDF)

Figure S11 **Mean difference dot plot of IL-15.** Disagreement plot shows the difference between the duplicates against the geometric mean of both values of a sample tested with Invitrogen™ Human Cytokine Magnetic 30-Plex Panel (INV-MAG). The middle line is the mean difference and the two extreme lines are the limits of agreement calculated by Bland-Altman test.
(PDF)

Figure S12 **Mean difference dot plots of IL-17 for each kit tested.** Disagreement plots show the difference between the duplicates against the geometric mean of both values of a sample tested with A) Bio-Rad® Bio-Plex Pro™ Human Cytokine Plex Assay (Bio-Rad) and B) Invitrogen™ Human Cytokine Magnetic 30-Plex Panel (INV-MAG). The middle line is the mean difference and the two extreme lines are the limits of agreement calculated by Bland-Altman test.
(PDF)

Figure S13 **Mean difference dot plots of IL-1β for each kit tested.** Disagreement plots show the difference between the duplicates against the geometric mean of both values of a sample tested with A) eBioscience® FlowCytomix™ (Bender), B) Bio-Rad® Bio-Plex Pro™ Human Cytokine Plex Assay (Bio-Rad), C) Human Cytokine 25-Plex panel from Invitrogen™ (non-magnetic beads), D) Invitrogen™ Human Cytokine Magnetic 30-Plex Panel (INV-MAG), and D) Millipore™ MILLIPLEX® MAP Plex Kit (Millipore). The middle line is the mean difference and the two extreme lines are the limits of agreement calculated by Bland-Altman test.
(PDF)

Figure S14 **Mean difference dot plots of IL-1RA for each kit tested.** Disagreement plots show the difference between the duplicates against the geometric mean of both values of a sample tested with A) Human Cytokine 25-Plex panel from Invitrogen™ (non-magnetic beads) and B) Invitrogen™ Human Cytokine Magnetic 30-Plex Panel (INV-MAG). The middle line is the mean difference and the two extreme lines are the limits of agreement calculated by Bland-Altman test.
(PDF)

Figure S15 **Mean difference dot plots of IL-2 for each kit tested.** Disagreement plots show the difference between the duplicates against the geometric mean of both values of a sample tested with A) BD™ Cytometric Bead Array Human Enhanced Sensitivity kit (BD CBA), B) Bio-Rad® Bio-Plex Pro™ Human Cytokine Plex Assay (Bio-Rad), C) Invitrogen™ Human Cytokine Magnetic 30-Plex Panel (INV-MAG), and D) Millipore™ MILLIPLEX® MAP Plex Kit (Millipore). The middle line is the mean difference and the two extreme lines are the limits of agreement calculated by Bland-Altman test.
(PDF)

Figure S16 **Mean difference dot plots of IL-2R for each kit tested.** Disagreement plots show the difference between the duplicates against the geometric mean of both values of a sample tested with A) Human Cytokine 25-Plex panel from Invitrogen™ (non-magnetic beads) and B) Invitrogen™ Human Cytokine Magnetic 30-Plex Panel (INV-MAG). The middle line is the mean difference and the two extreme lines are the limits of agreement calculated by Bland-Altman test.
(PDF)

Figure S17 **Mean difference dot plots of IL-4 for each kit tested.** Disagreement plots show the difference between the duplicates against the geometric mean of both values of a sample tested with A) BD™ Cytometric Bead Array Human Enhanced Sensitivity kit (BD CBA), B) Bio-Rad® Bio-Plex Pro™ Human Cytokine Plex Assay (Bio-Rad), C) Invitrogen™ Human Cytokine Magnetic 30-Plex Panel (INV-MAG), and D) Millipore™ MILLIPLEX® MAP Plex Kit (Millipore). The middle line is the mean difference and the two extreme lines are the limits of agreement calculated by Bland-Altman test.
(PDF)

Figure S18 **Mean difference dot plots of IL-5 for each kit tested.** Disagreement plots show the difference between the duplicates against the geometric mean of both values of a sample tested with A) Bio-Rad® Bio-Plex Pro™ Human Cytokine Plex Assay (Bio-Rad), and B) Millipore™ MILLIPLEX® MAP Plex Kit (Millipore). The middle line is the mean difference and the two extreme lines are the limits of agreement calculated by Bland-Altman test.
(PDF)

Figure S19 **Mean difference dot plots of IL-6 for each kit tested.** Disagreement plots show the difference between the duplicates against the geometric mean of both values of a sample tested with A) eBioscience® FlowCytomix™ (Bender), B) Bio-Rad® Bio-Plex Pro™ Human Cytokine Plex Assay (Bio-Rad), C) Human Cytokine 25-Plex panel from Invitrogen™ (non-magnetic beads), D) Invitrogen™ Human Cytokine Magnetic 30-Plex Panel (INV-MAG), and E) Millipore™ MILLIPLEX® MAP Plex Kit (Millipore). The middle line is the mean difference and the two extreme lines are the limits of agreement calculated by Bland-Altman test.
(PDF)

Figure S20 **Mean difference dot plots of IL-7 for each kit tested.** Disagreement plots show the difference between the duplicates against the geometric mean of both values of a sample tested with A) Bio-Rad® Bio-Plex Pro™ Human Cytokine Plex Assay (Bio-Rad) and B) Invitrogen™ Human Cytokine Magnetic 30-Plex Panel (INV-MAG). The middle line is the mean difference and the two extreme lines are the limits of agreement calculated by Bland-Altman test.
(PDF)

Figure S21 **Mean difference dot plots of IL-8 for each kit tested.** Disagreement plots show the difference between the duplicates against the geometric mean of both values of a sample tested with A) eBioscience® FlowCytomix™ (Bender), B) Bio-Rad® Bio-Plex Pro™ Human Cytokine Plex Assay (Bio-Rad), C) Human Cytokine 25-Plex panel from Invitrogen™ (non-magnetic beads), D) Invitrogen™ Human Cytokine Magnetic 30-Plex Panel (INV-MAG), and E) Millipore™ MILLIPLEX® MAP Plex Kit (Millipore). The middle line is the mean difference and the two extreme lines are the limits of agreement calculated by Bland-Altman test.
(PDF)

Figure S22 **Mean difference dot plots of IP-10 for each kit tested.** Disagreement plots show the difference between the duplicates against the geometric mean of both values of a sample tested with A) Human Cytokine 25-Plex panel from Invitrogen™ (non-magnetic beads) and B) Invitrogen™ Human Cytokine Magnetic 30-Plex Panel (INV-MAG). The middle line is the mean difference and the two extreme lines are the limits of agreement calculated by Bland-Altman test.
(PDF)

Figure S23 **Mean difference dot plots of MCP-1 for each kit tested.** Disagreement plots show the difference between the duplicates against the geometric mean of both values of a sample tested with A) Bio-Rad® Bio-Plex Pro™ Human Cytokine Plex Assay (Bio-Rad) B) Human Cytokine 25-Plex panel from Invitrogen™ (non-magnetic beads) and C) Invitrogen™ Human Cytokine Magnetic 30-Plex Panel (INV-MAG). The middle line is the mean difference and the two extreme lines are the limits of agreement calculated by Bland-Altman test.
(PDF)

Figure S24 **Mean difference dot plot of MIG.** Disagreement plots show the difference between the duplicates against the geometric mean of both values of a sample tested with Invitrogen™ Human Cytokine Magnetic 30-Plex Panel (INV-MAG). The middle line is the mean difference and the two extreme lines are the limits of agreement calculated by Bland-Altman test.
(PDF)

Figure S25 **Mean difference dot plots of MIP-1α for each kit tested.** Disagreement plots show the difference between the duplicates against the geometric mean of both values of a sample tested with A) Human Cytokine 25-Plex panel from Invitrogen™ (non-magnetic beads) and B) Invitrogen™ Human Cytokine Magnetic 30-Plex Panel (INV-MAG). The middle line is the mean difference and the two extreme lines are the limits of agreement calculated by Bland-Altman test.
(PDF)

Figure S26 **Mean difference dot plots of MIP-1β for each kit tested.** Disagreement plots show the difference between the duplicates against the geometric mean of both values of a sample tested with A) Bio-Rad® Bio-Plex Pro™ Human Cytokine Plex Assay (Bio-Rad). B) Human Cytokine 25-Plex panel from Invitrogen™ (non-magnetic beads) and C) Invitrogen™ Human Cytokine Magnetic 30-Plex Panel (INV-MAG). The middle line is the mean difference and the two extreme lines are the limits of agreement calculated by Bland-Altman test.
(PDF)

Figure S27 **Mean difference dot plots of RANTES for each kit tested.** Disagreement plots show the difference between the duplicates against the geometric mean of both values of a sample tested with A) Human Cytokine 25-Plex panel from Invitrogen™ (non-magnetic beads) and B) Invitrogen™ Human Cytokine Magnetic 30-Plex Panel (INV-MAG). The middle line is the mean difference and the two extreme lines are the limits of agreement calculated by Bland-Altman test.
(PDF)

Figure S28 **Mean difference dot plots of TNF-α for each kit tested.** Disagreement plots show the difference between the duplicates against the geometric mean of both values of a sample tested with A) eBioscience® FlowCytomix™ (Bender), B) Bio-Rad® Bio-Plex Pro™ Human Cytokine Plex Assay (Bio-Rad), C) Human Cytokine 25-Plex panel from Invitrogen™ (non-magnetic beads), D) Invitrogen™ Human Cytokine Magnetic 30-Plex Panel (INV-MAG), and E) Millipore™ MILLIPLEX® MAP Plex Kit (Millipore). The middle line is the mean difference and the two extreme lines are the limits of agreement calculated by Bland-Altman test.
(PDF)

Figure S29 **Mean difference dot plot of TNF-β.** Disagreement plots show the difference between the duplicates against the geometric mean of both values of a sample tested with Millipore™ MILLIPLEX® MAP Plex Kit (Millipore). The middle line is the mean difference and the two extreme lines are the limits of agreement calculated by Bland-Altman test.
(PDF)

Figure S30 **Mean difference dot plot of VEGF.** Disagreement plots show the difference between the duplicates against the geometric mean of both values of a sample tested with Invitrogen™ Human Cytokine Magnetic 30-Plex Panel (INV-MAG). The middle line is the mean difference and the two extreme lines are the limits of agreement calculated by Bland-Altman test.
(PDF)
