# Supplementary material for: Performance of Multiplex Commercial Kits to Quantify Cytokine and Chemokine Responses in Culture Supernatants from Plasmodium falciparum Stimulations
Source: PLoS One. 2013 Jan 2;8(1):e52587. doi: 10.1371/journal.pone.0052587 (PMC3534665; doi:10.1371/journal.pone.0052587)

Figure S2

	parameter	value
1	Cytokine	FGF-Basic
2	Vendor	INV_MAG
3	Samples included in this agreement	19
4	Proportion of both readings in range	47.5
5	Limits of agreement	0.66 to 1.81
6	Constant variance p.value	0.062
7	Constant ratio p.value	0.170
8	Ratio is 1 p.value	0.155

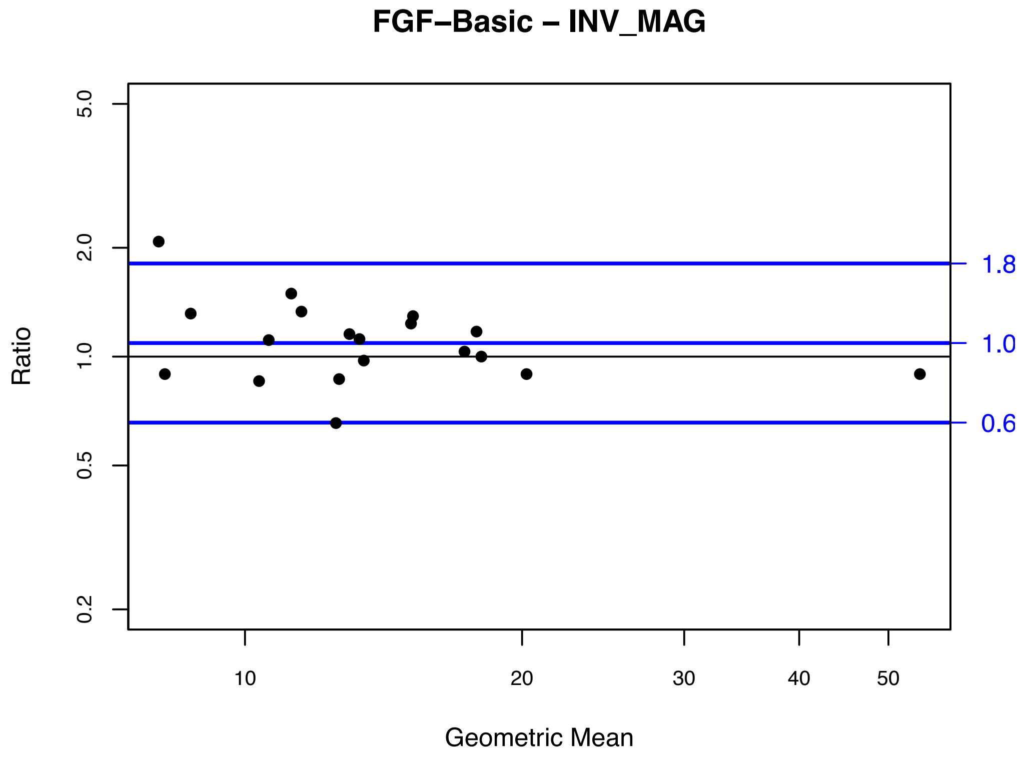

Supplement: Figure S2 — Mean difference dot plot of FGF-Basic. Disagreement plot shows the difference between the duplicates against the geometric mean of both values of a sample tested with Invitrogen™ Human Cytokine Magnetic 30-Plex Panel (INV-MAG). The middle line is the mean difference and the two extreme lines are the limits of agreement calculated by Bland-Altman test. (PDF) [file pone.0052587.s002.pdf]
