# Supplementary material for: Performance of Multiplex Commercial Kits to Quantify Cytokine and Chemokine Responses in Culture Supernatants from Plasmodium falciparum Stimulations
Source: PLoS One. 2013 Jan 2;8(1):e52587. doi: 10.1371/journal.pone.0052587 (PMC3534665; doi:10.1371/journal.pone.0052587)

Figure S3

A

	parameter	value
1	Cytokine	G-CSF
2	Vendor	Bio-Rad
3	Samples included in this agreement	32
4	Proportion of both readings in range	86.5
5	Limits of agreement	0.70 to 1.51
6	Constant variance p.value	0.003
7	Constant ratio p.value	0.249
8	Ratio is 1 p.value	0.454

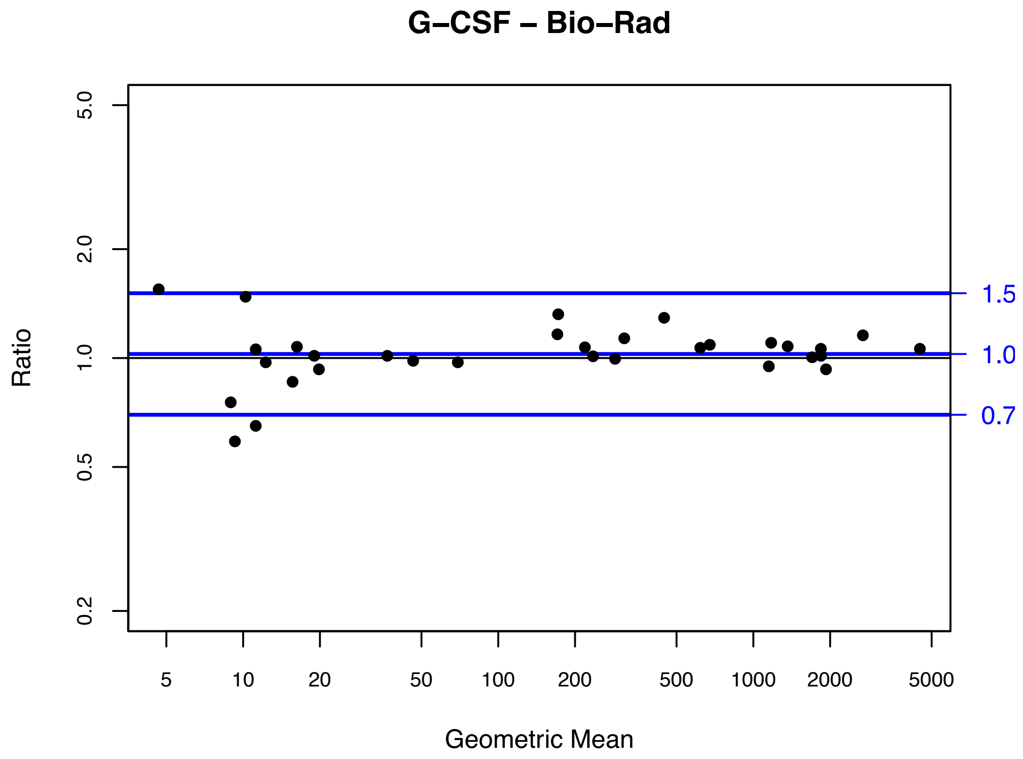

B

	parameter	value
1	Cytokine	G-CSF
2	Vendor	INV_MAG
3	Samples included in this agreement	26
4	Proportion of both readings in range	65.0
5	Limits of agreement	0.56 to 1.47
6	Constant variance p.value	0.096
7	Constant ratio p.value	0.143
8	Ratio is 1 p.value	0.059

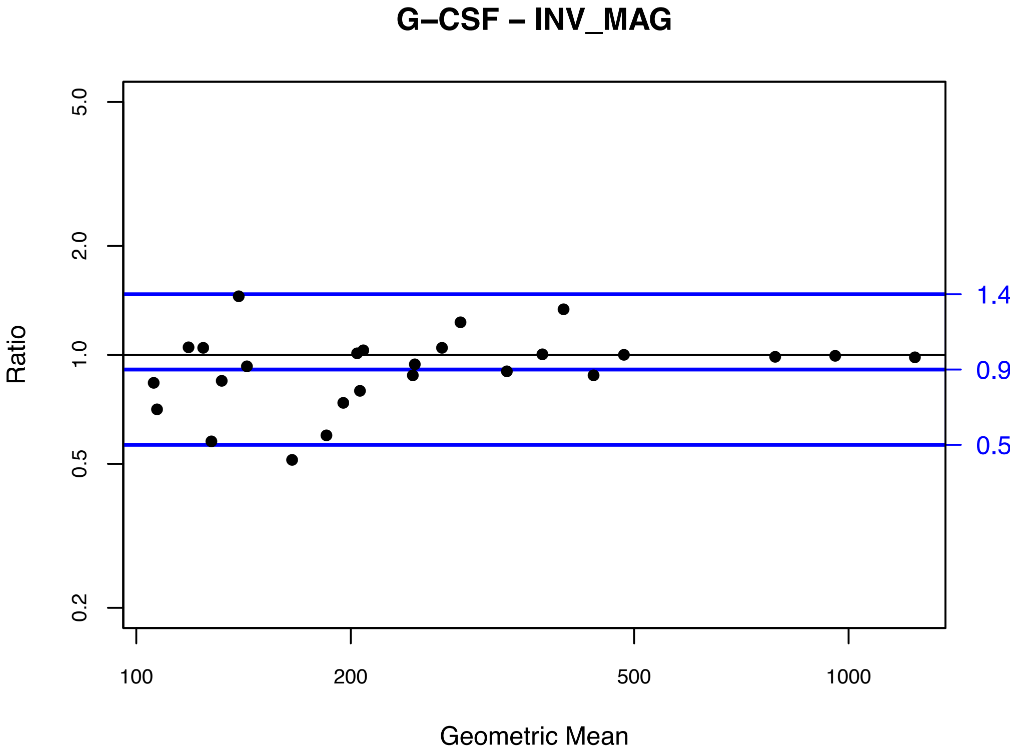

Supplement: Figure S3 — Mean difference dot plots of G-CSF for each kit tested. Disagreement plots show the difference between the duplicates against the geometric mean of both values of a sample tested with A) Bio-Rad® Bio-Plex Pro™ Human Cytokine Plex Assay (Bio-Rad), and B) Invitrogen™ Human Cytokine Magnetic 30-Plex Panel (INV-MAG). The middle line is the mean difference and the two extreme lines are the limits of agreement calculated by Bland-Altman test. (PDF) [file pone.0052587.s003.pdf]
