# Supplementary material for: Performance of Multiplex Commercial Kits to Quantify Cytokine and Chemokine Responses in Culture Supernatants from Plasmodium falciparum Stimulations
Source: PLoS One. 2013 Jan 2;8(1):e52587. doi: 10.1371/journal.pone.0052587 (PMC3534665; doi:10.1371/journal.pone.0052587)

Figure S4

A

	parameter	value
1	Cytokine	GM-CSF
2	Vendor	Bio-Rad
3	Samples included in this agreement	36
4	Proportion of both readings in range	97.3
5	Limits of agreement	0.83 to 1.20
6	Constant variance p.value	0.074
7	Constant ratio p.value	0.476
8	Ratio is 1 p.value	0.767

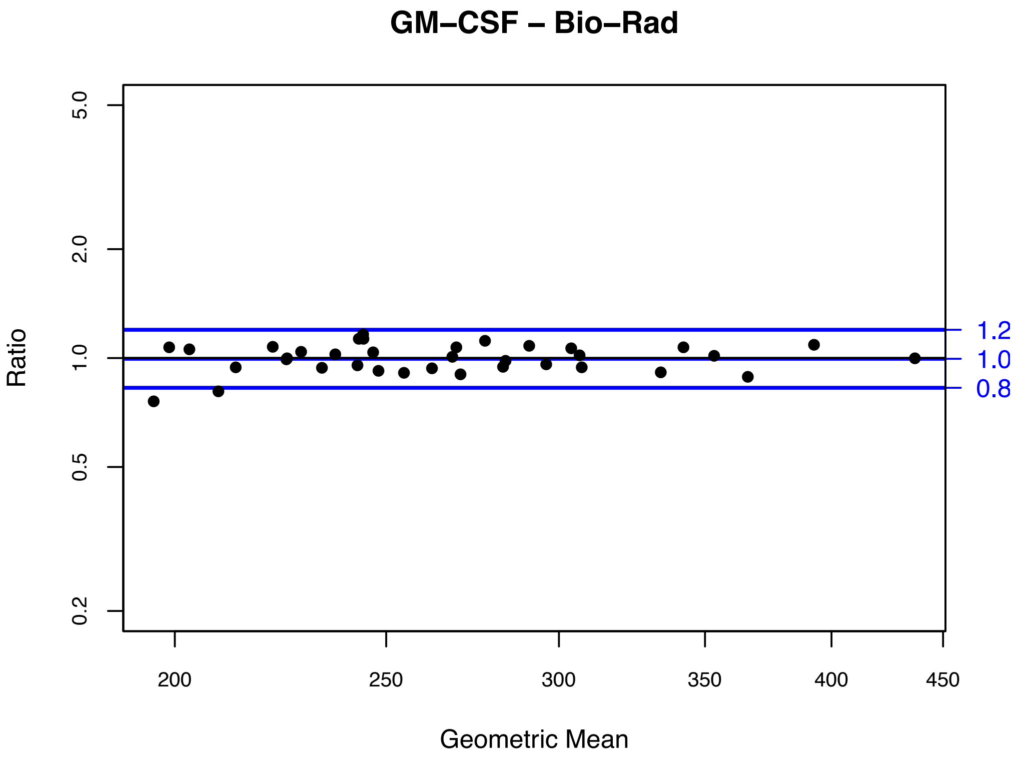

B

	parameter	value
1	Cytokine	GM-CSF
2	Vendor	Invitrogen
3	Samples included in this agreement	3
4	Proportion of both readings in range	8.1
5	Limits of agreement	0.30 to 1.93
6	Constant variance p.value	0.244
7	Constant ratio p.value	0.244
8	Ratio is 1 p.value	0.422

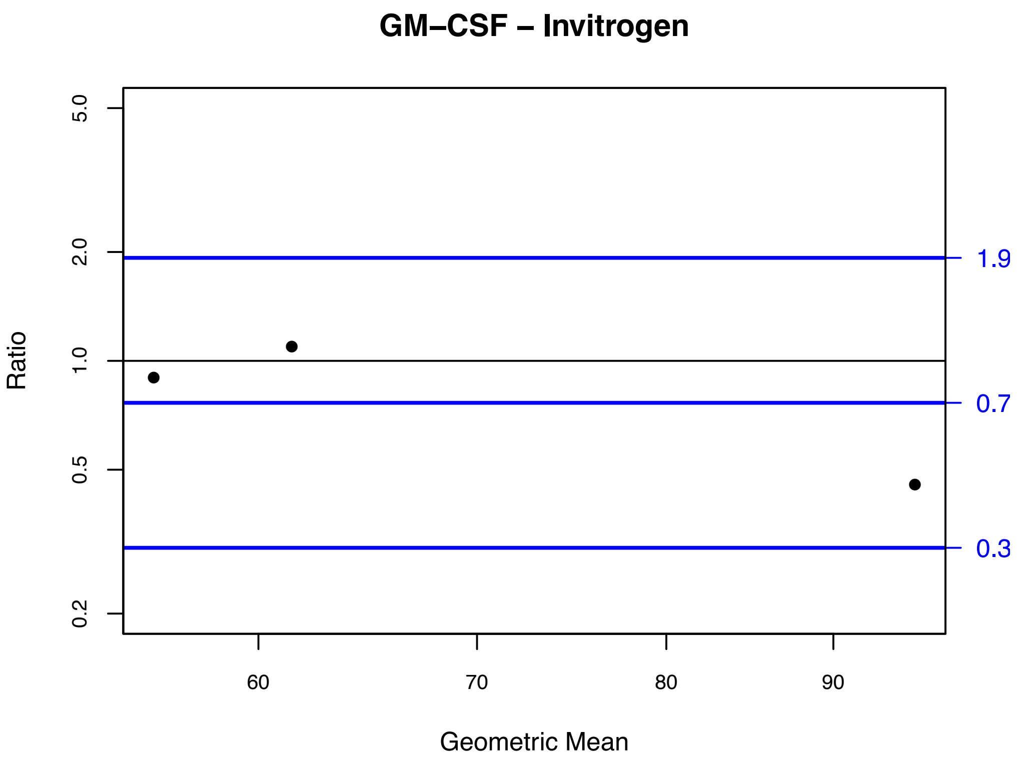

C

	parameter	value
1	Cytokine	GM-CSF
2	Vendor	INV_MAG
3	Samples included in this agreement	4
4	Proportion of both readings in range	10.0
5	Limits of agreement	0.85 to 1.10
6	Constant variance p.value	0.352
7	Constant ratio p.value	0.800
8	Ratio is 1 p.value	0.404

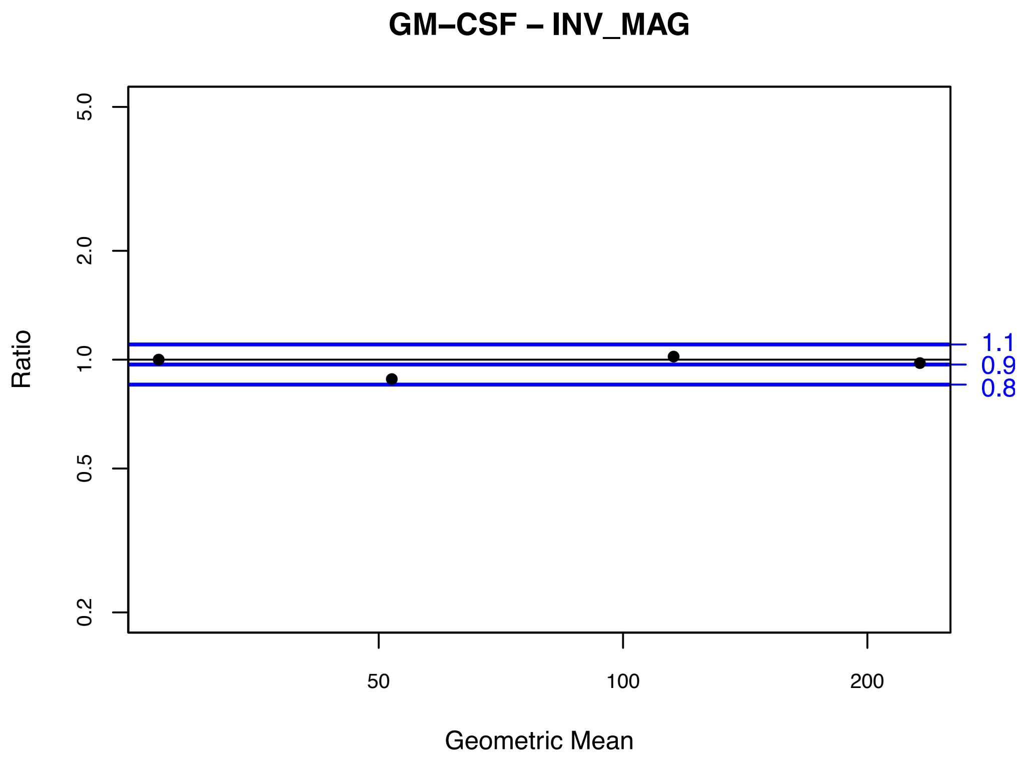

Supplement: Figure S4 — Mean difference dot plots of GM-CSF for each kit tested. Disagreement plots show the difference between the duplicates against the geometric mean of both values of a sample tested with A) Bio-Rad® Bio-Plex Pro™ Human Cytokine Plex Assay (Bio-Rad), B) Human Cytokine 25-Plex panel from Invitrogen™ (non-magnetic beads) and C) Invitrogen™ Human Cytokine Magnetic 30-Plex Panel (INV-MAG). The middle line is the mean difference and the two extreme lines are the limits of agreement calculated by Bland-Altman test. (PDF) [file pone.0052587.s004.pdf]
