# Supplementary material for: Performance of Multiplex Commercial Kits to Quantify Cytokine and Chemokine Responses in Culture Supernatants from Plasmodium falciparum Stimulations
Source: PLoS One. 2013 Jan 2;8(1):e52587. doi: 10.1371/journal.pone.0052587 (PMC3534665; doi:10.1371/journal.pone.0052587)

Figure S6

A

	parameter	value
1	Cytokine	IFN-alpha
2	Vendor	Invitrogen
3	Samples included in this agreement	3
4	Proportion of both readings in range	8.1
5	Limits of agreement	0.23 to 10.92
6	Constant variance p.value	0.280
7	Constant ratio p.value	0.637
8	Ratio is 1 p.value	0.494

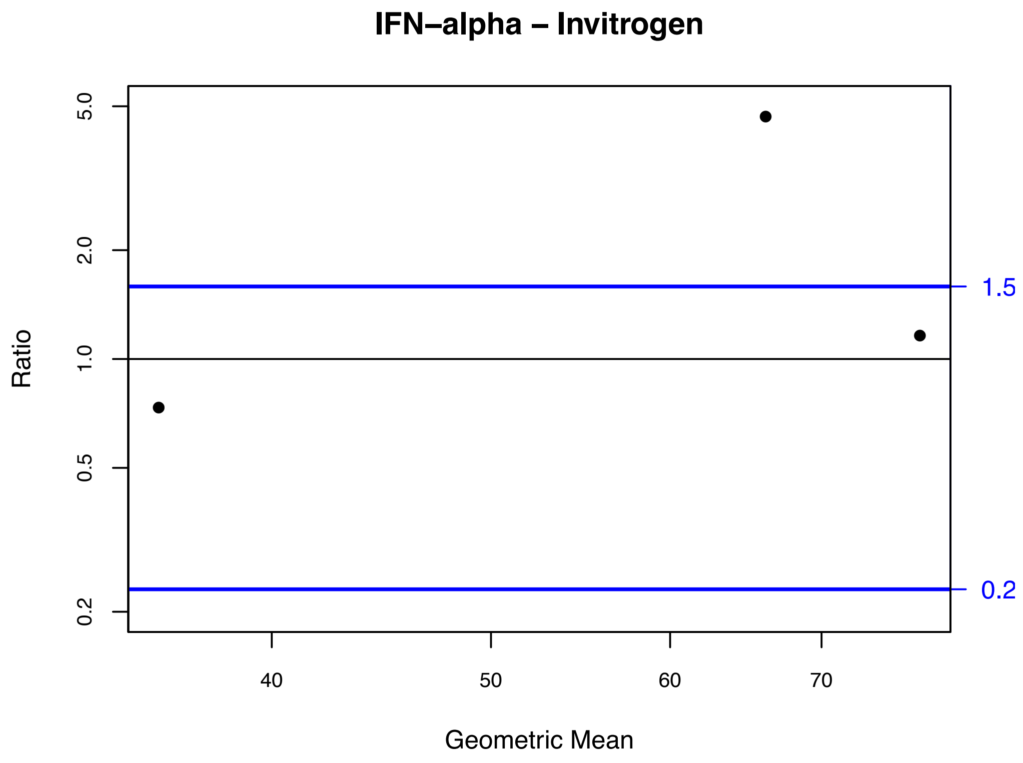

B

	parameter	value
1	Cytokine	IFN-alpha
2	Vendor	INV_MAG
3	Samples included in this agreement	35
4	Proportion of both readings in range	87.5
5	Limits of agreement	0.80 to 1.24
6	Constant variance p.value	0.230
7	Constant ratio p.value	0.599
8	Ratio is 1 p.value	0.917

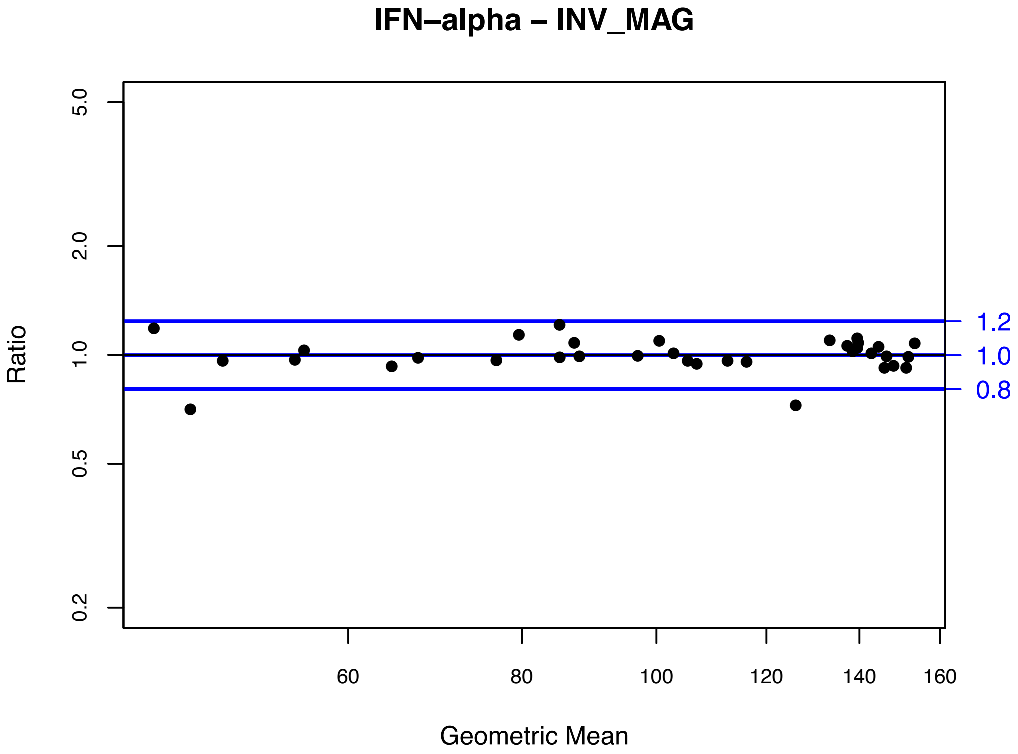

Supplement: Figure S6 — Mean difference dot plots of IFN-α for each kit tested. Disagreement plots show the difference between the duplicates against the geometric mean of both values of a sample tested with A) Human Cytokine 25-Plex panel from Invitrogen™ (non-magnetic beads) and B) Invitrogen™ Human Cytokine Magnetic 30-Plex Panel (INV-MAG). The middle line is the mean difference and the two extreme lines are the limits of agreement calculated by Bland-Altman test. (PDF) [file pone.0052587.s006.pdf]
