# Supplementary material for: Performance of Multiplex Commercial Kits to Quantify Cytokine and Chemokine Responses in Culture Supernatants from Plasmodium falciparum Stimulations
Source: PLoS One. 2013 Jan 2;8(1):e52587. doi: 10.1371/journal.pone.0052587 (PMC3534665; doi:10.1371/journal.pone.0052587)

Figure S7

A

	parameter	value
1	Cytokine	IFN-gamma
2	Vendor	Bender
3	Samples included in this agreement	7
4	Proportion of both readings in range	18.9
5	Limits of agreement	0.64 to 1.66
6	Constant variance p.value	0.281
7	Constant ratio p.value	0.066
8	Ratio is 1 p.value	0.766

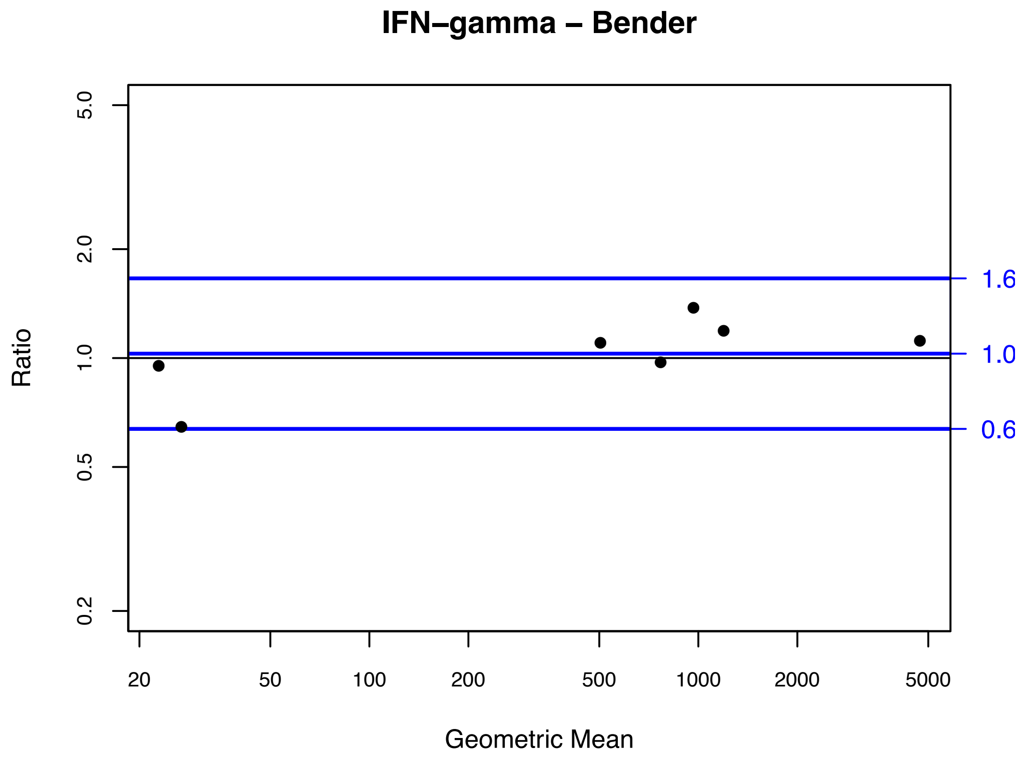

B

	parameter	value
1	Cytokine	IFN-gamma
2	Vendor	Bio-Rad
3	Samples included in this agreement	26
4	Proportion of both readings in range	70.3
5	Limits of agreement	0.62 to 1.71
6	Constant variance p.value	0.256
7	Constant ratio p.value	0.263
8	Ratio is 1 p.value	0.583

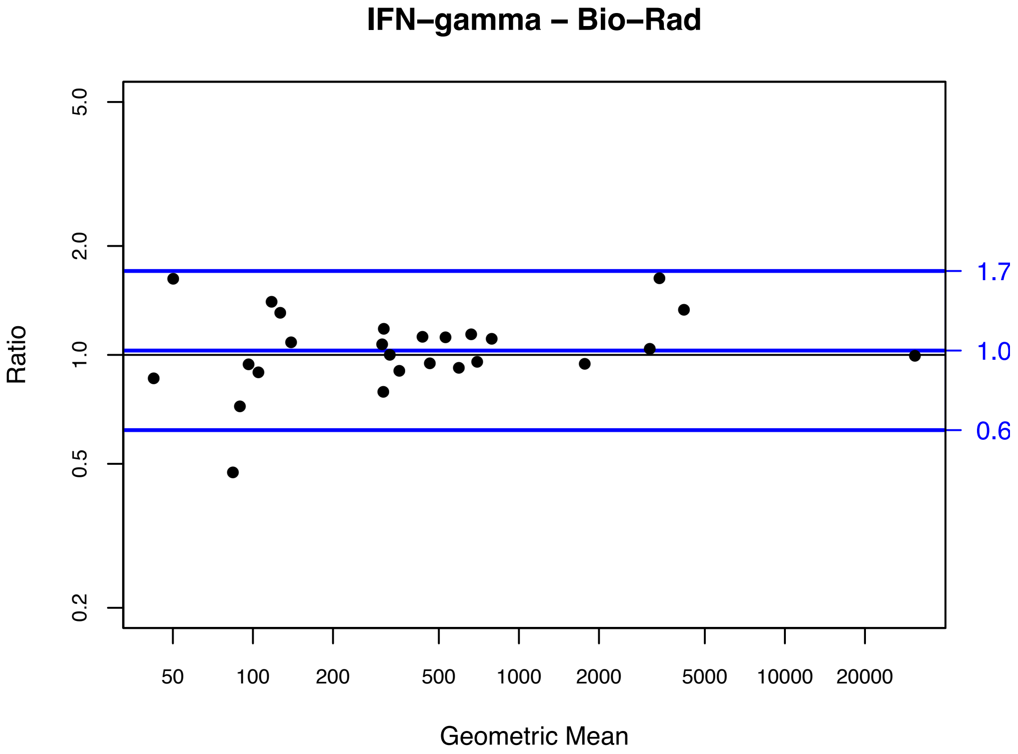

C

	parameter	value
1	Cytokine	IFN-gamma
2	Vendor	Invitrogen
3	Samples included in this agreement	4
4	Proportion of both readings in range	10.8
5	Limits of agreement	0.25 to 5.05
6	Constant variance p.value	0.899
7	Constant ratio p.value	0.436
8	Ratio is 1 p.value	0.763

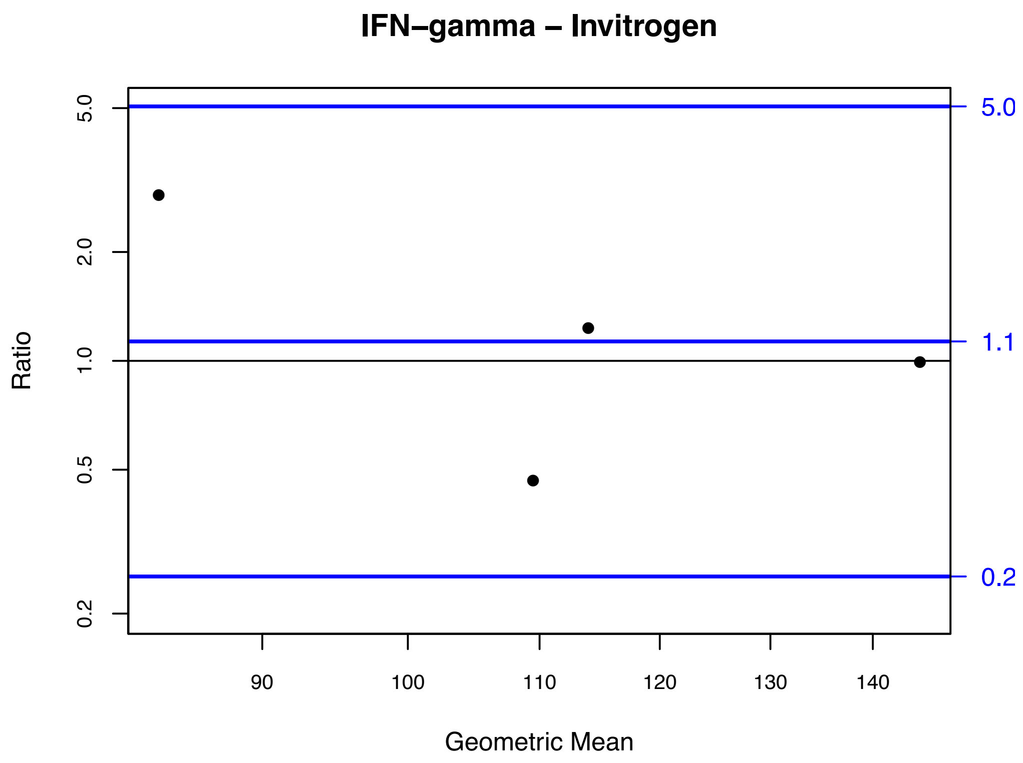

D

	parameter	value
1	Cytokine	IFN-gamma
2	Vendor	INV_MAG
3	Samples included in this agreement	2
4	Proportion of both readings in range	5.0
5	Limits of agreement	0.98 to 0.99
6	Constant variance p.value	NaN
7	Constant ratio p.value	NaN
8	Ratio is 1 p.value	0.059

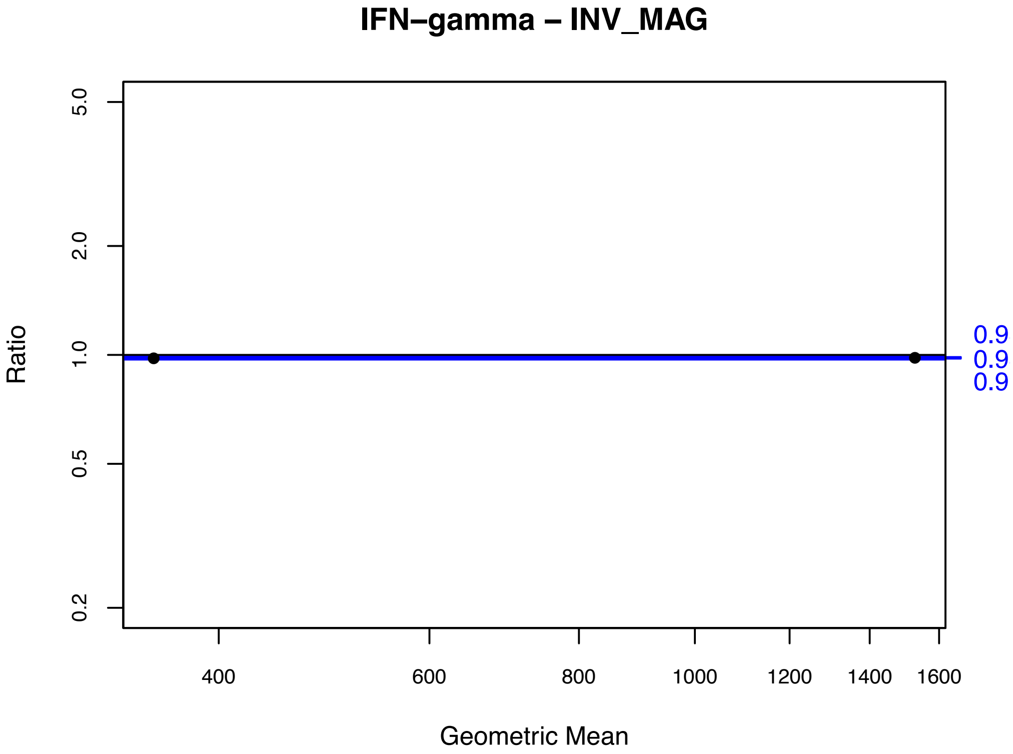

E

	parameter	value
1	Cytokine	IFN-gamma
2	Vendor	Millipore
3	Samples included in this agreement	9
4	Proportion of both readings in range	24.3
5	Limits of agreement	0.73 to 1.28
6	Constant variance p.value	0.141
7	Constant ratio p.value	0.829
8	Ratio is 1 p.value	0.526

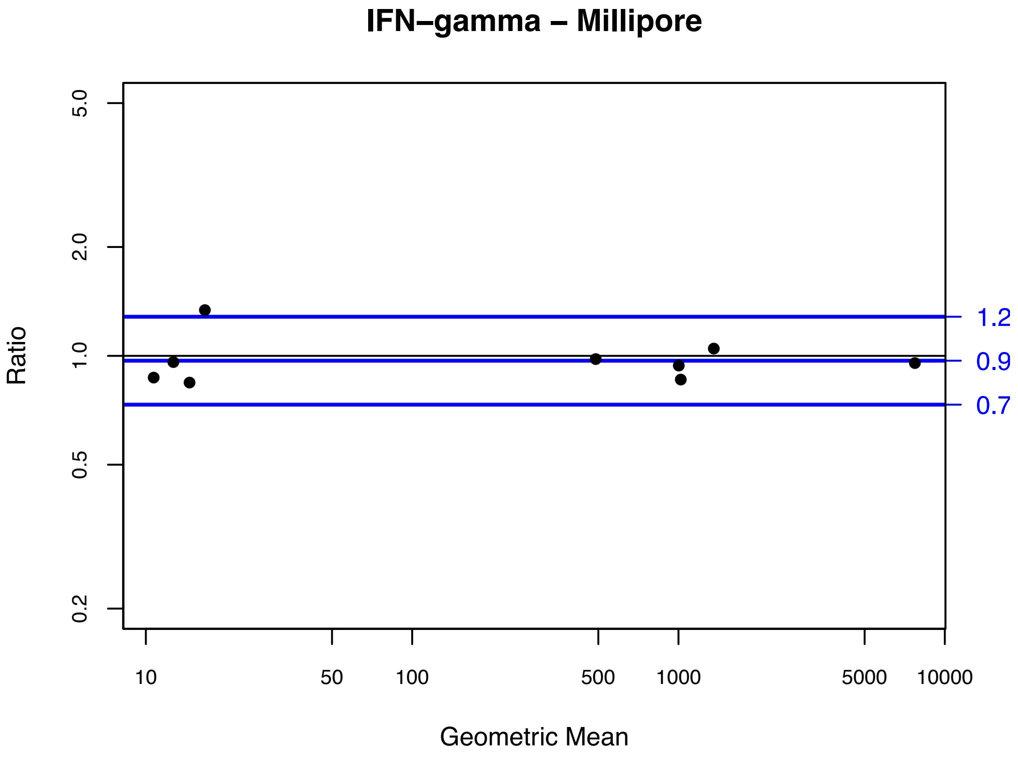

Supplement: Figure S7 — Mean difference dot plots of IFN-γ for each kit tested. Disagreement plots show the difference between the duplicates against the geometric mean of both values of a sample tested with A) eBioscience® FlowCytomix™ (Bender), B) Bio-Rad® Bio-Plex Pro™ Human Cytokine Plex Assay (Bio-Rad), C) Human Cytokine 25-Plex panel from Invitrogen™ (non-magnetic beads), D) Invitrogen™ Human Cytokine Magnetic 30-Plex Panel (INV-MAG), and E) Millipore™ MILLIPLEX® MAP Plex Kit (Millipore). The middle line is the mean difference and the two extreme lines are the limits of agreement calculated by Bland-Altman test. (PDF) [file pone.0052587.s007.pdf]
