# Supplementary material for: Performance of Multiplex Commercial Kits to Quantify Cytokine and Chemokine Responses in Culture Supernatants from Plasmodium falciparum Stimulations
Source: PLoS One. 2013 Jan 2;8(1):e52587. doi: 10.1371/journal.pone.0052587 (PMC3534665; doi:10.1371/journal.pone.0052587)

## Figure S8

### A

| | parameter | value |
|---|---|---|
| 1 | Cytokine | IL-10 |
| 2 | Vendor | Bender |
| 3 | Samples included in this agreement | 8 |
| 4 | Proportion of both readings in range | 21.6 |
| 5 | Limits of agreement | 0.75 to 1.66 |
| 6 | Constant variance p.value | 0.003 |
| 7 | Constant ratio p.value | 0.263 |
| 8 | Ratio is 1 p.value | 0.171 |

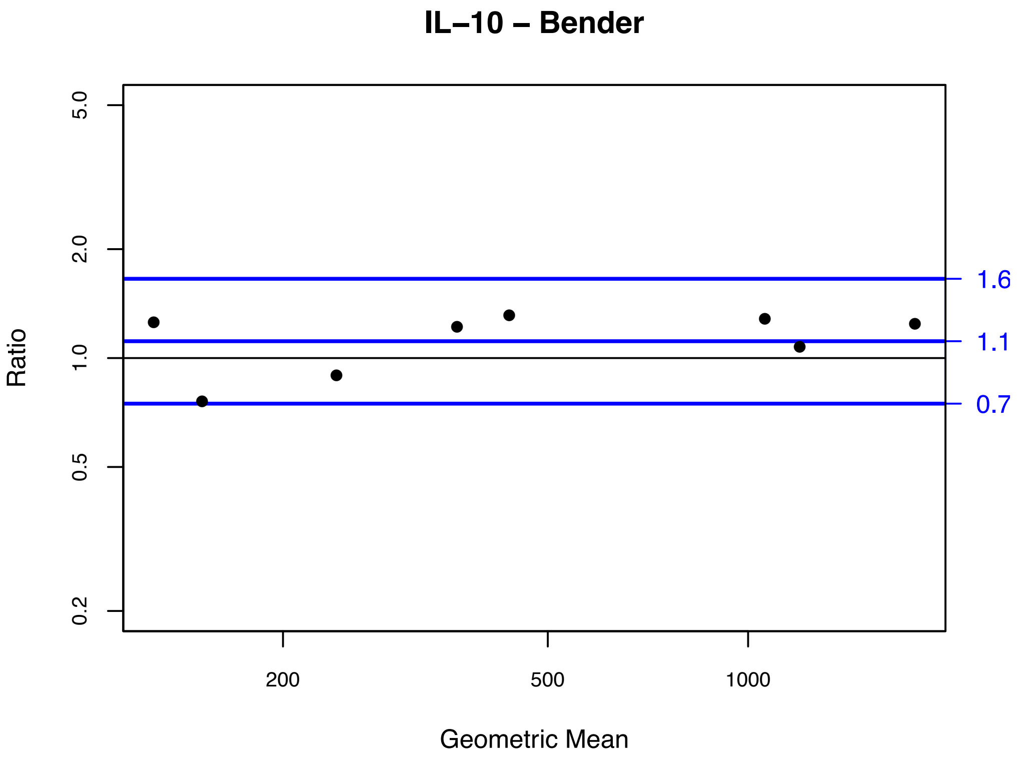

**B**

| | parameter | value |
|---|---|---|
| 1 | Cytokine | IL-10 |
| 2 | Vendor | Bio-Rad |
| 3 | Samples included in this agreement | 32 |
| 4 | Proportion of both readings in range | 86.5 |
| 5 | Limits of agreement | 0.76 to 1.56 |
| 6 | Constant variance p.value | 0.501 |
| 7 | Constant ratio p.value | 0.019 |
| 8 | Ratio is 1 p.value | 0.014 |

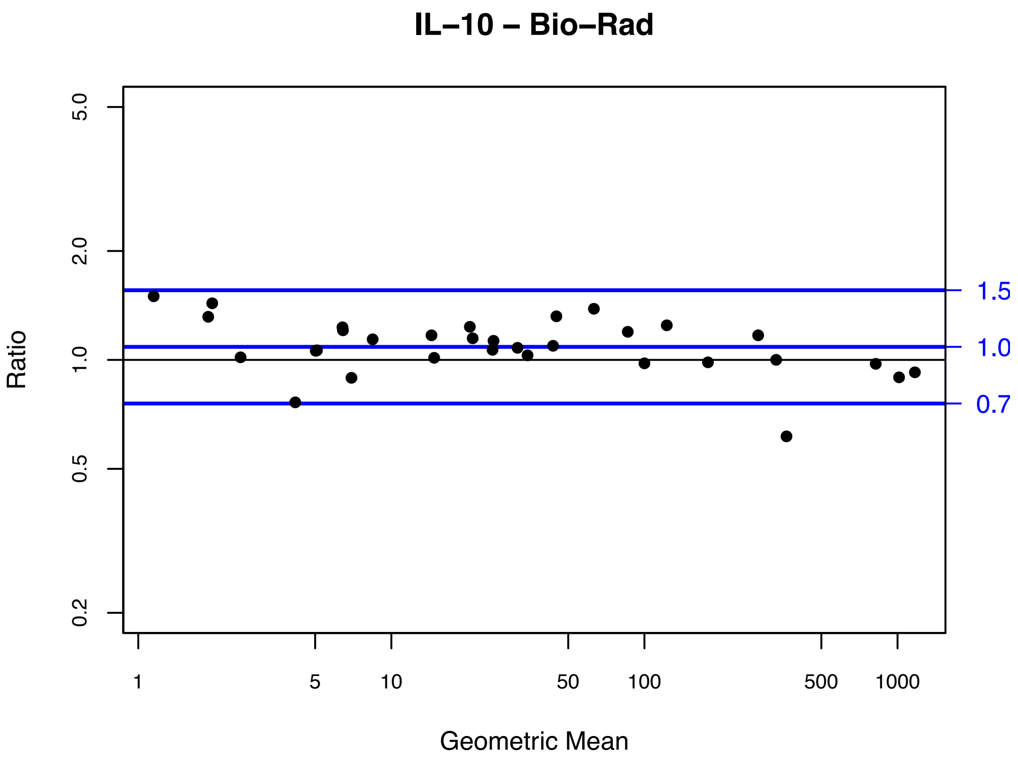

**C**

| | parameter | value |
|---|---|---|
| 1 | Cytokine | IL-10 |
| 2 | Vendor | Invitrogen |
| 3 | Samples included in this agreement | 4 |
| 4 | Proportion of both readings in range | 10.8 |
| 5 | Limits of agreement | 0.15 to 7.68 |
| 6 | Constant variance p.value | 0.268 |
| 7 | Constant ratio p.value | 0.064 |
| 8 | Ratio is 1 p.value | 0.914 |

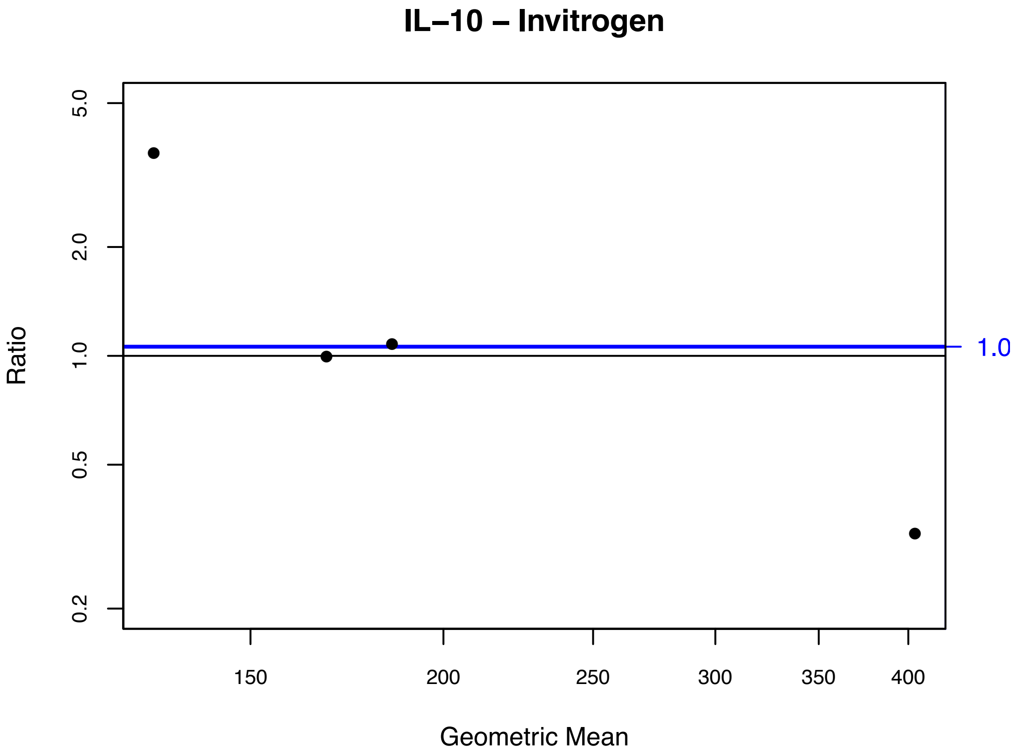

**D**

| | parameter | value |
|---|---|---|
| 1 | Cytokine | IL-10 |
| 2 | Vendor | INV_MAG |
| 3 | Samples included in this agreement | 25 |
| 4 | Proportion of both readings in range | 62.5 |
| 5 | Limits of agreement | 0.86 to 1.27 |
| 6 | Constant variance p.value | 0.004 |
| 7 | Constant ratio p.value | 0.290 |
| 8 | Ratio is 1 p.value | 0.035 |

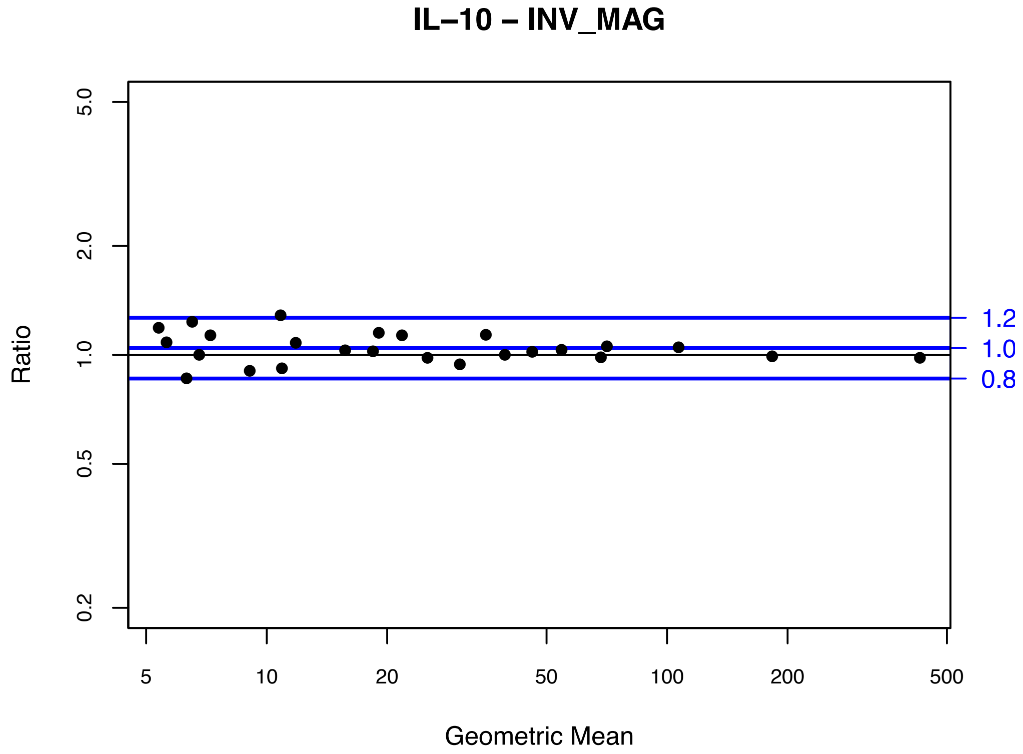

**E**

| | parameter | value |
|---|---|---|
| 1 | Cytokine | IL-10 |
| 2 | Vendor | Millipore |
| 3 | Samples included in this agreement | 23 |
| 4 | Proportion of both readings in range | 62.2 |
| 5 | Limits of agreement | 0.84 to 1.28 |
| 6 | Constant variance p.value | 0.177 |
| 7 | Constant ratio p.value | 0.132 |
| 8 | Ratio is 1 p.value | 0.147 |

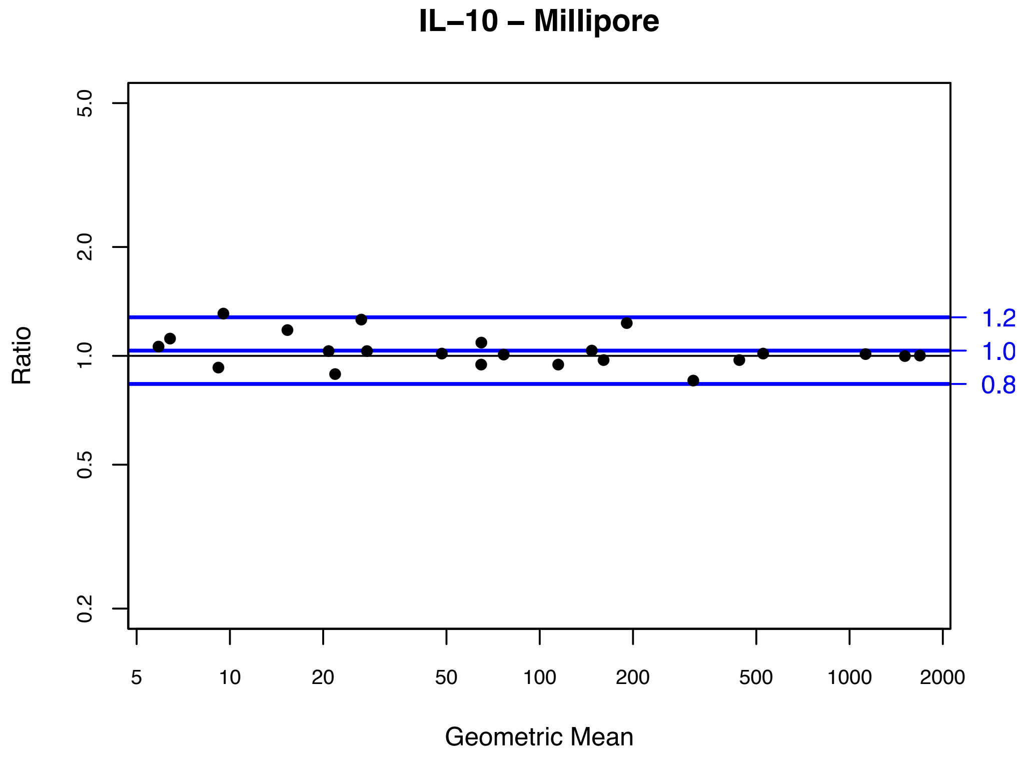

Supplement: Figure S8 — Mean difference dot plots of IL-10 for each kit tested. Disagreement plots show the difference between the duplicates against the geometric mean of both values of a sample tested with A) eBioscience® FlowCytomix™ (Bender), B) Bio-Rad® Bio-Plex Pro™ Human Cytokine Plex Assay (Bio-Rad), C) Human Cytokine 25-Plex panel from Invitrogen™ (non-magnetic beads), D) Invitrogen™ Human Cytokine Magnetic 30-Plex Panel (INV-MAG), and E) Millipore™ MILLIPLEX® MAP Plex Kit (Millipore). The middle line is the mean difference and the two extreme lines are the limits of agreement calculated by Bland-Altman test. (PDF) [file pone.0052587.s008.pdf]
