# Supplementary material for: Performance of Multiplex Commercial Kits to Quantify Cytokine and Chemokine Responses in Culture Supernatants from Plasmodium falciparum Stimulations
Source: PLoS One. 2013 Jan 2;8(1):e52587. doi: 10.1371/journal.pone.0052587 (PMC3534665; doi:10.1371/journal.pone.0052587)

Figure S9

A

	parameter	value
1	Cytokine	IL-12
2	Vendor	Bio-Rad
3	Samples included in this agreement	24
4	Proportion of both readings in range	64.9
5	Limits of agreement	0.40 to 1.97
6	Constant variance p.value	0.236
7	Constant ratio p.value	0.712
8	Ratio is 1 p.value	0.155

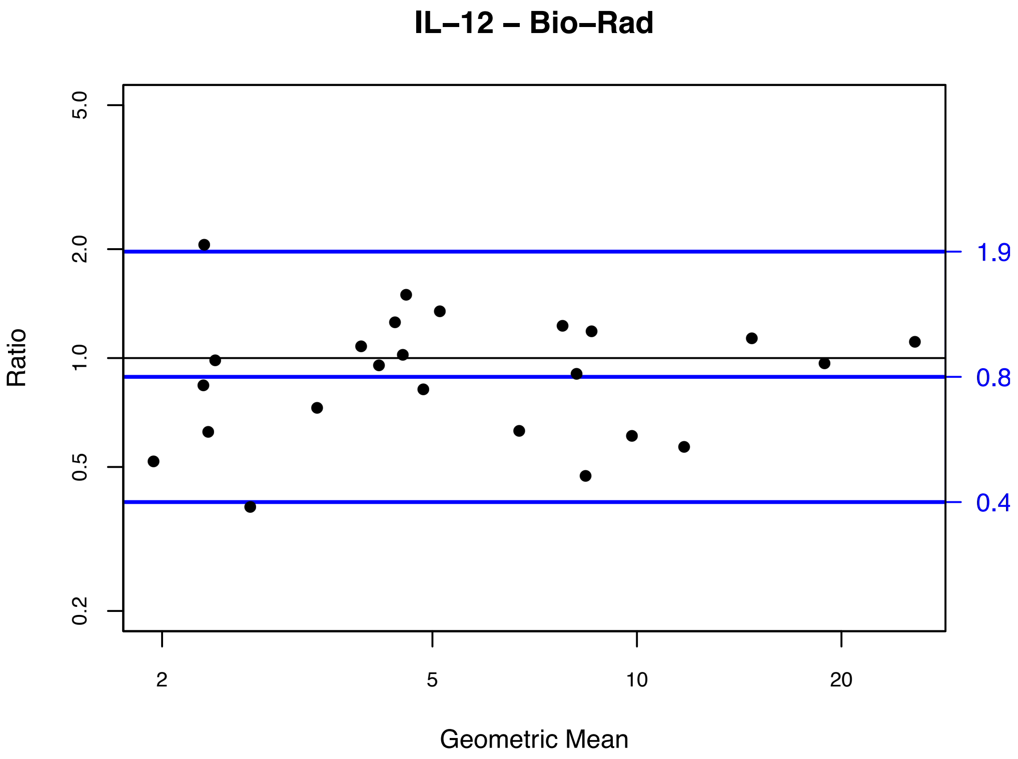

B

	parameter	value
1	Cytokine	IL-12
2	Vendor	Invitrogen
3	Samples included in this agreement	20
4	Proportion of both readings in range	54.1
5	Limits of agreement	0.19 to 9.29
6	Constant variance p.value	0.808
7	Constant ratio p.value	0.055
8	Ratio is 1 p.value	0.191

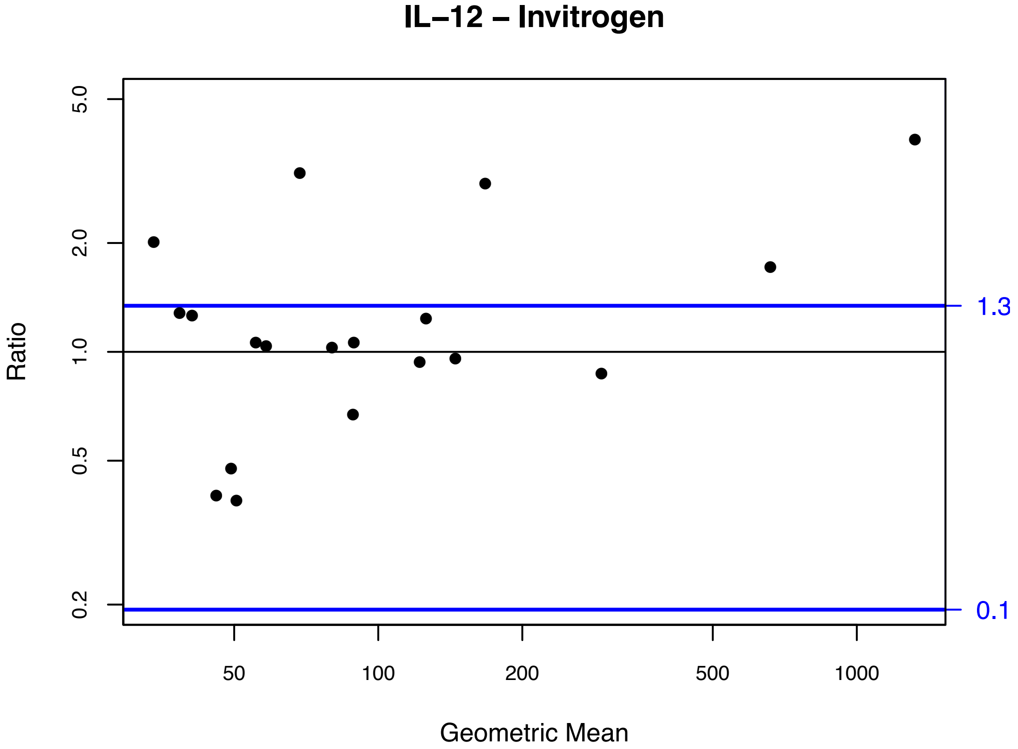

C

	parameter	value
1	Cytokine	IL-12
2	Vendor	INV_MAG
3	Samples included in this agreement	39
4	Proportion of both readings in range	97.5
5	Limits of agreement	0.64 to 1.60
6	Constant variance p.value	0.037
7	Constant ratio p.value	0.814
8	Ratio is 1 p.value	0.744

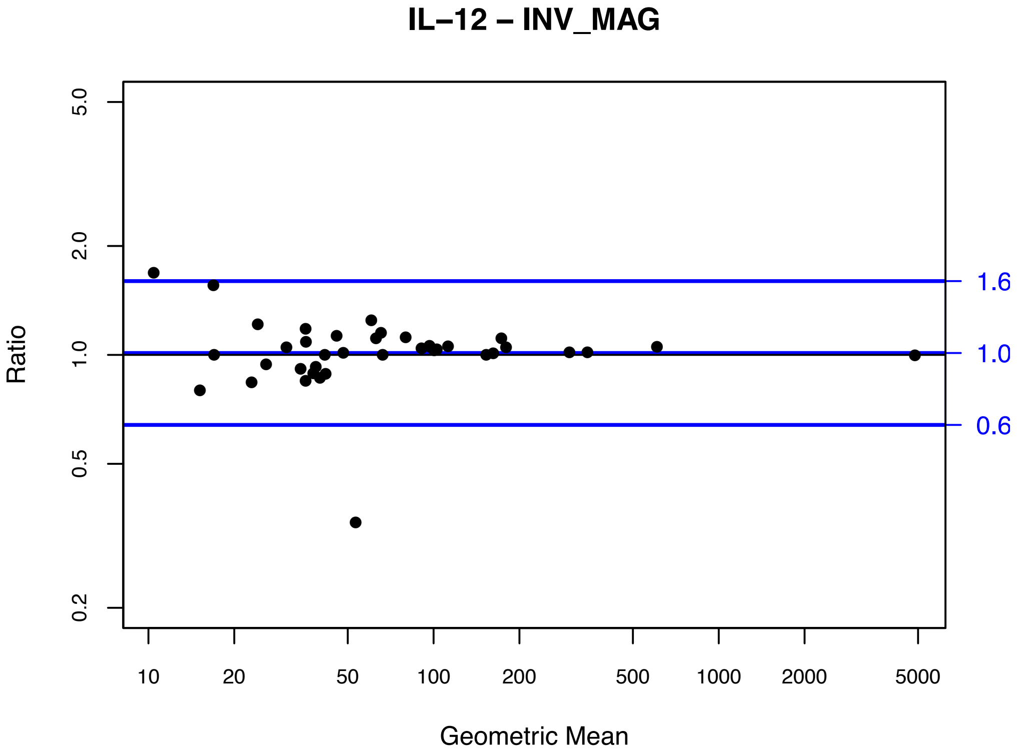

D

	parameter	value
1	Cytokine	IL-12
2	Vendor	Millipore
3	Samples included in this agreement	5
4	Proportion of both readings in range	13.5
5	Limits of agreement	0.80 to 1.46
6	Constant variance p.value	0.385
7	Constant ratio p.value	0.209
8	Ratio is 1 p.value	0.332

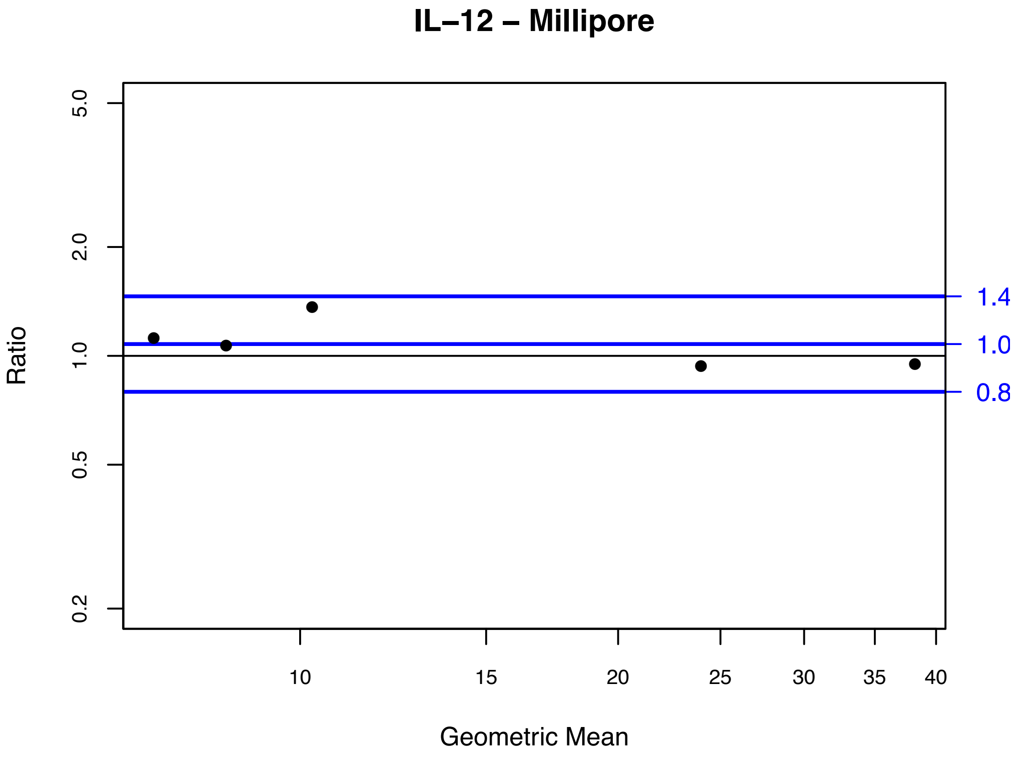

Supplement: Figure S9 — Mean difference dot plots of IL-12 for each kit tested. Disagreement plots show the difference between the duplicates against the geometric mean of both values of a sample tested with A) Bio-Rad® Bio-Plex Pro™ Human Cytokine Plex Assay (Bio-Rad), B) Human Cytokine 25-Plex panel from Invitrogen™ (non-magnetic beads), C) Invitrogen™ Human Cytokine Magnetic 30-Plex Panel (INV-MAG), and D) Millipore™ MILLIPLEX® MAP Plex Kit (Millipore). The middle line is the mean difference and the two extreme lines are the limits of agreement calculated by Bland-Altman test. (PDF) [file pone.0052587.s009.pdf]
