# Supplementary material for: Performance of Multiplex Commercial Kits to Quantify Cytokine and Chemokine Responses in Culture Supernatants from Plasmodium falciparum Stimulations
Source: PLoS One. 2013 Jan 2;8(1):e52587. doi: 10.1371/journal.pone.0052587 (PMC3534665; doi:10.1371/journal.pone.0052587)

Figure S10

A

	parameter	value
1	Cytokine	IL-13
2	Vendor	Bender
3	Samples included in this agreement	2
4	Proportion of both readings in range	5.4
5	Limits of agreement	0.88 to 1.28
6	Constant variance p.value	NaN
7	Constant ratio p.value	NaN
8	Ratio is 1 p.value	0.515

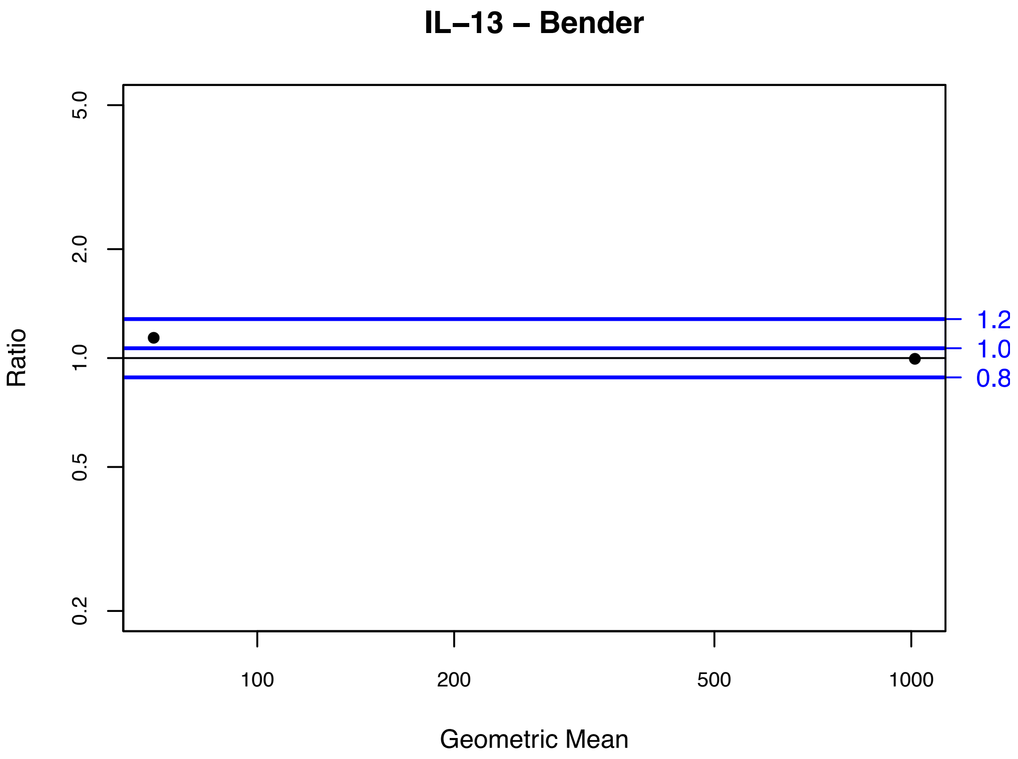

B

	parameter	value
1	Cytokine	IL-13
2	Vendor	Bio-Rad
3	Samples included in this agreement	25
4	Proportion of both readings in range	67.6
5	Limits of agreement	0.65 to 1.66
6	Constant variance p.value	0.279
7	Constant ratio p.value	0.444
8	Ratio is 1 p.value	0.446

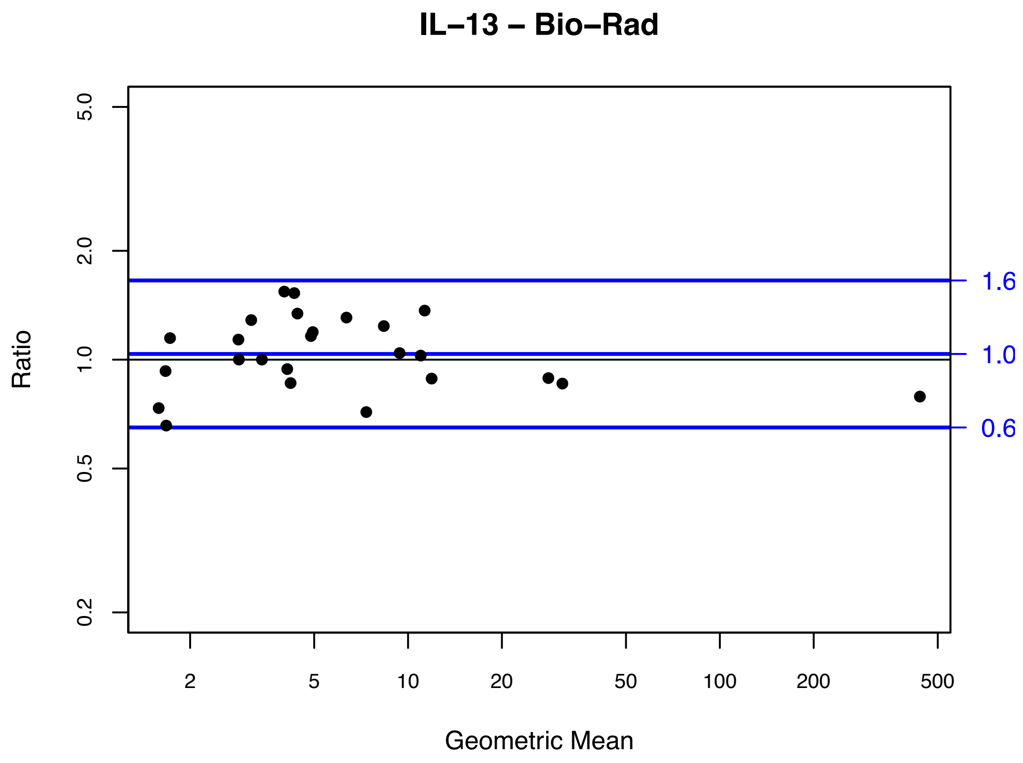

C

	parameter	value
1	Cytokine	IL-13
2	Vendor	INV_MAG
3	Samples included in this agreement	32
4	Proportion of both readings in range	80.0
5	Limits of agreement	0.54 to 1.62
6	Constant variance p.value	0.033
7	Constant ratio p.value	0.928
8	Ratio is 1 p.value	0.201

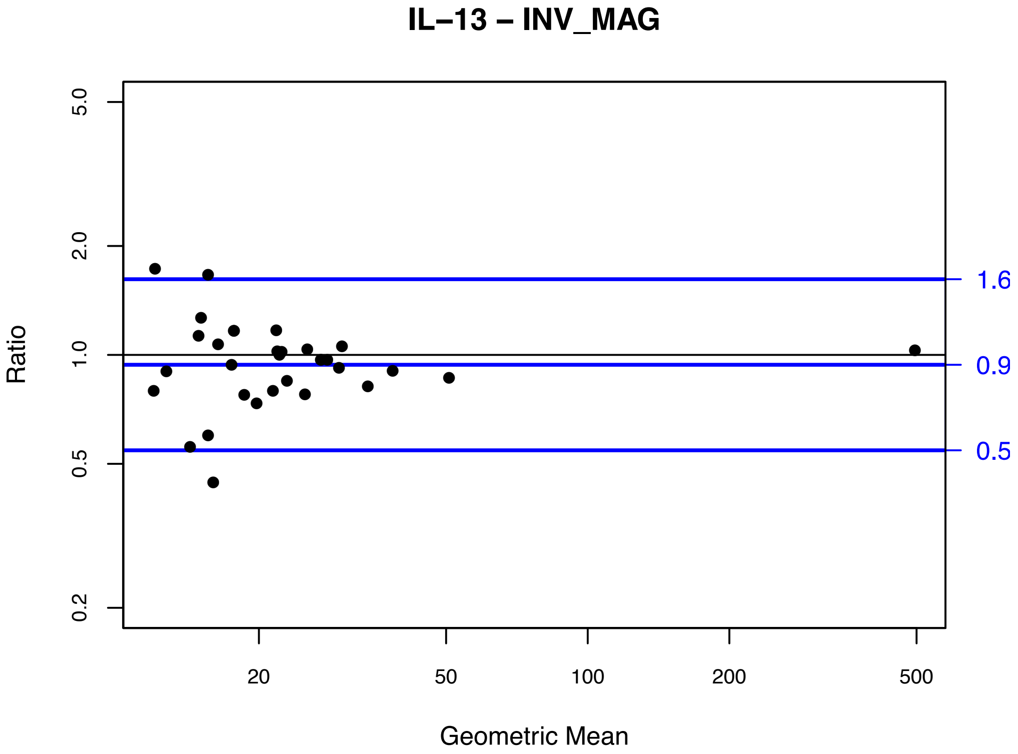

Supplement: Figure S10 — Mean difference dot plots of IL-13 for each kit tested. Disagreement plots show the difference between the duplicates against the geometric mean of both values of a sample tested with A) eBioscience® FlowCytomix™ (Bender), B) Bio-Rad® Bio-Plex Pro™ Human Cytokine Plex Assay (Bio-Rad) and C) Invitrogen™ Human Cytokine Magnetic 30-Plex Panel (INV-MAG). The middle line is the mean difference and the two extreme lines are the limits of agreement calculated by Bland-Altman test. (PDF) [file pone.0052587.s010.pdf]
