# Supplementary material for: Performance of Multiplex Commercial Kits to Quantify Cytokine and Chemokine Responses in Culture Supernatants from Plasmodium falciparum Stimulations
Source: PLoS One. 2013 Jan 2;8(1):e52587. doi: 10.1371/journal.pone.0052587 (PMC3534665; doi:10.1371/journal.pone.0052587)

Figure S12

A

	parameter	value
1	Cytokine	IL-17
2	Vendor	Bio-Rad
3	Samples included in this agreement	22
4	Proportion of both readings in range	59.5
5	Limits of agreement	0.74 to 1.54
6	Constant variance p.value	0.019
7	Constant ratio p.value	0.096
8	Ratio is 1 p.value	0.094

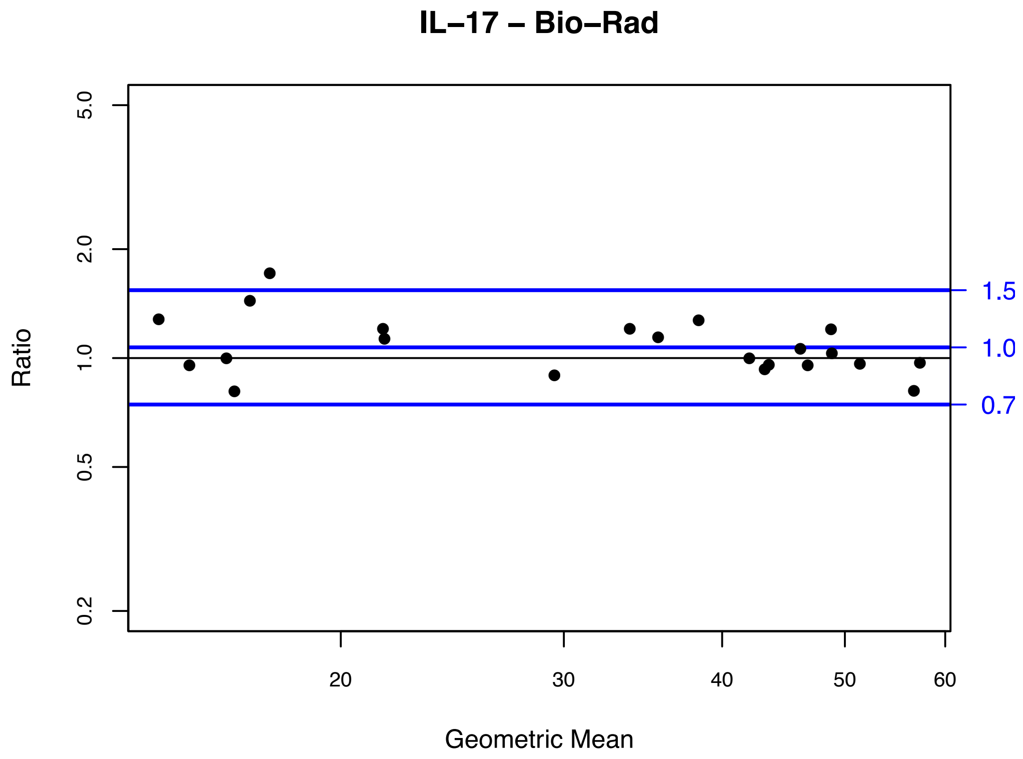

B

	parameter	value
1	Cytokine	IL-17
2	Vendor	INV_MAG
3	Samples included in this agreement	11
4	Proportion of both readings in range	27.5
5	Limits of agreement	0.31 to 2.06
6	Constant variance p.value	0.673
7	Constant ratio p.value	0.653
8	Ratio is 1 p.value	0.150

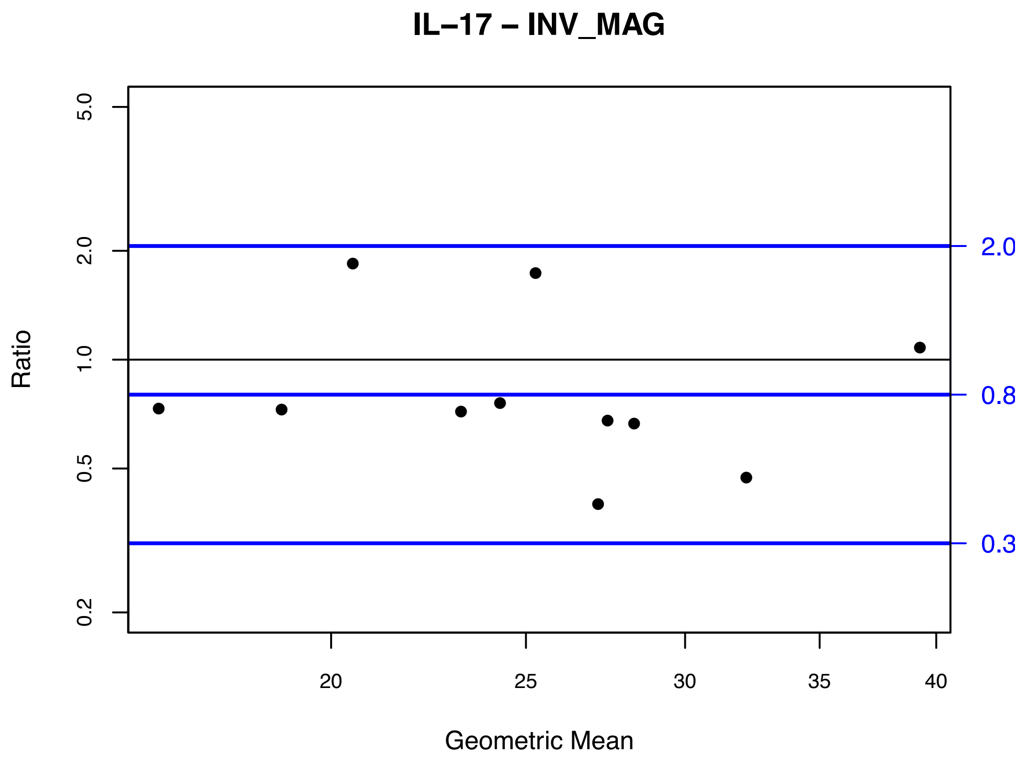

Supplement: Figure S12 — Mean difference dot plots of IL-17 for each kit tested. Disagreement plots show the difference between the duplicates against the geometric mean of both values of a sample tested with A) Bio-Rad® Bio-Plex Pro™ Human Cytokine Plex Assay (Bio-Rad) and B) Invitrogen™ Human Cytokine Magnetic 30-Plex Panel (INV-MAG). The middle line is the mean difference and the two extreme lines are the limits of agreement calculated by Bland-Altman test. (PDF) [file pone.0052587.s012.pdf]
