# Supplementary material for: Performance of Multiplex Commercial Kits to Quantify Cytokine and Chemokine Responses in Culture Supernatants from Plasmodium falciparum Stimulations
Source: PLoS One. 2013 Jan 2;8(1):e52587. doi: 10.1371/journal.pone.0052587 (PMC3534665; doi:10.1371/journal.pone.0052587)

Figure S13

A

	parameter	value
1	Cytokine	IL-1beta
2	Vendor	Bender
3	Samples included in this agreement	22
4	Proportion of both readings in range	59.5
5	Limits of agreement	0.64 to 1.74
6	Constant variance p.value	0.640
7	Constant ratio p.value	0.804
8	Ratio is 1 p.value	0.346

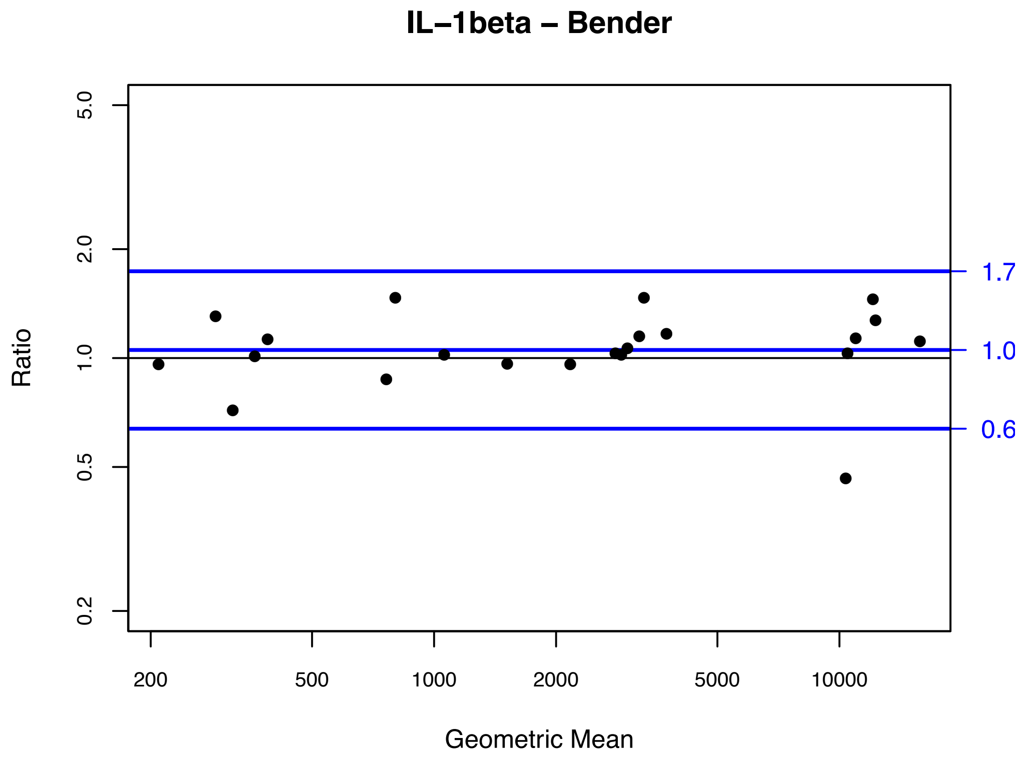

B

	parameter	value
1	Cytokine	IL-1beta
2	Vendor	Bio-Rad
3	Samples included in this agreement	30
4	Proportion of both readings in range	81.1
5	Limits of agreement	0.72 to 1.47
6	Constant variance p.value	0.612
7	Constant ratio p.value	0.091
8	Ratio is 1 p.value	0.377

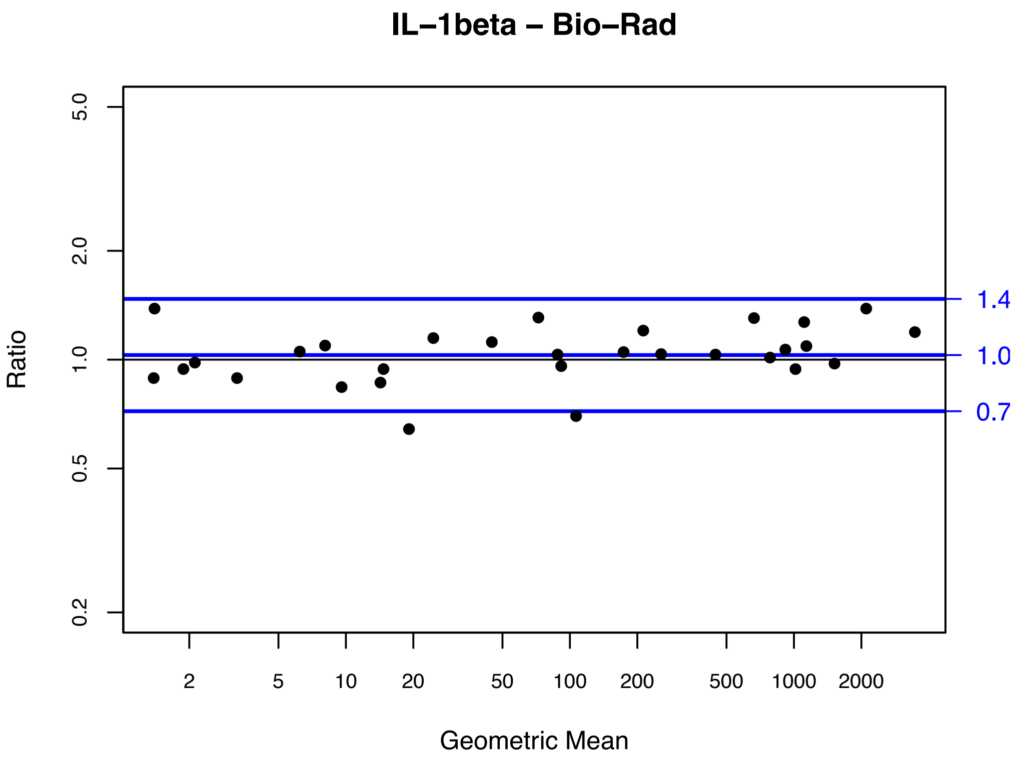

C

	parameter	value
1	Cytokine	IL-1beta
2	Vendor	Invitrogen
3	Samples included in this agreement	17
4	Proportion of both readings in range	45.9
5	Limits of agreement	0.46 to 2.71
6	Constant variance p.value	0.359
7	Constant ratio p.value	0.253
8	Ratio is 1 p.value	0.319

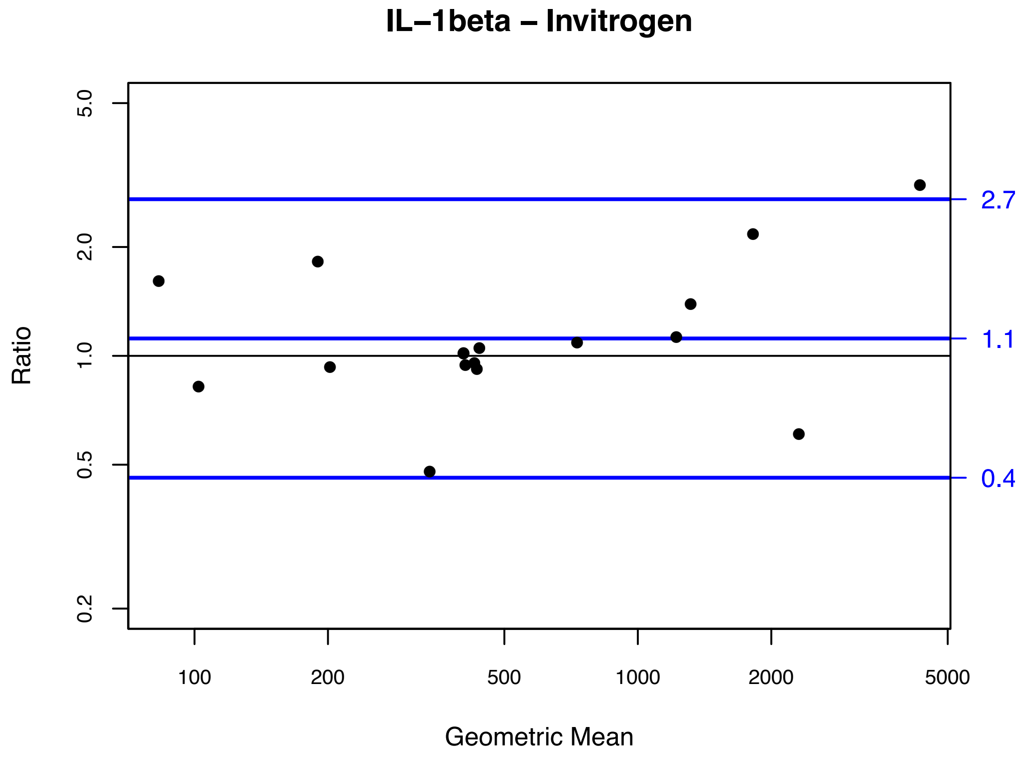

D

	parameter	value
1	Cytokine	IL-1beta
2	Vendor	INV_MAG
3	Samples included in this agreement	27
4	Proportion of both readings in range	67.5
5	Limits of agreement	0.88 to 1.23
6	Constant variance p.value	0.006
7	Constant ratio p.value	0.103
8	Ratio is 1 p.value	0.025

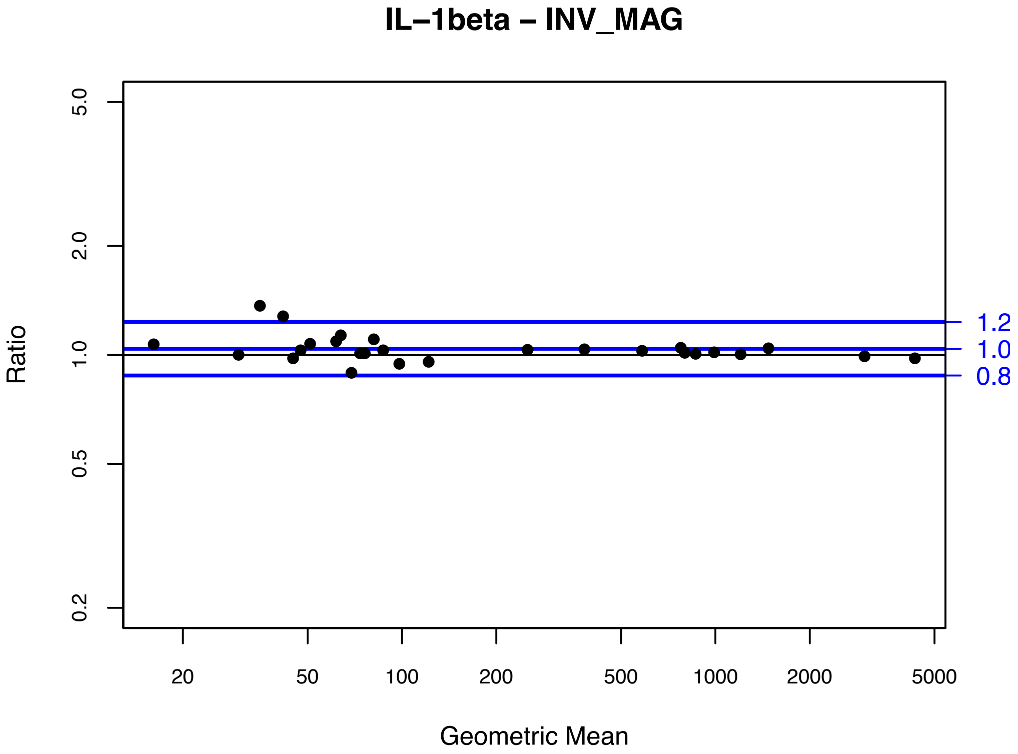

E

	parameter	value
1	Cytokine	IL-1beta
2	Vendor	Millipore
3	Samples included in this agreement	29
4	Proportion of both readings in range	78.4
5	Limits of agreement	0.77 to 1.27
6	Constant variance p.value	0.225
7	Constant ratio p.value	0.009
8	Ratio is 1 p.value	0.654

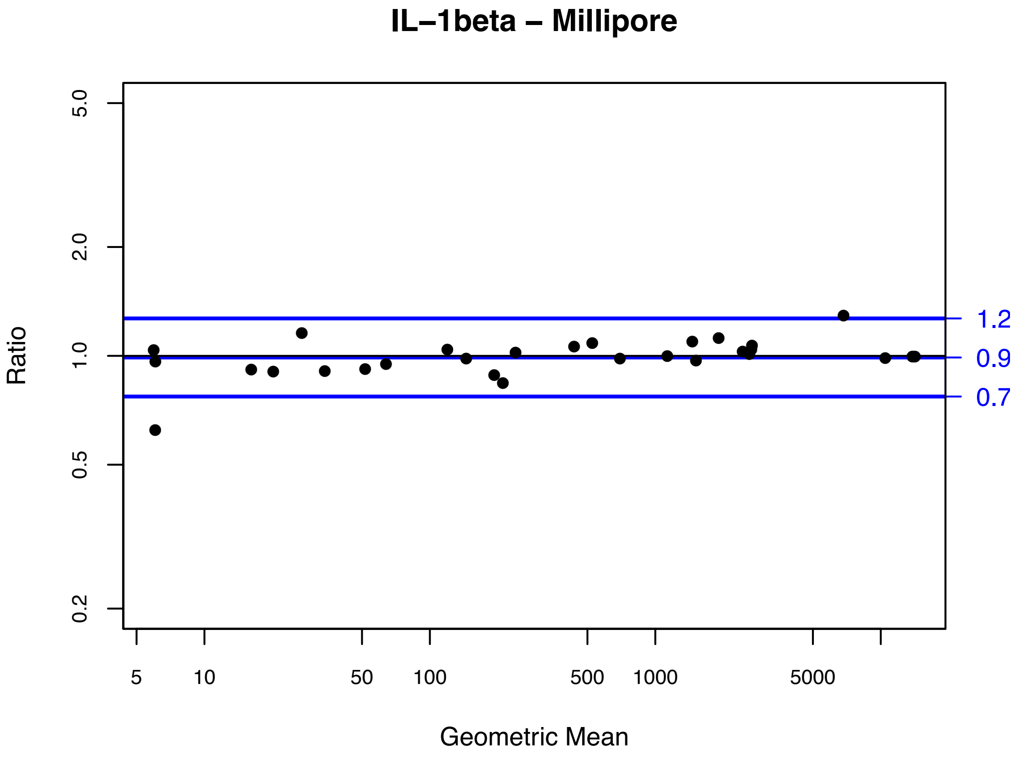

Supplement: Figure S13 — Mean difference dot plots of IL-1β for each kit tested. Disagreement plots show the difference between the duplicates against the geometric mean of both values of a sample tested with A) eBioscience® FlowCytomix™ (Bender), B) Bio-Rad® Bio-Plex Pro™ Human Cytokine Plex Assay (Bio-Rad), C) Human Cytokine 25-Plex panel from Invitrogen™ (non-magnetic beads), D) Invitrogen™ Human Cytokine Magnetic 30-Plex Panel (INV-MAG), and D) Millipore™ MILLIPLEX® MAP Plex Kit (Millipore). The middle line is the mean difference and the two extreme lines are the limits of agreement calculated by Bland-Altman test. (PDF) [file pone.0052587.s013.pdf]
