# Supplementary material for: Performance of Multiplex Commercial Kits to Quantify Cytokine and Chemokine Responses in Culture Supernatants from Plasmodium falciparum Stimulations
Source: PLoS One. 2013 Jan 2;8(1):e52587. doi: 10.1371/journal.pone.0052587 (PMC3534665; doi:10.1371/journal.pone.0052587)

Figure S15

A

	parameter	value
1	Cytokine	IL-2
2	Vendor	BD_CBA
3	Samples included in this agreement	7
4	Proportion of both readings in range	35.0
5	Limits of agreement	0.61 to 1.53
6	Constant variance p.value	0.556
7	Constant ratio p.value	0.787
8	Ratio is 1 p.value	0.715

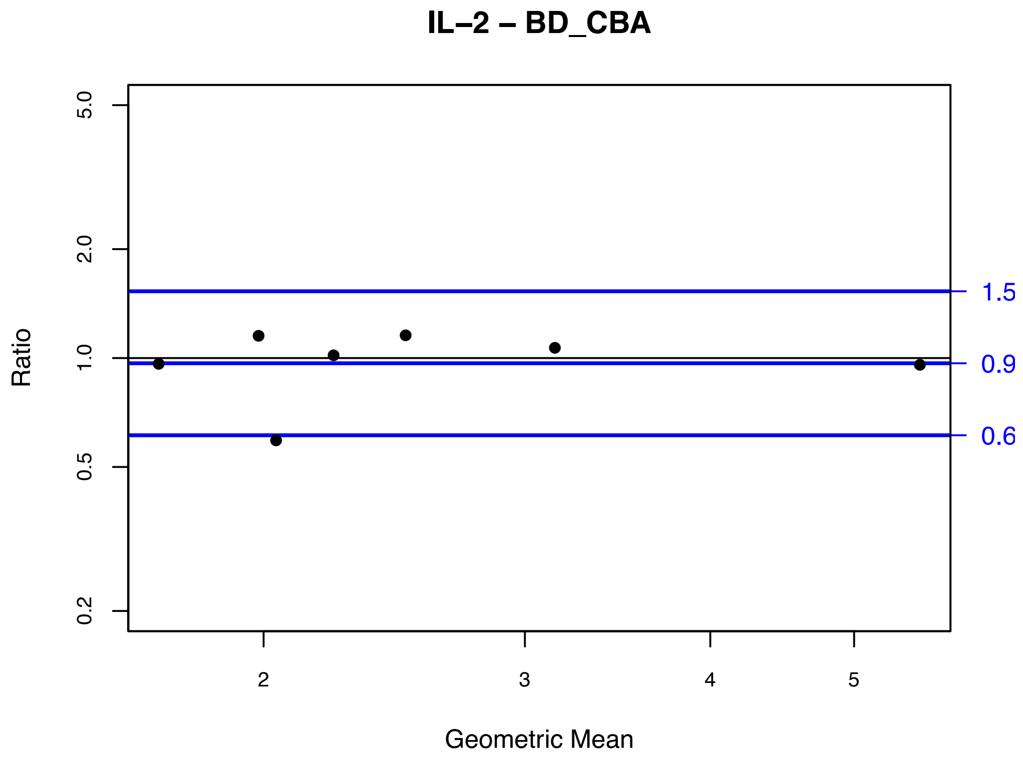

B

	parameter	value
1	Cytokine	IL-2
2	Vendor	Bio-Rad
3	Samples included in this agreement	17
4	Proportion of both readings in range	45.9
5	Limits of agreement	0.58 to 1.63
6	Constant variance p.value	0.948
7	Constant ratio p.value	0.666
8	Ratio is 1 p.value	0.662

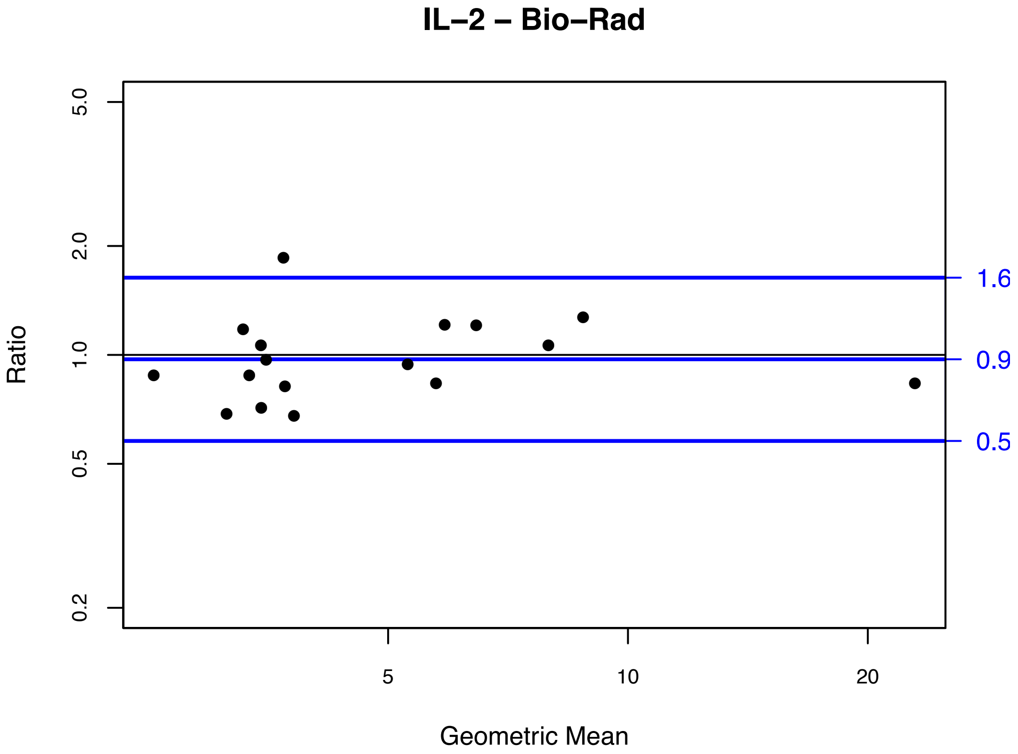

C

	parameter	value
1	Cytokine	IL-2
2	Vendor	INV_MAG
3	Samples included in this agreement	19
4	Proportion of both readings in range	47.5
5	Limits of agreement	0.67 to 1.94
6	Constant variance p.value	0.348
7	Constant ratio p.value	0.495
8	Ratio is 1 p.value	0.048

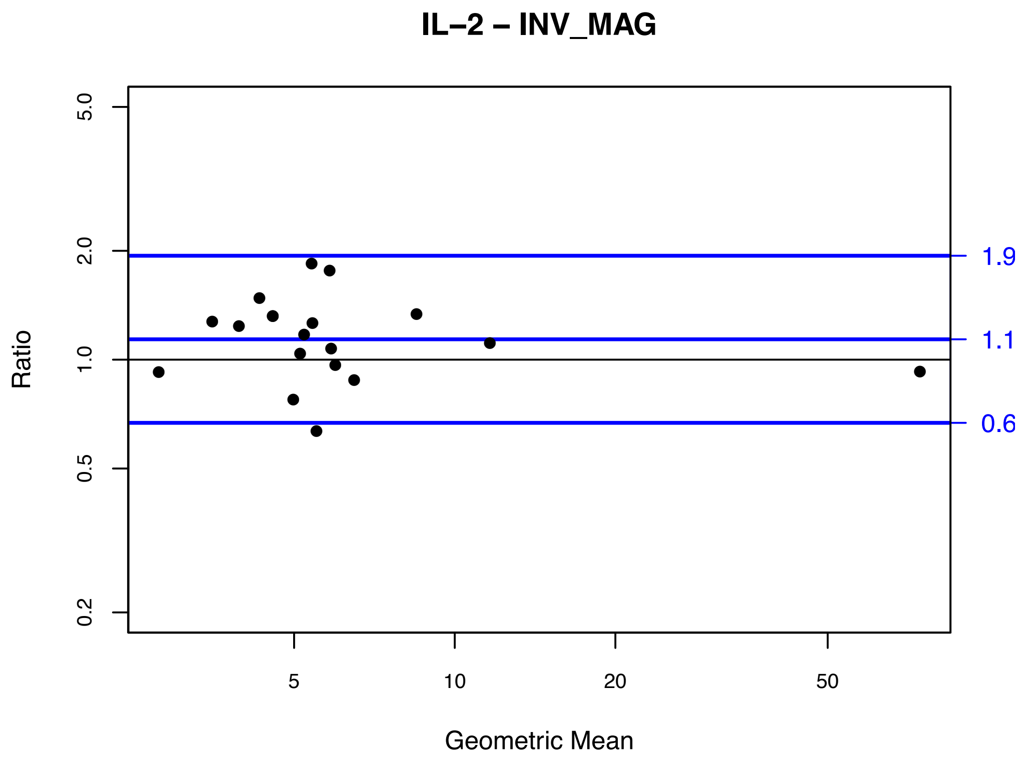

D

	parameter	value
1	Cytokine	IL-2
2	Vendor	Millipore
3	Samples included in this agreement	2
4	Proportion of both readings in range	5.4
5	Limits of agreement	0.83 to 1.18
6	Constant variance p.value	NaN
7	Constant ratio p.value	NaN
8	Ratio is 1 p.value	0.879

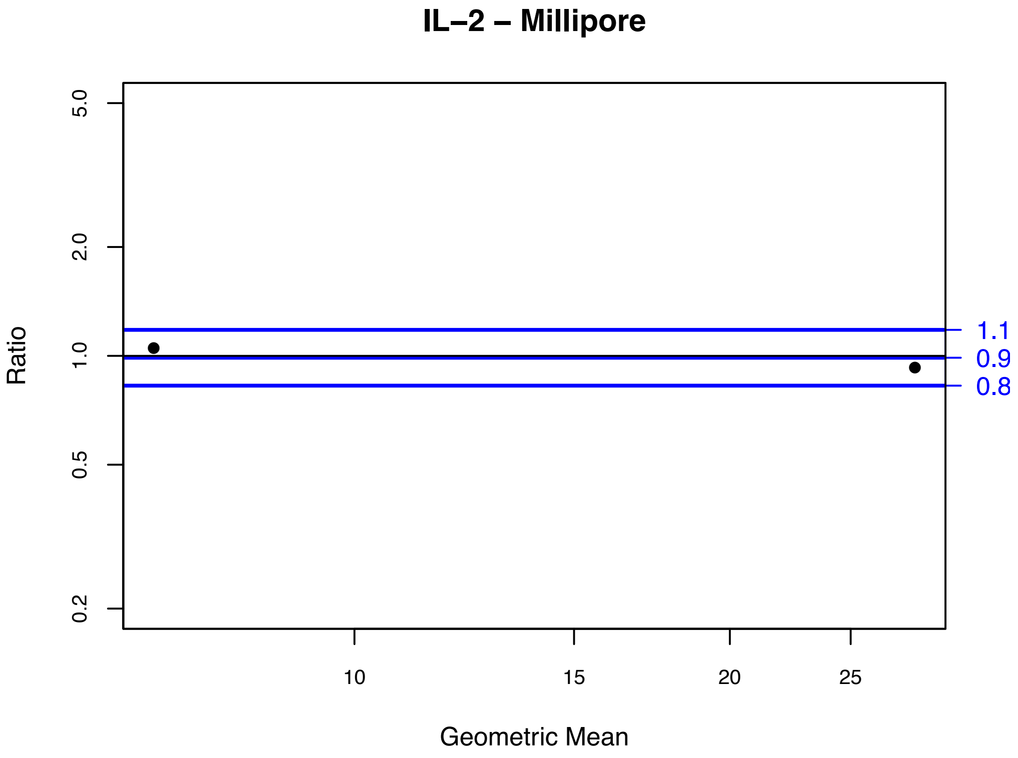

Supplement: Figure S15 — Mean difference dot plots of IL-2 for each kit tested. Disagreement plots show the difference between the duplicates against the geometric mean of both values of a sample tested with A) BD™ Cytometric Bead Array Human Enhanced Sensitivity kit (BD CBA), B) Bio-Rad® Bio-Plex Pro™ Human Cytokine Plex Assay (Bio-Rad), C) Invitrogen™ Human Cytokine Magnetic 30-Plex Panel (INV-MAG), and D) Millipore™ MILLIPLEX® MAP Plex Kit (Millipore). The middle line is the mean difference and the two extreme lines are the limits of agreement calculated by Bland-Altman test. (PDF) [file pone.0052587.s015.pdf]
