# Supplementary material for: Performance of Multiplex Commercial Kits to Quantify Cytokine and Chemokine Responses in Culture Supernatants from Plasmodium falciparum Stimulations
Source: PLoS One. 2013 Jan 2;8(1):e52587. doi: 10.1371/journal.pone.0052587 (PMC3534665; doi:10.1371/journal.pone.0052587)

Figure S17

A

	parameter	value
1	Cytokine	IL-4
2	Vendor	BD_CBA
3	Samples included in this agreement	3
4	Proportion of both readings in range	15.0
5	Limits of agreement	0.42 to 1.60
6	Constant variance p.value	0.679
7	Constant ratio p.value	0.144
8	Ratio is 1 p.value	0.413

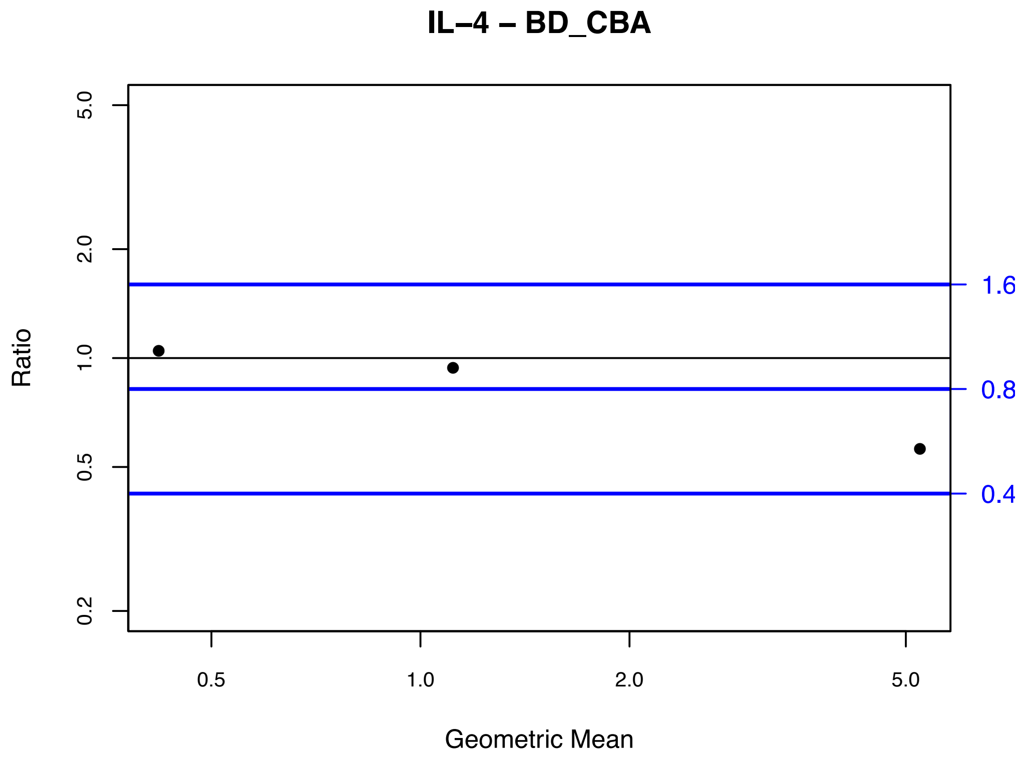

B

	parameter	value
1	Cytokine	IL-4
2	Vendor	Bio-Rad
3	Samples included in this agreement	33
4	Proportion of both readings in range	89.2
5	Limits of agreement	0.51 to 1.62
6	Constant variance p.value	0.001
7	Constant ratio p.value	0.540
8	Ratio is 1 p.value	0.059

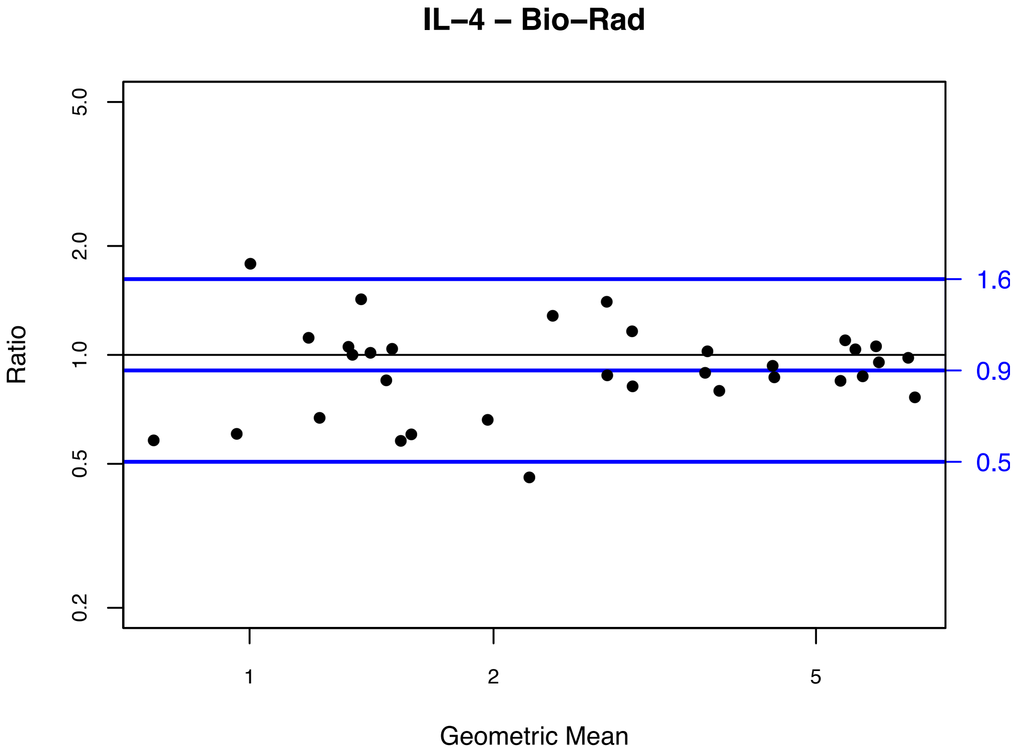

C

	parameter	value
1	Cytokine	IL-4
2	Vendor	INV_MAG
3	Samples included in this agreement	4
4	Proportion of both readings in range	10.0
5	Limits of agreement	0.27 to 1.47
6	Constant variance p.value	0.133
7	Constant ratio p.value	0.465
8	Ratio is 1 p.value	0.115

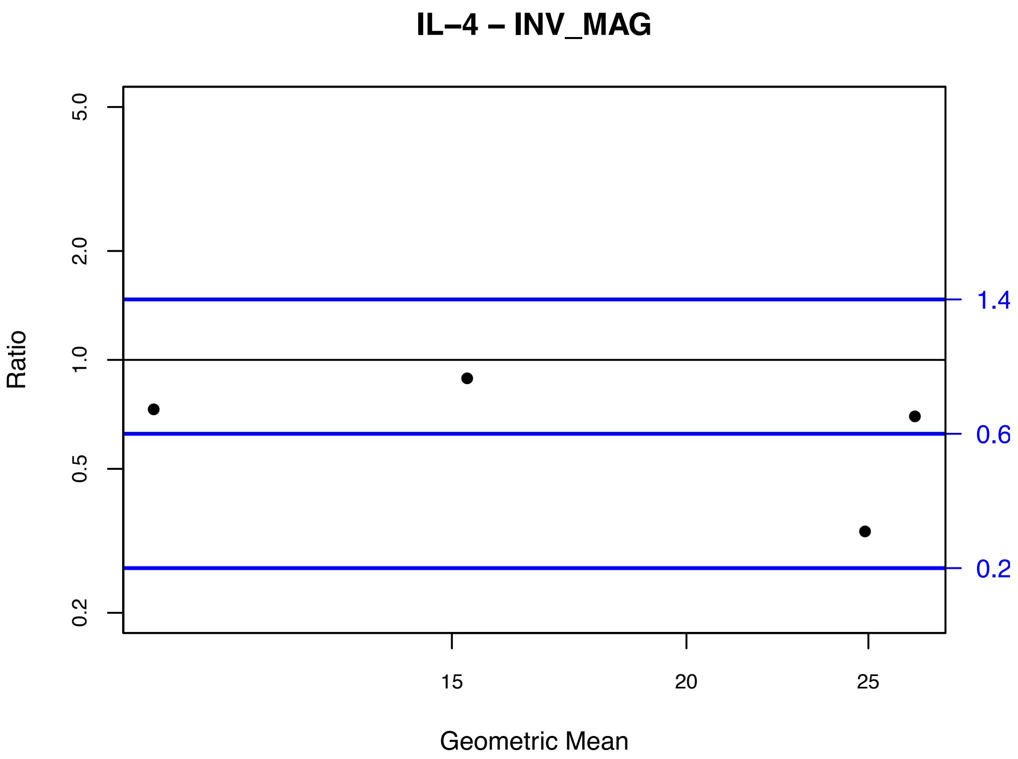

D

	parameter	value
1	Cytokine	IL-4
2	Vendor	Millipore
3	Samples included in this agreement	18
4	Proportion of both readings in range	48.6
5	Limits of agreement	0.61 to 1.61
6	Constant variance p.value	0.592
7	Constant ratio p.value	0.620
8	Ratio is 1 p.value	0.867

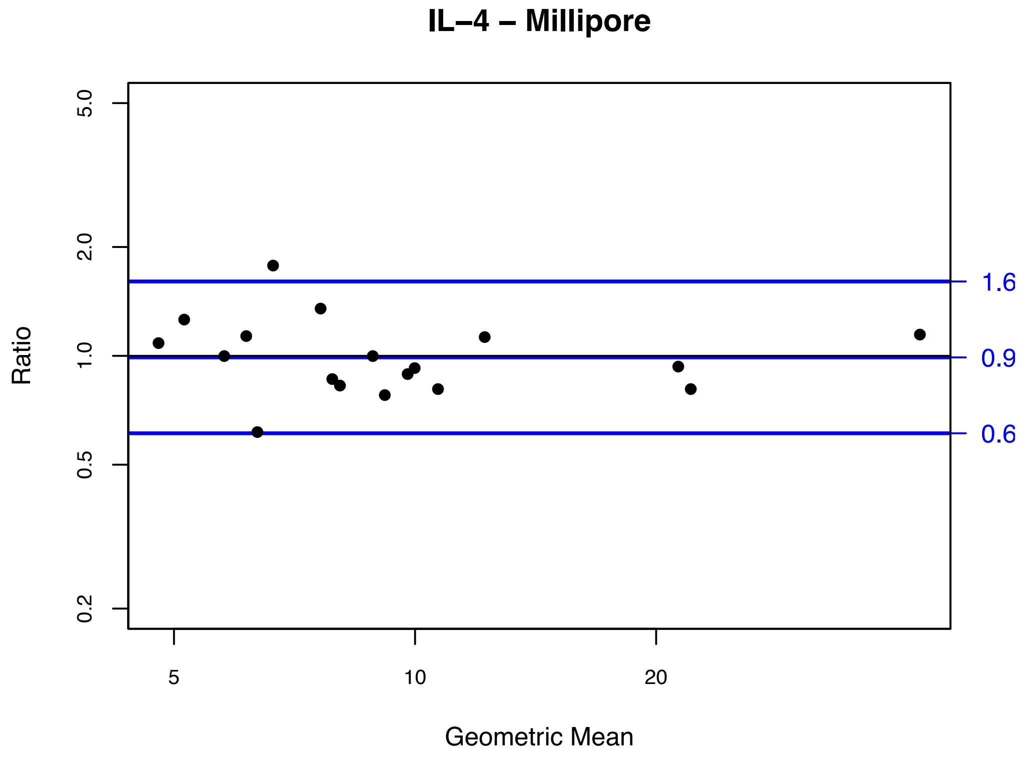

Supplement: Figure S17 — Mean difference dot plots of IL-4 for each kit tested. Disagreement plots show the difference between the duplicates against the geometric mean of both values of a sample tested with A) BD™ Cytometric Bead Array Human Enhanced Sensitivity kit (BD CBA), B) Bio-Rad® Bio-Plex Pro™ Human Cytokine Plex Assay (Bio-Rad), C) Invitrogen™ Human Cytokine Magnetic 30-Plex Panel (INV-MAG), and D) Millipore™ MILLIPLEX® MAP Plex Kit (Millipore). The middle line is the mean difference and the two extreme lines are the limits of agreement calculated by Bland-Altman test. (PDF) [file pone.0052587.s017.pdf]
