# Supplementary material for: Performance of Multiplex Commercial Kits to Quantify Cytokine and Chemokine Responses in Culture Supernatants from Plasmodium falciparum Stimulations
Source: PLoS One. 2013 Jan 2;8(1):e52587. doi: 10.1371/journal.pone.0052587 (PMC3534665; doi:10.1371/journal.pone.0052587)

Figure S18

A

	parameter	value
1	Cytokine	IL-5
2	Vendor	Bio-Rad
3	Samples included in this agreement	12
4	Proportion of both readings in range	32.4
5	Limits of agreement	0.52 to 2.78
6	Constant variance p.value	0.426
7	Constant ratio p.value	0.466
8	Ratio is 1 p.value	0.155

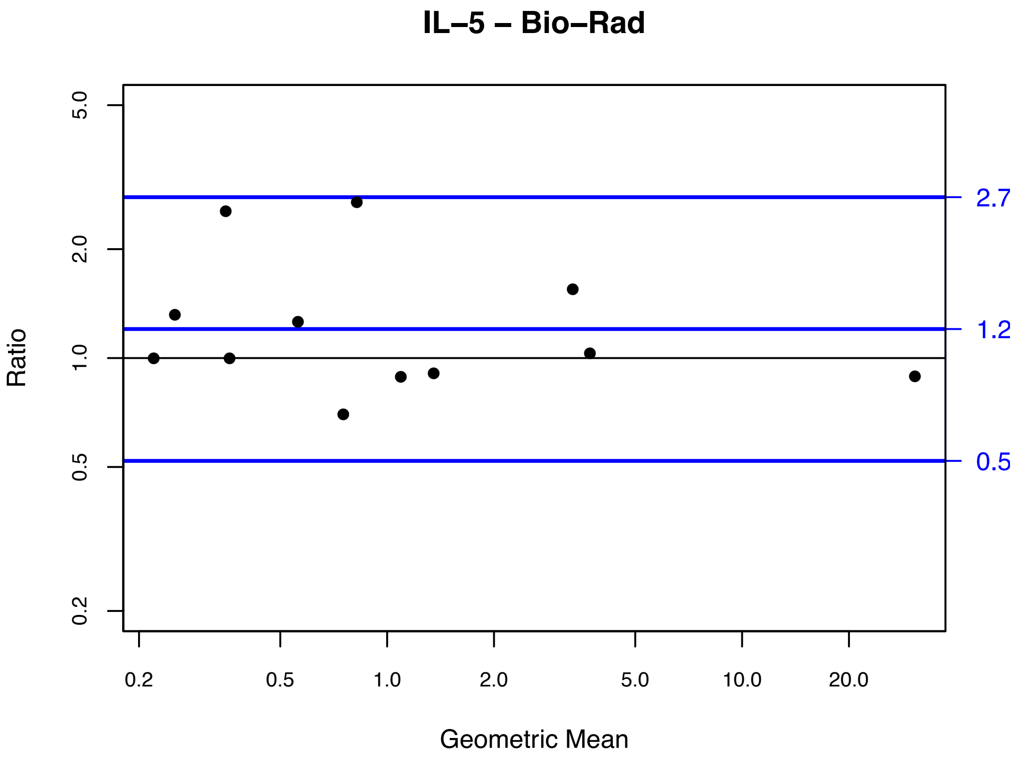

B

	parameter	value
1	Cytokine	IL-5
2	Vendor	Millipore
3	Samples included in this agreement	2
4	Proportion of both readings in range	5.4
5	Limits of agreement	0.94 to 0.95
6	Constant variance p.value	NaN
7	Constant ratio p.value	NaN
8	Ratio is 1 p.value	0.028

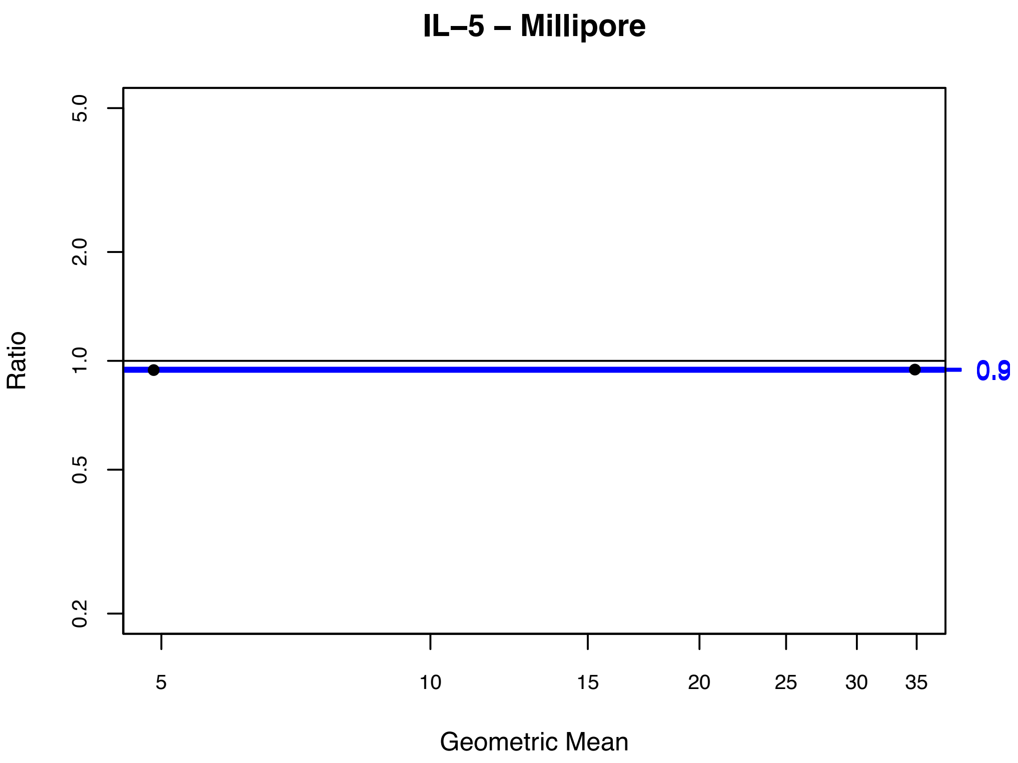

Supplement: Figure S18 — Mean difference dot plots of IL-5 for each kit tested. Disagreement plots show the difference between the duplicates against the geometric mean of both values of a sample tested with A) Bio-Rad® Bio-Plex Pro™ Human Cytokine Plex Assay (Bio-Rad), and B) Millipore™ MILLIPLEX® MAP Plex Kit (Millipore). The middle line is the mean difference and the two extreme lines are the limits of agreement calculated by Bland-Altman test. (PDF) [file pone.0052587.s018.pdf]
