# Supplementary material for: Performance of Multiplex Commercial Kits to Quantify Cytokine and Chemokine Responses in Culture Supernatants from Plasmodium falciparum Stimulations
Source: PLoS One. 2013 Jan 2;8(1):e52587. doi: 10.1371/journal.pone.0052587 (PMC3534665; doi:10.1371/journal.pone.0052587)

Figure S19

A

	parameter	value
1	Cytokine	IL-6
2	Vendor	Bender
3	Samples included in this agreement	29
4	Proportion of both readings in range	78.4
5	Limits of agreement	0.68 to 1.52
6	Constant variance p.value	0.590
7	Constant ratio p.value	0.629
8	Ratio is 1 p.value	0.676

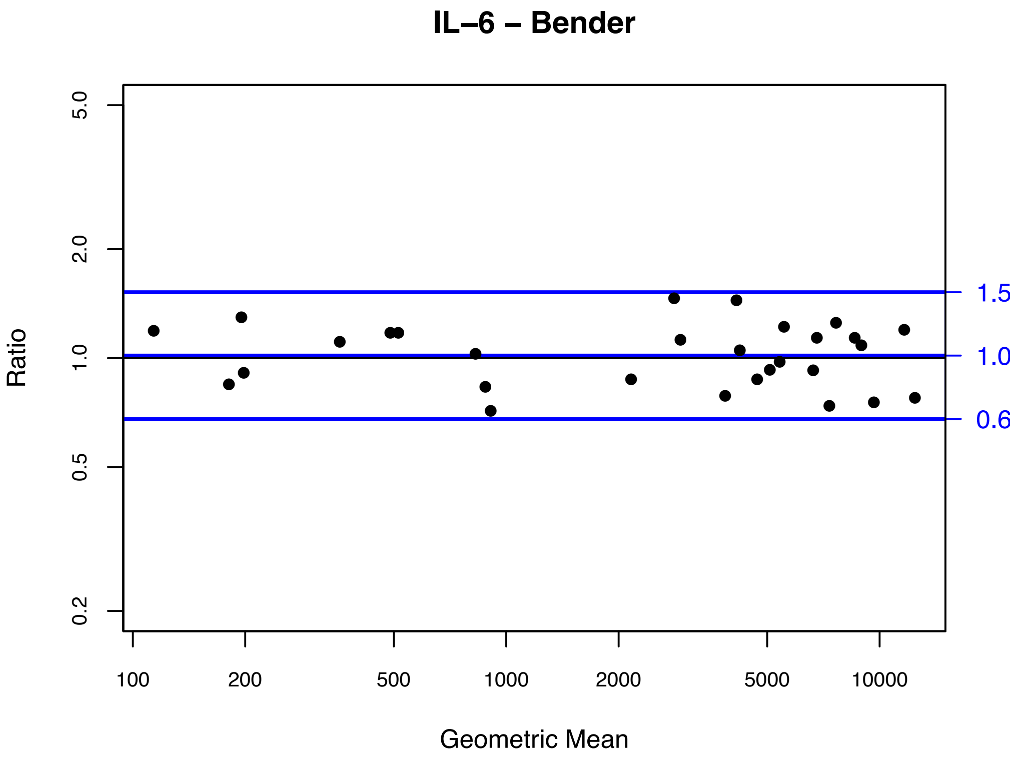

B

	parameter	value
1	Cytokine	IL-6
2	Vendor	Bio-Rad
3	Samples included in this agreement	21
4	Proportion of both readings in range	56.8
5	Limits of agreement	0.72 to 1.40
6	Constant variance p.value	0.707
7	Constant ratio p.value	0.072
8	Ratio is 1 p.value	0.980

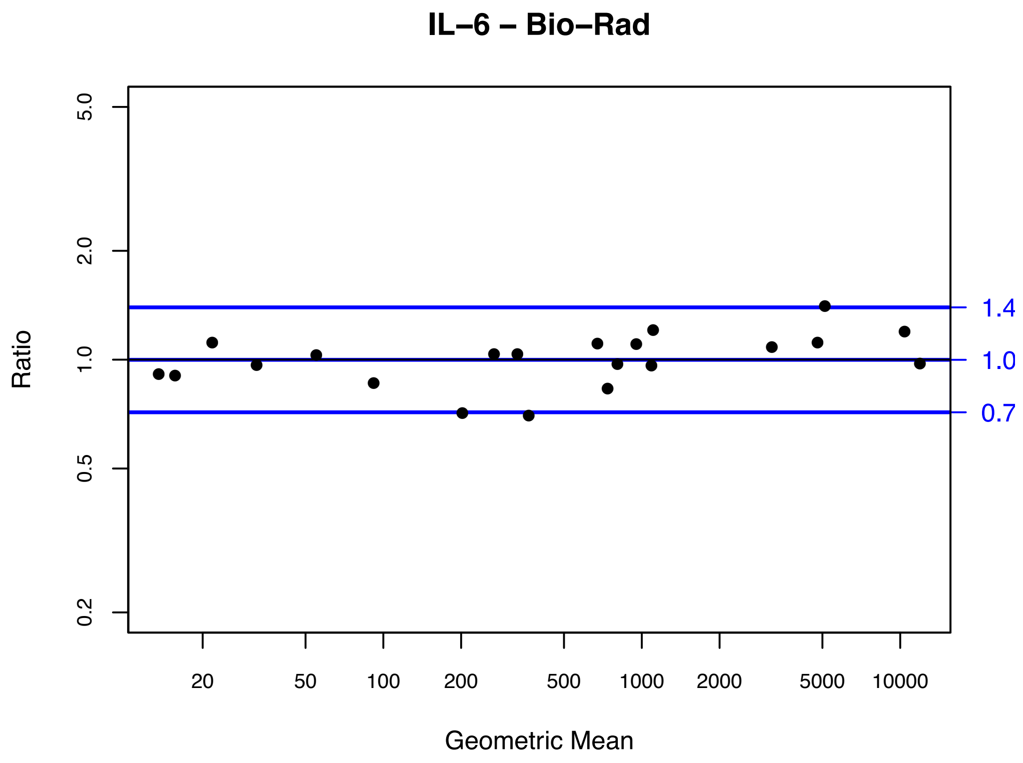

C

	parameter	value
1	Cytokine	IL-6
2	Vendor	Invitrogen
3	Samples included in this agreement	21
4	Proportion of both readings in range	56.8
5	Limits of agreement	0.51 to 2.43
6	Constant variance p.value	0.957
7	Constant ratio p.value	0.365
8	Ratio is 1 p.value	0.216

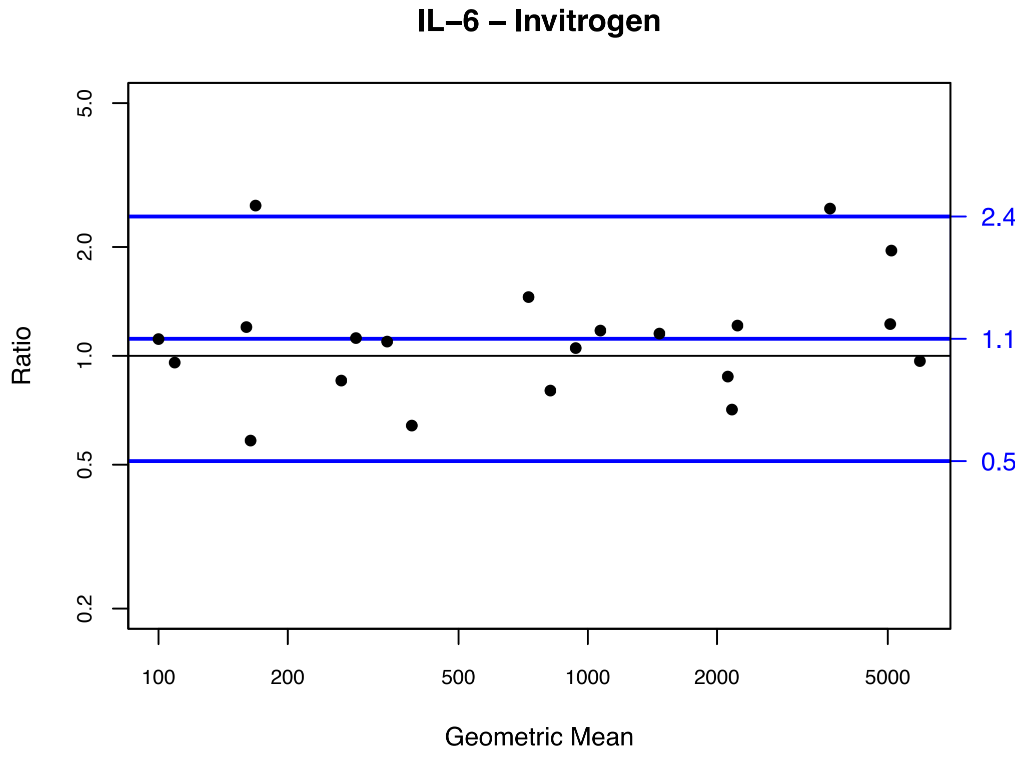

D

	parameter	value
1	Cytokine	IL-6
2	Vendor	INV_MAG
3	Samples included in this agreement	30
4	Proportion of both readings in range	75.0
5	Limits of agreement	0.89 to 1.18
6	Constant variance p.value	0.007
7	Constant ratio p.value	0.015
8	Ratio is 1 p.value	0.094

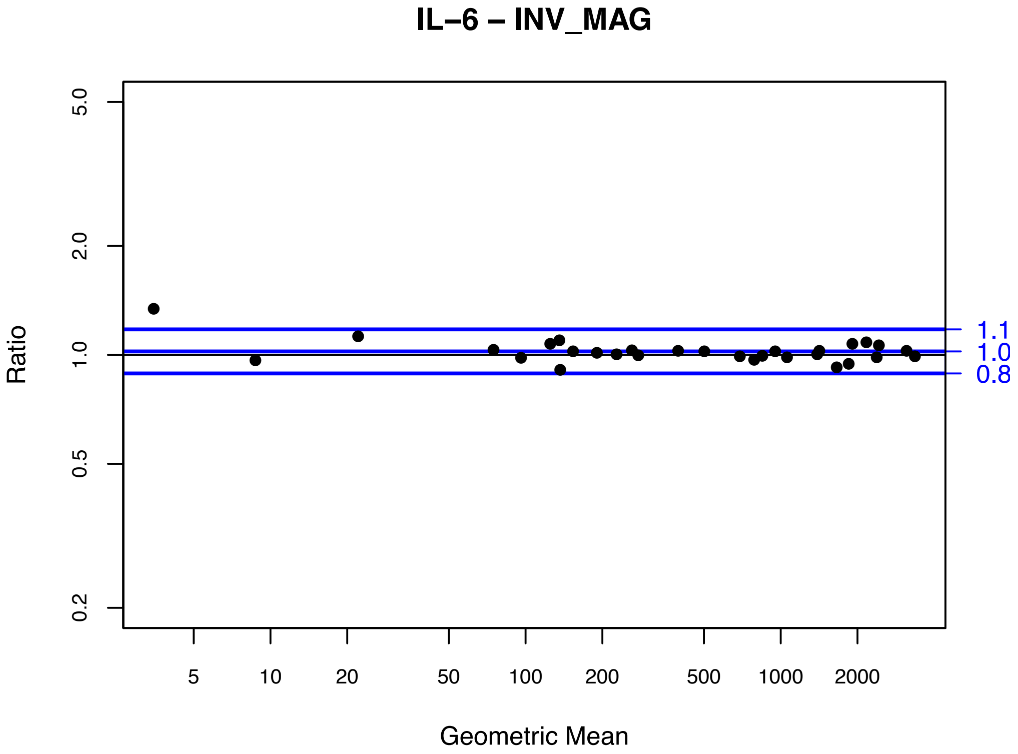

E

	parameter	value
1	Cytokine	IL-6
2	Vendor	Millipore
3	Samples included in this agreement	25
4	Proportion of both readings in range	67.6
5	Limits of agreement	0.77 to 1.25
6	Constant variance p.value	0.004
7	Constant ratio p.value	0.073
8	Ratio is 1 p.value	0.411

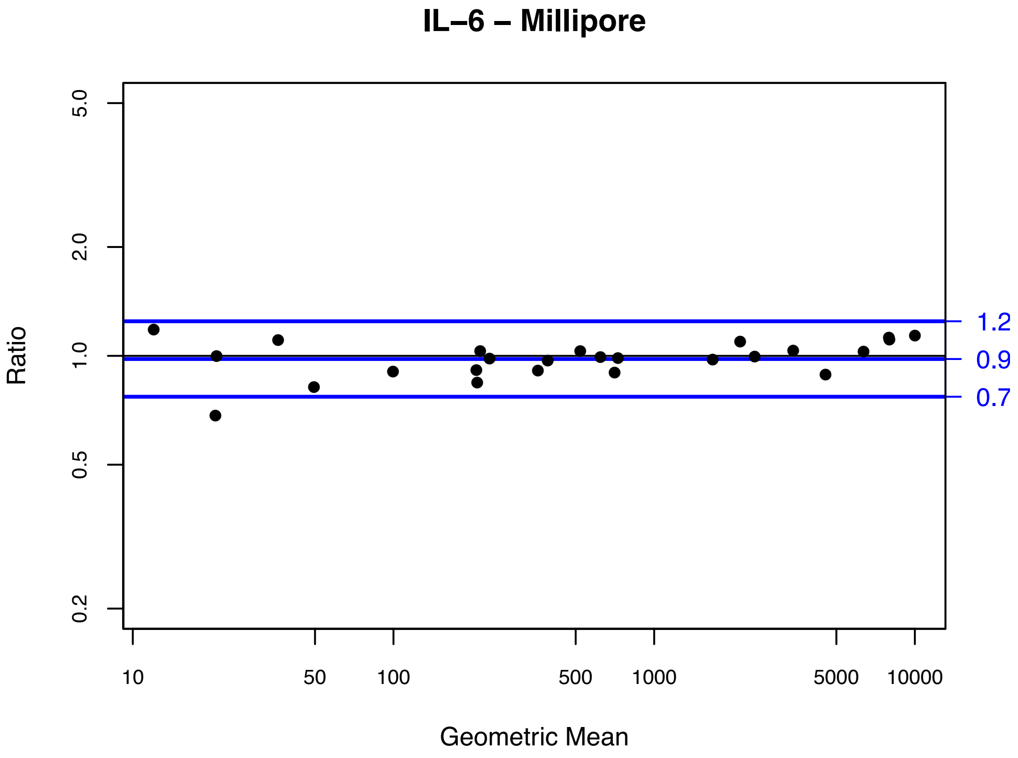

Supplement: Figure S19 — Mean difference dot plots of IL-6 for each kit tested. Disagreement plots show the difference between the duplicates against the geometric mean of both values of a sample tested with A) eBioscience® FlowCytomix™ (Bender), B) Bio-Rad® Bio-Plex Pro™ Human Cytokine Plex Assay (Bio-Rad), C) Human Cytokine 25-Plex panel from Invitrogen™ (non-magnetic beads), D) Invitrogen™ Human Cytokine Magnetic 30-Plex Panel (INV-MAG), and E) Millipore™ MILLIPLEX® MAP Plex Kit (Millipore). The middle line is the mean difference and the two extreme lines are the limits of agreement calculated by Bland-Altman test. (PDF) [file pone.0052587.s019.pdf]
