# Supplementary material for: Performance of Multiplex Commercial Kits to Quantify Cytokine and Chemokine Responses in Culture Supernatants from Plasmodium falciparum Stimulations
Source: PLoS One. 2013 Jan 2;8(1):e52587. doi: 10.1371/journal.pone.0052587 (PMC3534665; doi:10.1371/journal.pone.0052587)

Figure S20

A

	parameter	value
1	Cytokine	IL-7
2	Vendor	Bio-Rad
3	Samples included in this agreement	36
4	Proportion of both readings in range	97.3
5	Limits of agreement	0.38 to 2.91
6	Constant variance p.value	0.026
7	Constant ratio p.value	0.684
8	Ratio is 1 p.value	0.510

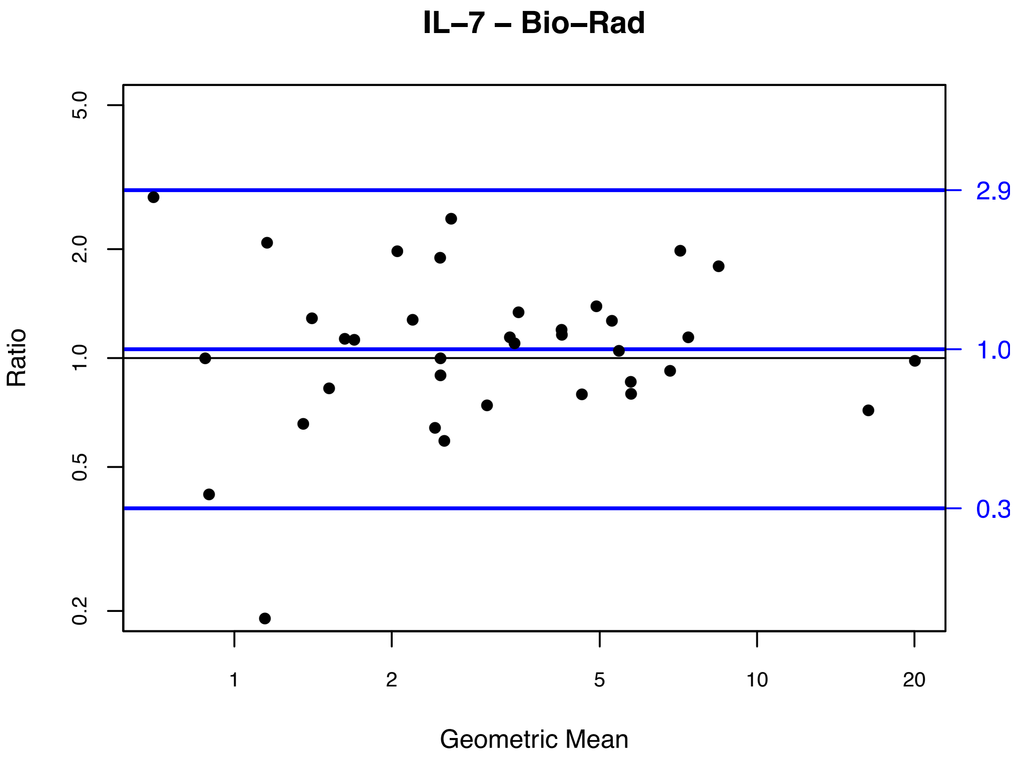

B

	parameter	value
1	Cytokine	IL-7
2	Vendor	INV_MAG
3	Samples included in this agreement	7
4	Proportion of both readings in range	17.5
5	Limits of agreement	1.11 to 1.36
6	Constant variance p.value	0.314
7	Constant ratio p.value	0.919
8	Ratio is 1 p.value	0.000

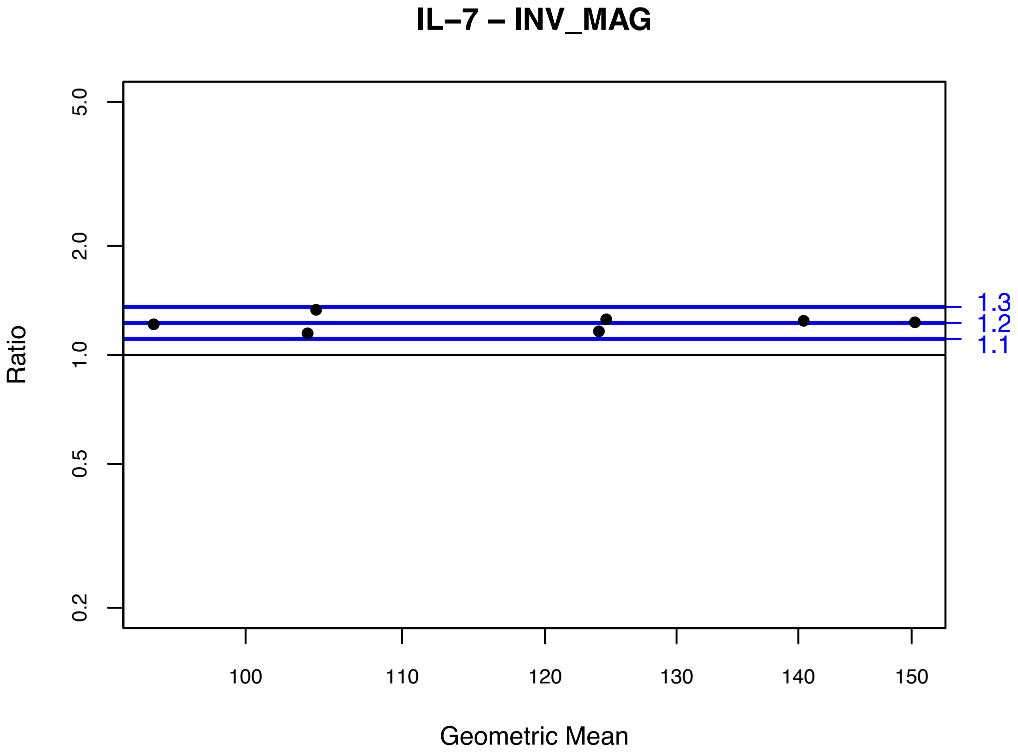

Supplement: Figure S20 — Mean difference dot plots of IL-7 for each kit tested. Disagreement plots show the difference between the duplicates against the geometric mean of both values of a sample tested with A) Bio-Rad® Bio-Plex Pro™ Human Cytokine Plex Assay (Bio-Rad) and B) Invitrogen™ Human Cytokine Magnetic 30-Plex Panel (INV-MAG). The middle line is the mean difference and the two extreme lines are the limits of agreement calculated by Bland-Altman test. (PDF) [file pone.0052587.s020.pdf]
