# Supplementary material for: Performance of Multiplex Commercial Kits to Quantify Cytokine and Chemokine Responses in Culture Supernatants from Plasmodium falciparum Stimulations
Source: PLoS One. 2013 Jan 2;8(1):e52587. doi: 10.1371/journal.pone.0052587 (PMC3534665; doi:10.1371/journal.pone.0052587)

Figure S21

A

	parameter	value
1	Cytokine	IL-8
2	Vendor	Bender
3	Samples included in this agreement	30
4	Proportion of both readings in range	81.1
5	Limits of agreement	0.58 to 1.57
6	Constant variance p.value	0.003
7	Constant ratio p.value	0.126
8	Ratio is 1 p.value	0.310

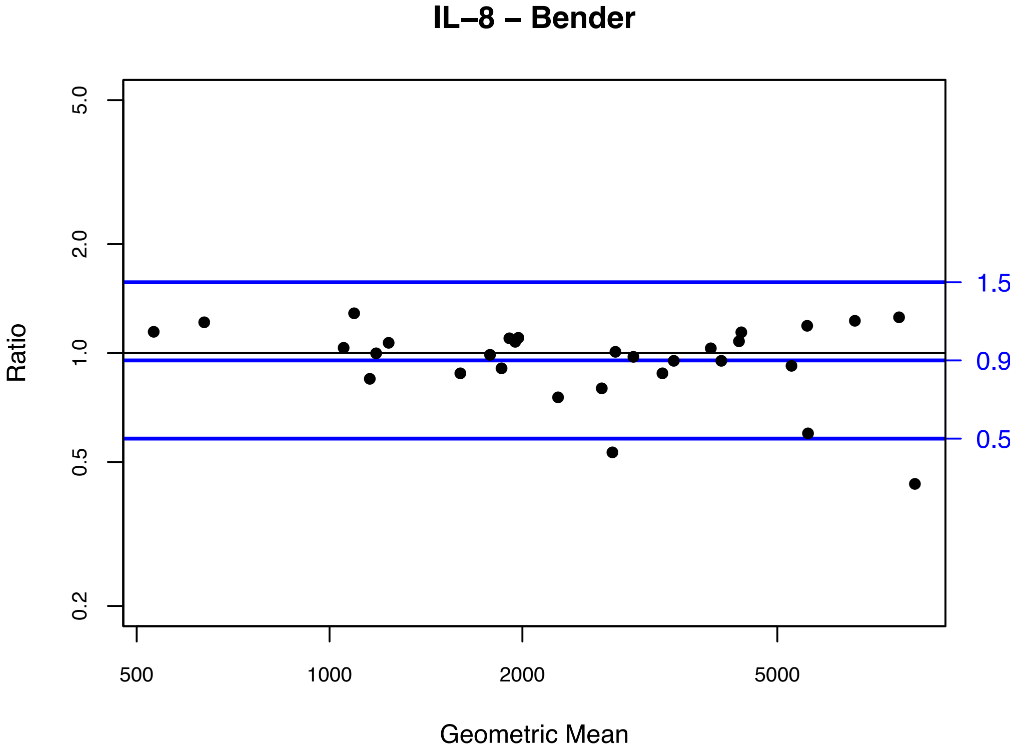

B

	parameter	value
1	Cytokine	IL-8
2	Vendor	Bio-Rad
3	Samples included in this agreement	4
4	Proportion of both readings in range	10.8
5	Limits of agreement	0.79 to 1.15
6	Constant variance p.value	0.534
7	Constant ratio p.value	0.715
8	Ratio is 1 p.value	0.373

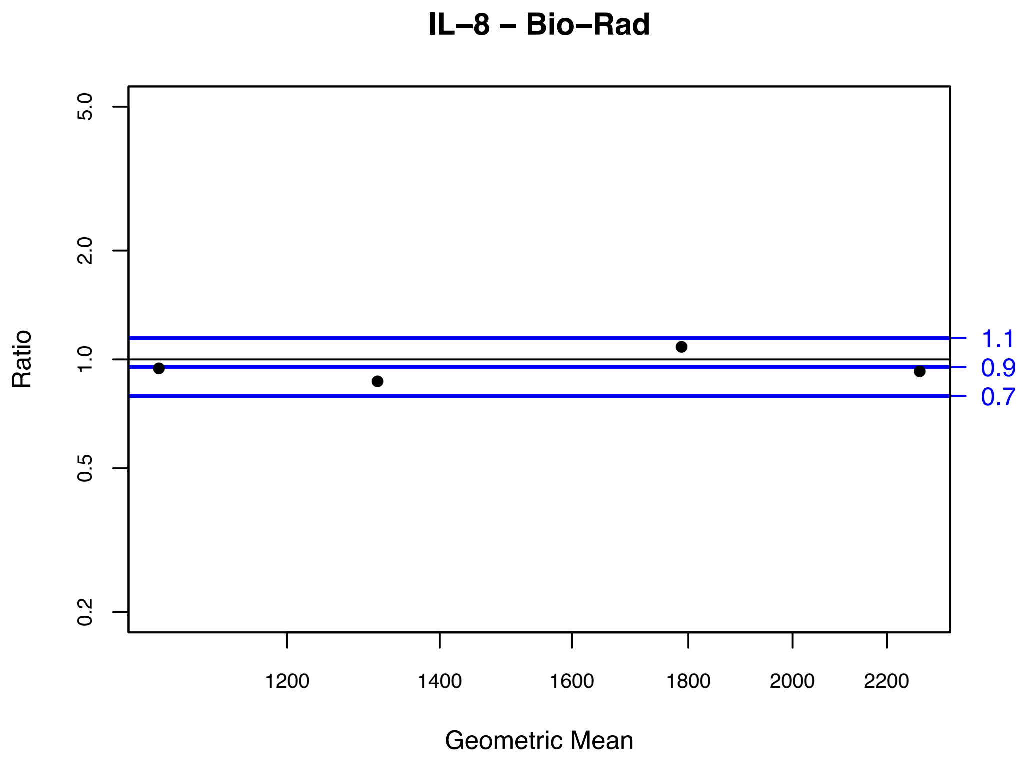

C

	parameter	value
1	Cytokine	IL-8
2	Vendor	Invitrogen
3	Samples included in this agreement	15
4	Proportion of both readings in range	40.5
5	Limits of agreement	0.47 to 2.40
6	Constant variance p.value	0.752
7	Constant ratio p.value	0.008
8	Ratio is 1 p.value	0.556

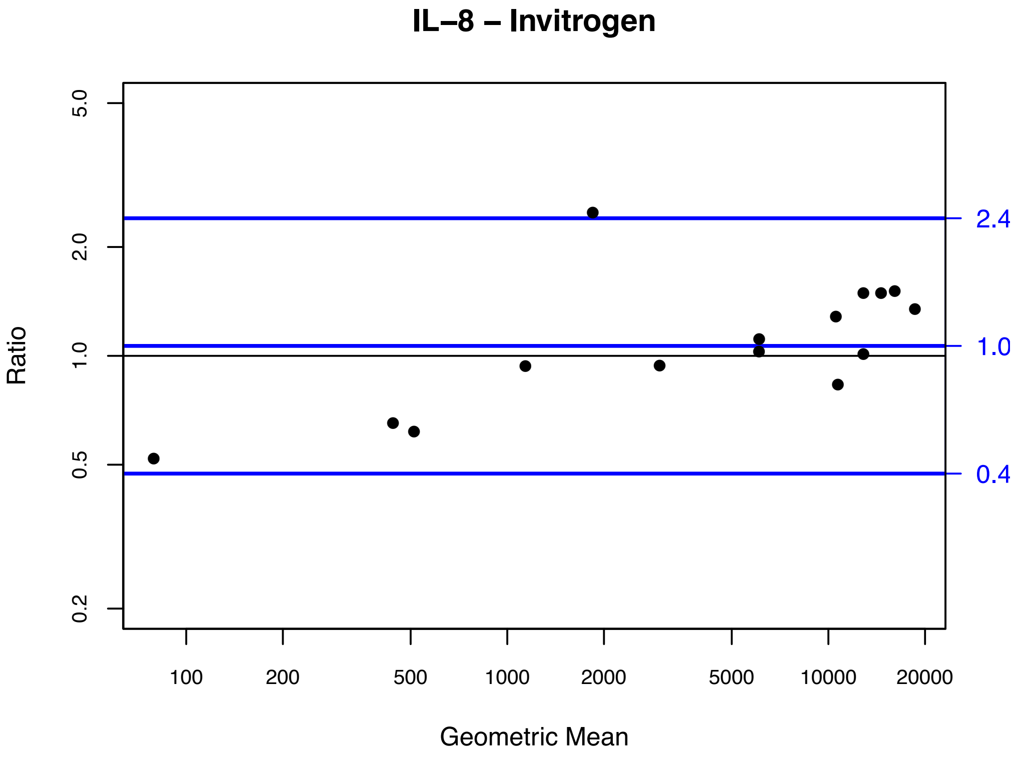

D

	parameter	value
1	Cytokine	IL-8
2	Vendor	INV_MAG
3	Samples included in this agreement	9
4	Proportion of both readings in range	22.5
5	Limits of agreement	0.93 to 1.17
6	Constant variance p.value	0.055
7	Constant ratio p.value	0.112
8	Ratio is 1 p.value	0.051

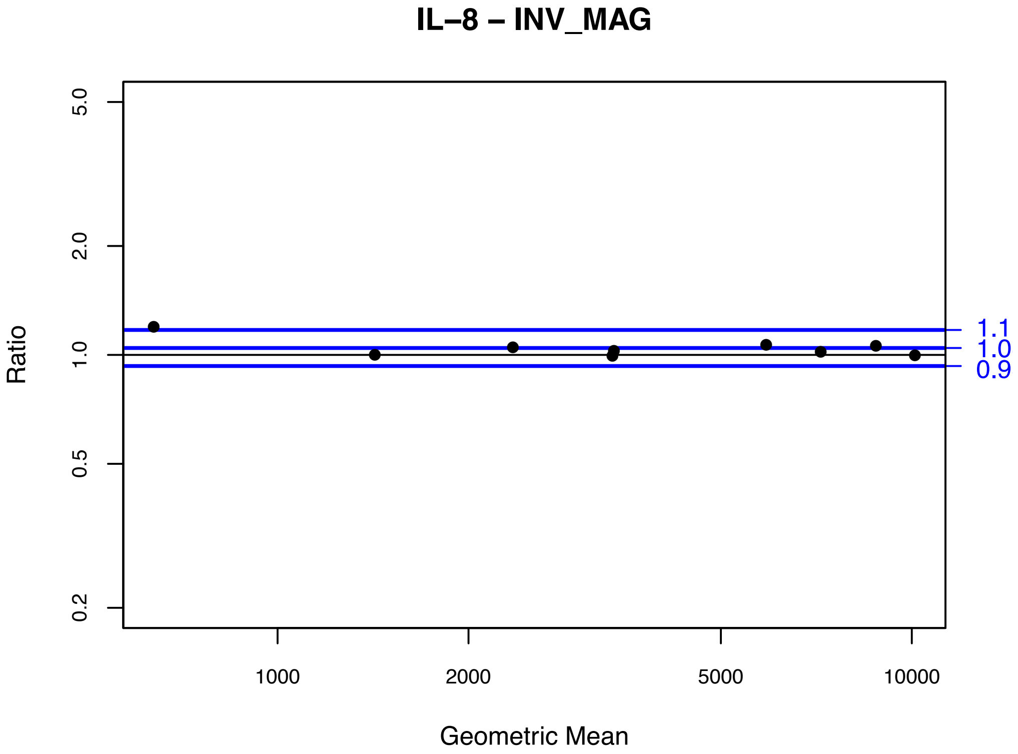

E

	parameter	value
1	Cytokine	IL-8
2	Vendor	Millipore
3	Samples included in this agreement	13
4	Proportion of both readings in range	35.1
5	Limits of agreement	0.81 to 1.14
6	Constant variance p.value	0.965
7	Constant ratio p.value	0.924
8	Ratio is 1 p.value	0.119

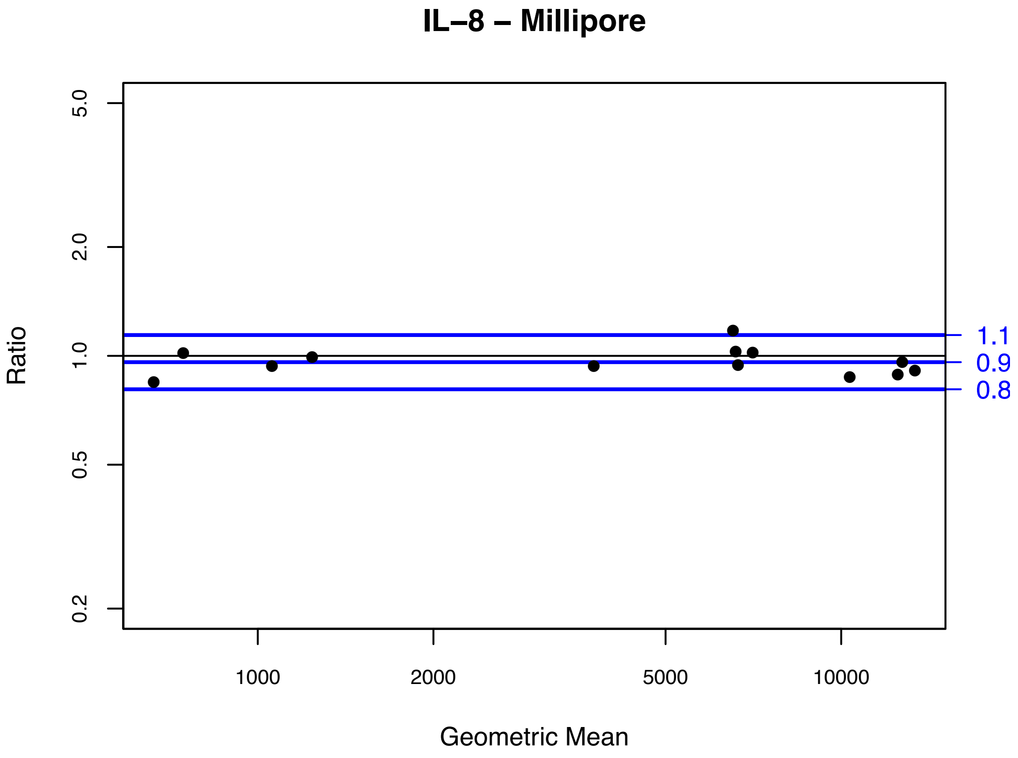

Supplement: Figure S21 — Mean difference dot plots of IL-8 for each kit tested. Disagreement plots show the difference between the duplicates against the geometric mean of both values of a sample tested with A) eBioscience® FlowCytomix™ (Bender), B) Bio-Rad® Bio-Plex Pro™ Human Cytokine Plex Assay (Bio-Rad), C) Human Cytokine 25-Plex panel from Invitrogen™ (non-magnetic beads), D) Invitrogen™ Human Cytokine Magnetic 30-Plex Panel (INV-MAG), and E) Millipore™ MILLIPLEX® MAP Plex Kit (Millipore). The middle line is the mean difference and the two extreme lines are the limits of agreement calculated by Bland-Altman test. (PDF) [file pone.0052587.s021.pdf]
