# Supplementary material for: Performance of Multiplex Commercial Kits to Quantify Cytokine and Chemokine Responses in Culture Supernatants from Plasmodium falciparum Stimulations
Source: PLoS One. 2013 Jan 2;8(1):e52587. doi: 10.1371/journal.pone.0052587 (PMC3534665; doi:10.1371/journal.pone.0052587)

Figure S22

A

	parameter	value
1	Cytokine	IP-10
2	Vendor	Invitrogen
3	Samples included in this agreement	7
4	Proportion of both readings in range	18.9
5	Limits of agreement	0.45 to 2.52
6	Constant variance p.value	0.628
7	Constant ratio p.value	0.193
8	Ratio is 1 p.value	0.724

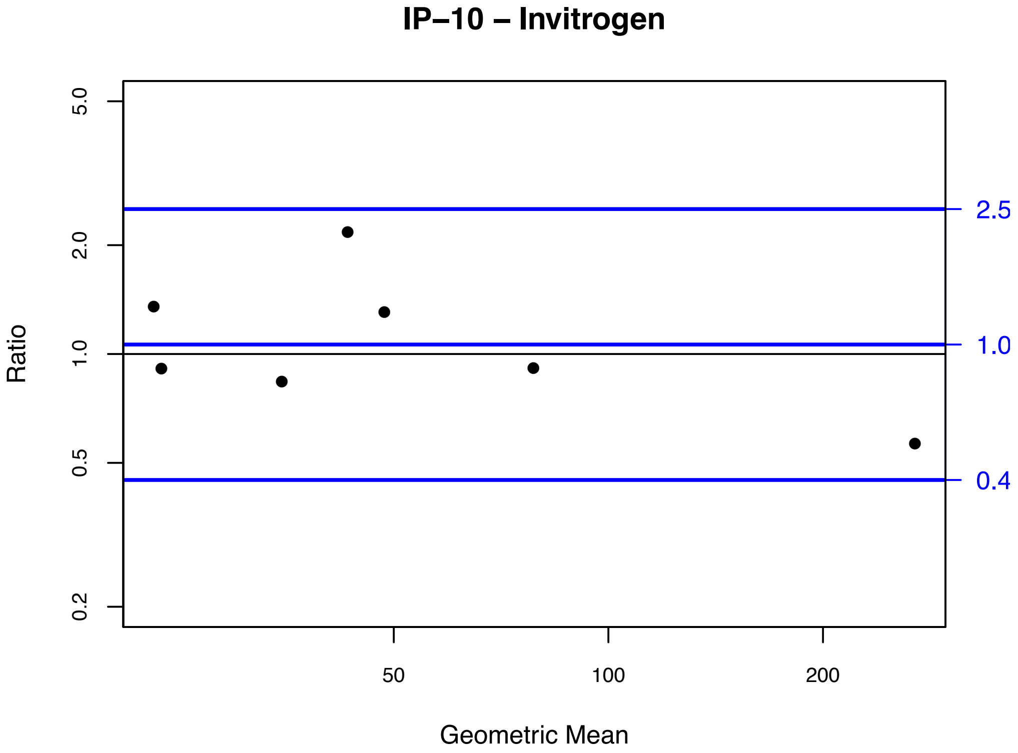

B

	parameter	value
1	Cytokine	IP-10
2	Vendor	INV_MAG
3	Samples included in this agreement	40
4	Proportion of both readings in range	100.0
5	Limits of agreement	0.79 to 1.36
6	Constant variance p.value	0.243
7	Constant ratio p.value	0.377
8	Ratio is 1 p.value	0.131

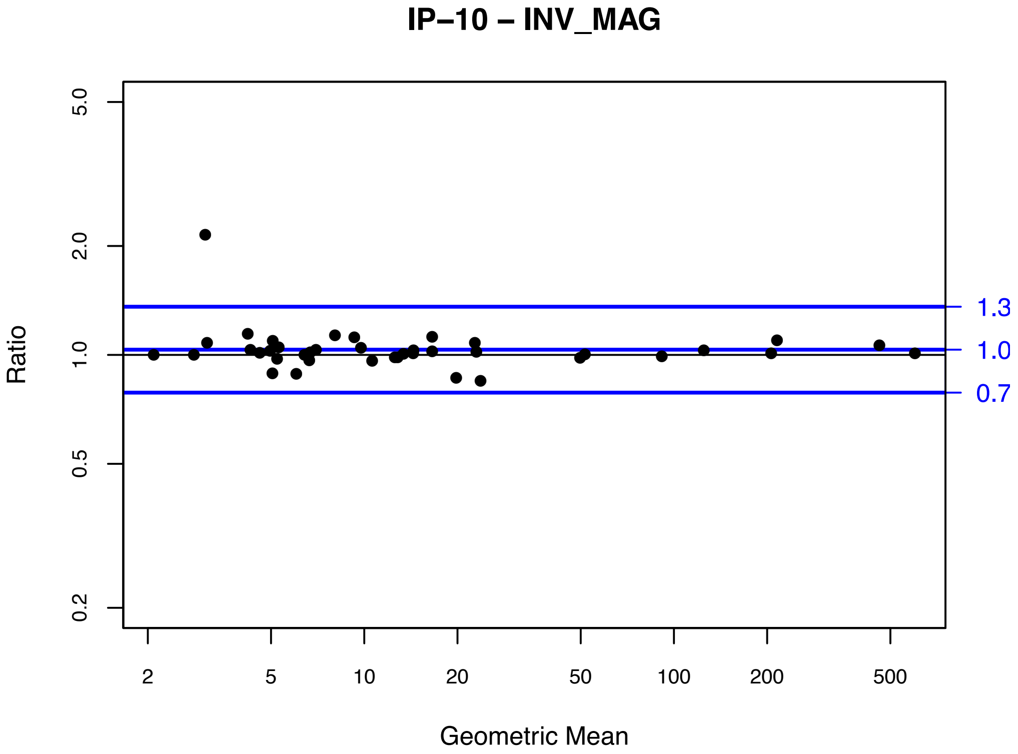

Supplement: Figure S22 — Mean difference dot plots of IP-10 for each kit tested. Disagreement plots show the difference between the duplicates against the geometric mean of both values of a sample tested with A) Human Cytokine 25-Plex panel from Invitrogen™ (non-magnetic beads) and B) Invitrogen™ Human Cytokine Magnetic 30-Plex Panel (INV-MAG). The middle line is the mean difference and the two extreme lines are the limits of agreement calculated by Bland-Altman test. (PDF) [file pone.0052587.s022.pdf]
