# Supplementary material for: Performance of Multiplex Commercial Kits to Quantify Cytokine and Chemokine Responses in Culture Supernatants from Plasmodium falciparum Stimulations
Source: PLoS One. 2013 Jan 2;8(1):e52587. doi: 10.1371/journal.pone.0052587 (PMC3534665; doi:10.1371/journal.pone.0052587)

Figure S23

A

	parameter	value
1	Cytokine	MCP-1
2	Vendor	Bio-Rad
3	Samples included in this agreement	15
4	Proportion of both readings in range	40.5
5	Limits of agreement	0.73 to 1.45
6	Constant variance p.value	0.488
7	Constant ratio p.value	0.103
8	Ratio is 1 p.value	0.483

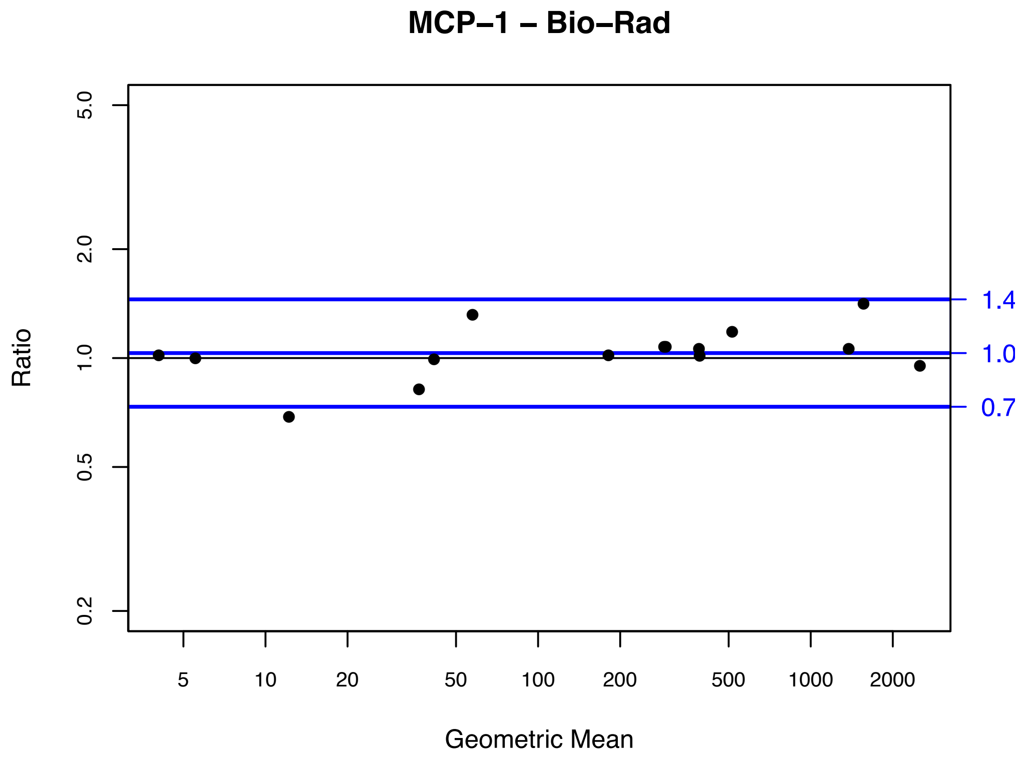

B

	parameter	value
1	Cytokine	MCP-1
2	Vendor	Invitrogen
3	Samples included in this agreement	21
4	Proportion of both readings in range	56.8
5	Limits of agreement	0.39 to 2.30
6	Constant variance p.value	0.829
7	Constant ratio p.value	0.532
8	Ratio is 1 p.value	0.608

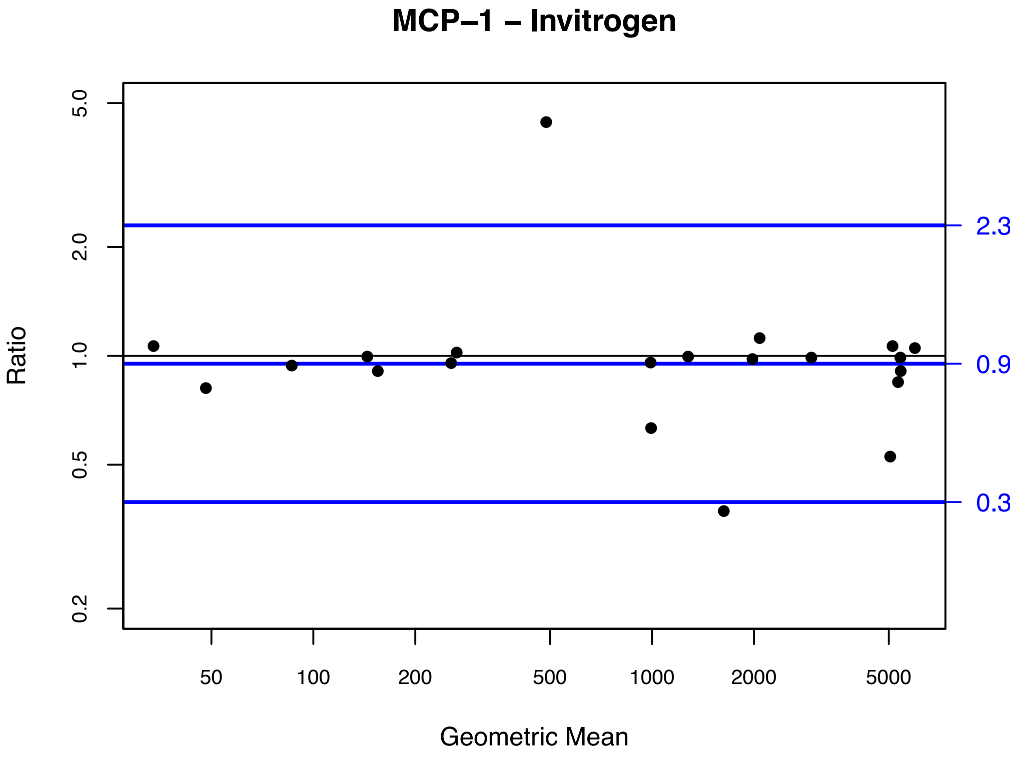

C

	parameter	value
1	Cytokine	MCP-1
2	Vendor	INV_MAG
3	Samples included in this agreement	24
4	Proportion of both readings in range	60.0
5	Limits of agreement	0.81 to 1.28
6	Constant variance p.value	0.251
7	Constant ratio p.value	0.371
8	Ratio is 1 p.value	0.384

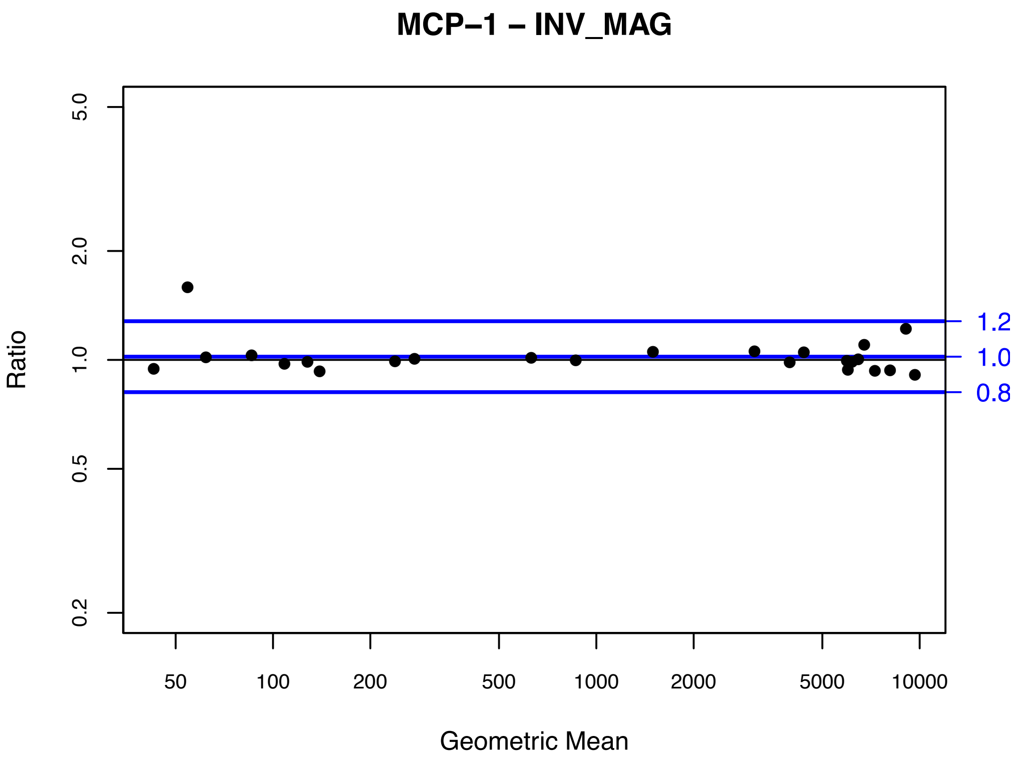

Supplement: Figure S23 — Mean difference dot plots of MCP-1 for each kit tested. Disagreement plots show the difference between the duplicates against the geometric mean of both values of a sample tested with A) Bio-Rad® Bio-Plex Pro™ Human Cytokine Plex Assay (Bio-Rad) B) Human Cytokine 25-Plex panel from Invitrogen™ (non-magnetic beads) and C) Invitrogen™ Human Cytokine Magnetic 30-Plex Panel (INV-MAG). The middle line is the mean difference and the two extreme lines are the limits of agreement calculated by Bland-Altman test. (PDF) [file pone.0052587.s023.pdf]
