# Supplementary material for: Performance of Multiplex Commercial Kits to Quantify Cytokine and Chemokine Responses in Culture Supernatants from Plasmodium falciparum Stimulations
Source: PLoS One. 2013 Jan 2;8(1):e52587. doi: 10.1371/journal.pone.0052587 (PMC3534665; doi:10.1371/journal.pone.0052587)

Figure S25

A

	parameter	value
1	Cytokine	MIP-1alpha
2	Vendor	Invitrogen
3	Samples included in this agreement	21
4	Proportion of both readings in range	56.8
5	Limits of agreement	0.46 to 2.65
6	Constant variance p.value	0.209
7	Constant ratio p.value	0.727
8	Ratio is 1 p.value	0.338

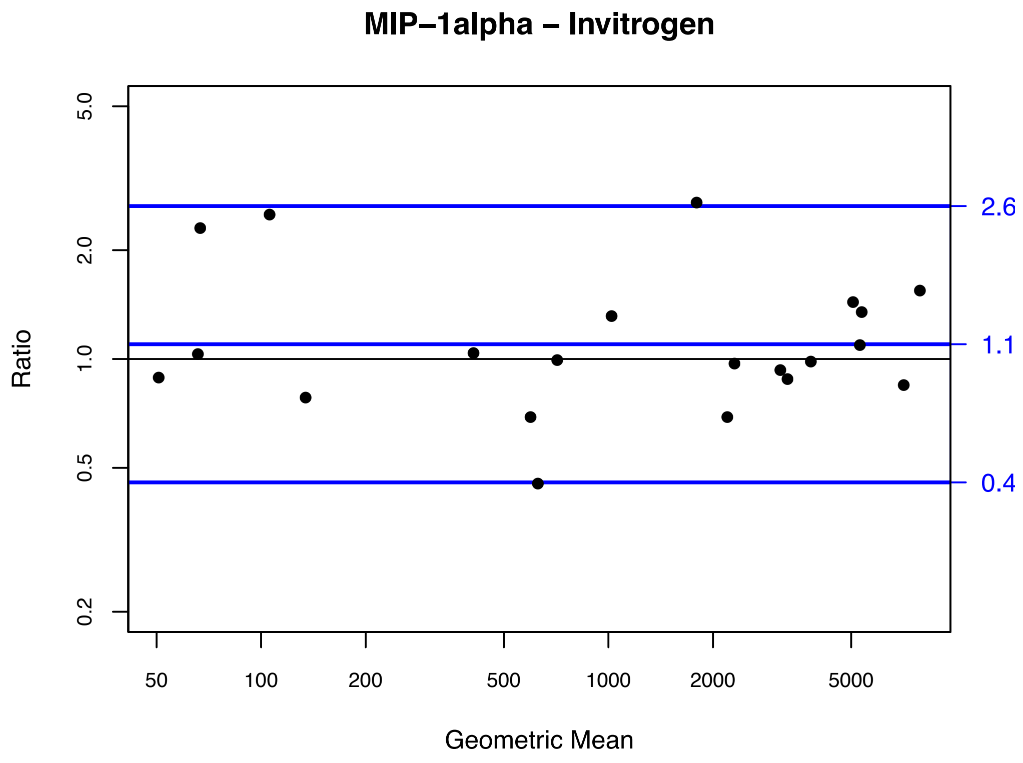

B

	parameter	value
1	Cytokine	MIP-1alpha
2	Vendor	INV_MAG
3	Samples included in this agreement	39
4	Proportion of both readings in range	97.5
5	Limits of agreement	0.80 to 1.22
6	Constant variance p.value	0.026
7	Constant ratio p.value	0.129
8	Ratio is 1 p.value	0.471

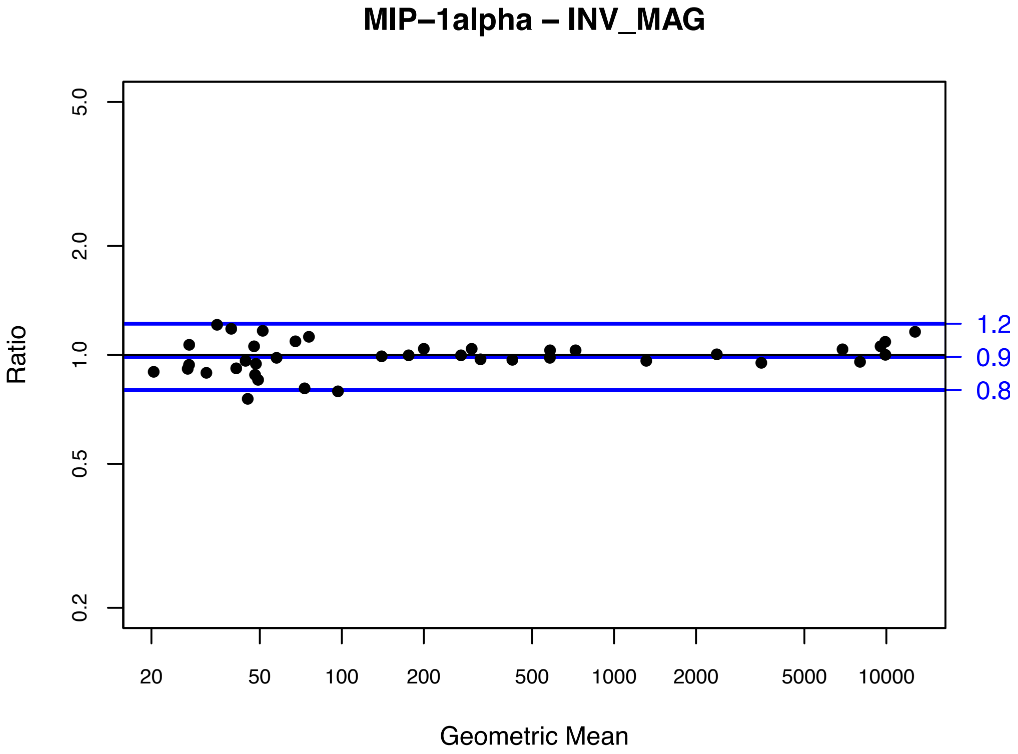

Supplement: Figure S25 — Mean difference dot plots of MIP-1α for each kit tested. Disagreement plots show the difference between the duplicates against the geometric mean of both values of a sample tested with A) Human Cytokine 25-Plex panel from Invitrogen™ (non-magnetic beads) and B) Invitrogen™ Human Cytokine Magnetic 30-Plex Panel (INV-MAG). The middle line is the mean difference and the two extreme lines are the limits of agreement calculated by Bland-Altman test. (PDF) [file pone.0052587.s025.pdf]
