# Supplementary material for: Performance of Multiplex Commercial Kits to Quantify Cytokine and Chemokine Responses in Culture Supernatants from Plasmodium falciparum Stimulations
Source: PLoS One. 2013 Jan 2;8(1):e52587. doi: 10.1371/journal.pone.0052587 (PMC3534665; doi:10.1371/journal.pone.0052587)

Figure S26

A

	parameter	value
1	Cytokine	MIP-1beta
2	Vendor	Bio-Rad
3	Samples included in this agreement	20
4	Proportion of both readings in range	54.1
5	Limits of agreement	0.72 to 1.37
6	Constant variance p.value	0.523
7	Constant ratio p.value	0.002
8	Ratio is 1 p.value	0.798

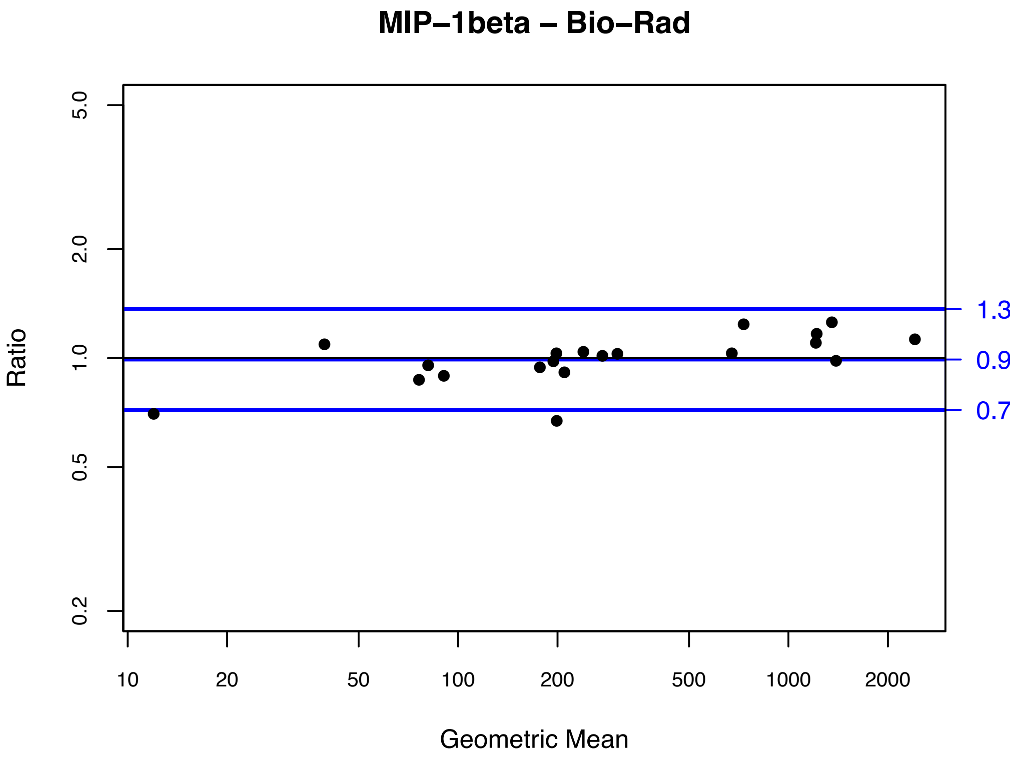

B

	parameter	value
1	Cytokine	MIP-1beta
2	Vendor	Invitrogen
3	Samples included in this agreement	21
4	Proportion of both readings in range	56.8
5	Limits of agreement	0.47 to 2.30
6	Constant variance p.value	0.228
7	Constant ratio p.value	0.956
8	Ratio is 1 p.value	0.680

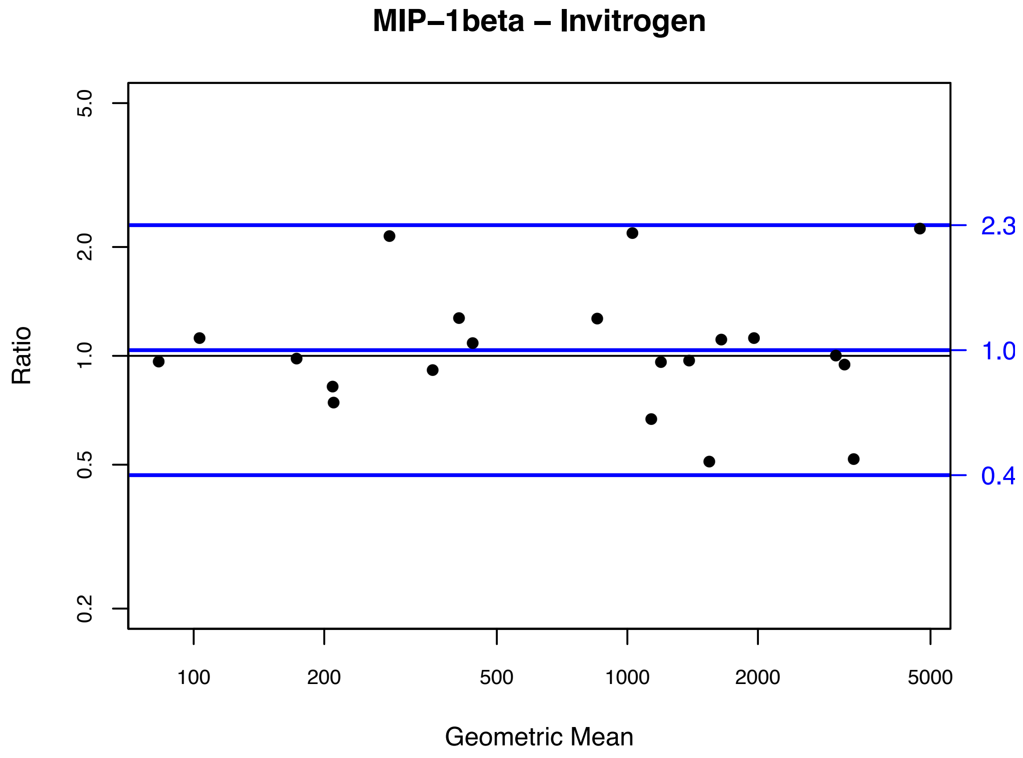

C

	parameter	value
1	Cytokine	MIP-1beta
2	Vendor	INV_MAG
3	Samples included in this agreement	27
4	Proportion of both readings in range	67.5
5	Limits of agreement	0.69 to 1.39
6	Constant variance p.value	0.000
7	Constant ratio p.value	0.234
8	Ratio is 1 p.value	0.576

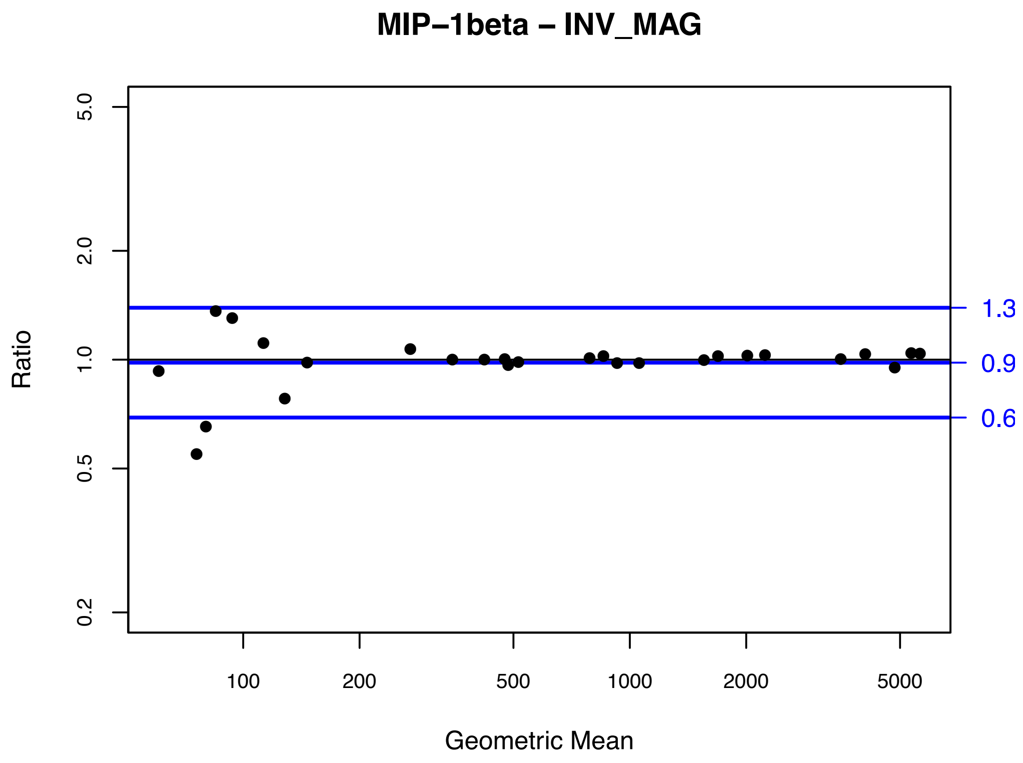

Supplement: Figure S26 — Mean difference dot plots of MIP-1β for each kit tested. Disagreement plots show the difference between the duplicates against the geometric mean of both values of a sample tested with A) Bio-Rad® Bio-Plex Pro™ Human Cytokine Plex Assay (Bio-Rad). B) Human Cytokine 25-Plex panel from Invitrogen™ (non-magnetic beads) and C) Invitrogen™ Human Cytokine Magnetic 30-Plex Panel (INV-MAG). The middle line is the mean difference and the two extreme lines are the limits of agreement calculated by Bland-Altman test. (PDF) [file pone.0052587.s026.pdf]
