# Supplementary material for: Performance of Multiplex Commercial Kits to Quantify Cytokine and Chemokine Responses in Culture Supernatants from Plasmodium falciparum Stimulations
Source: PLoS One. 2013 Jan 2;8(1):e52587. doi: 10.1371/journal.pone.0052587 (PMC3534665; doi:10.1371/journal.pone.0052587)

## Figure S27

### A

| | parameter | value |
|---|---|---|
| 1 | Cytokine | RANTES |
| 2 | Vendor | Invitrogen |
| 3 | Samples included in this agreement | 33 |
| 4 | Proportion of both readings in range | 89.2 |
| 5 | Limits of agreement | 0.49 to 2.17 |
| 6 | Constant variance p.value | 0.748 |
| 7 | Constant ratio p.value | 0.406 |
| 8 | Ratio is 1 p.value | 0.627 |

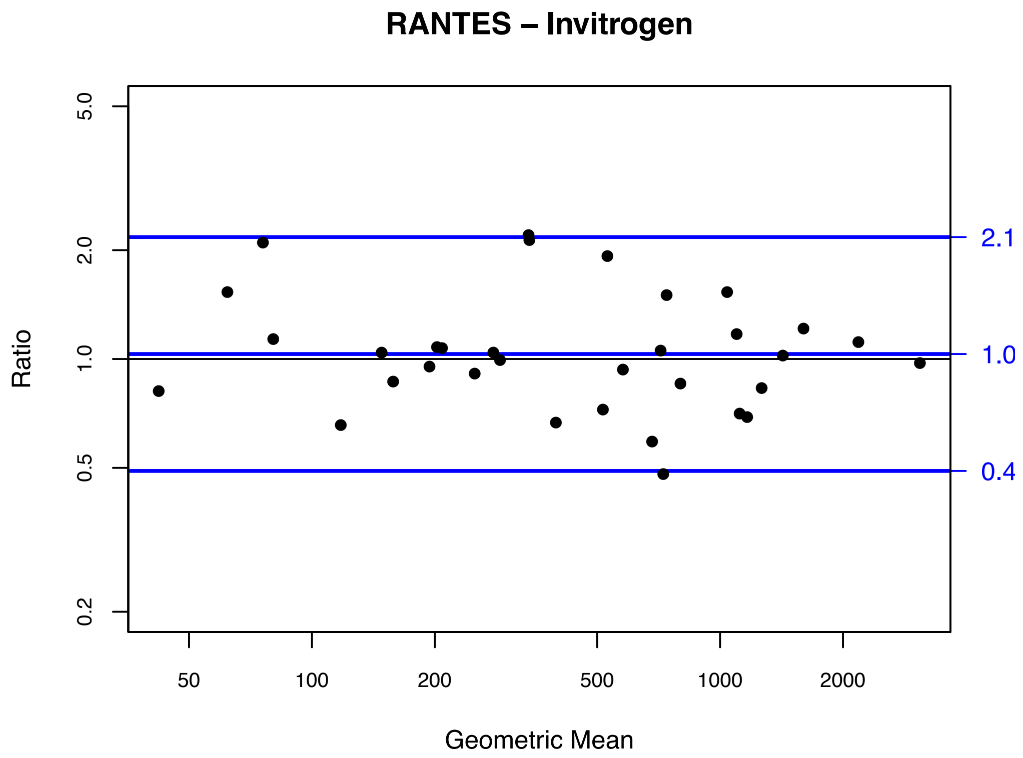

**B**

| | parameter | value |
|---|---|---|
| 1 | Cytokine | RANTES |
| 2 | Vendor | INV_MAG |
| 3 | Samples included in this agreement | 40 |
| 4 | Proportion of both readings in range | 100.0 |
| 5 | Limits of agreement | 0.73 to 1.33 |
| 6 | Constant variance p.value | 0.315 |
| 7 | Constant ratio p.value | 0.249 |
| 8 | Ratio is 1 p.value | 0.552 |

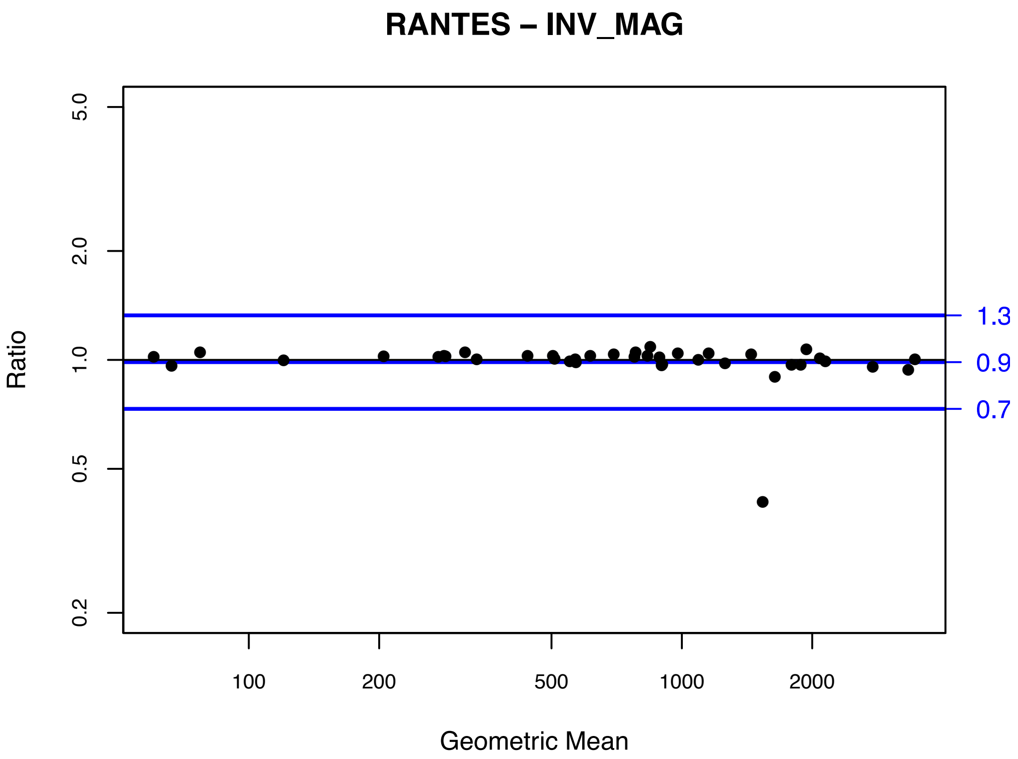

Supplement: Figure S27 — Mean difference dot plots of RANTES for each kit tested. Disagreement plots show the difference between the duplicates against the geometric mean of both values of a sample tested with A) Human Cytokine 25-Plex panel from Invitrogen™ (non-magnetic beads) and B) Invitrogen™ Human Cytokine Magnetic 30-Plex Panel (INV-MAG). The middle line is the mean difference and the two extreme lines are the limits of agreement calculated by Bland-Altman test. (PDF) [file pone.0052587.s027.pdf]
