# Supplementary material for: Performance of Multiplex Commercial Kits to Quantify Cytokine and Chemokine Responses in Culture Supernatants from Plasmodium falciparum Stimulations
Source: PLoS One. 2013 Jan 2;8(1):e52587. doi: 10.1371/journal.pone.0052587 (PMC3534665; doi:10.1371/journal.pone.0052587)

# Figure S28

## A

| | parameter | value |
|---|---|---|
| 1 | Cytokine | TNF-alpha |
| 2 | Vendor | Bender |
| 3 | Samples included in this agreement | 16 |
| 4 | Proportion of both readings in range | 43.2 |
| 5 | Limits of agreement | 0.62 to 1.97 |
| 6 | Constant variance p.value | 0.807 |
| 7 | Constant ratio p.value | 0.026 |
| 8 | Ratio is 1 p.value | 0.188 |

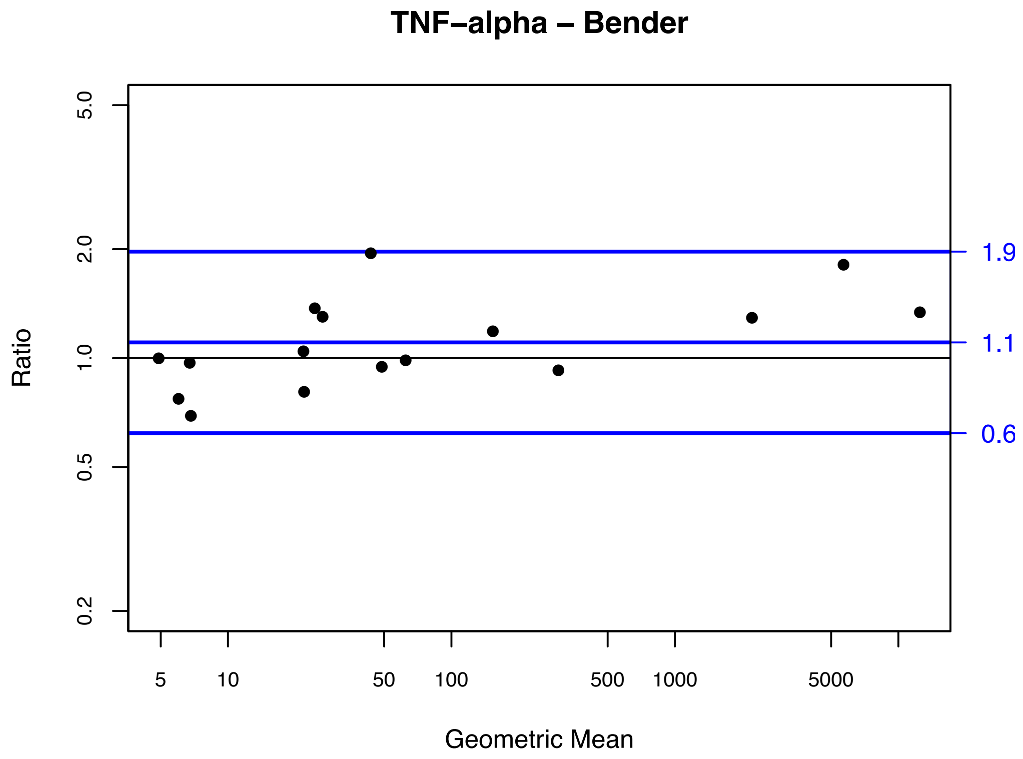

**B**

| | parameter | value |
|---|---|---|
| 1 | Cytokine | TNF-alpha |
| 2 | Vendor | Bio-Rad |
| 3 | Samples included in this agreement | 20 |
| 4 | Proportion of both readings in range | 54.1 |
| 5 | Limits of agreement | 0.77 to 1.56 |
| 6 | Constant variance p.value | 0.797 |
| 7 | Constant ratio p.value | 0.974 |
| 8 | Ratio is 1 p.value | 0.037 |

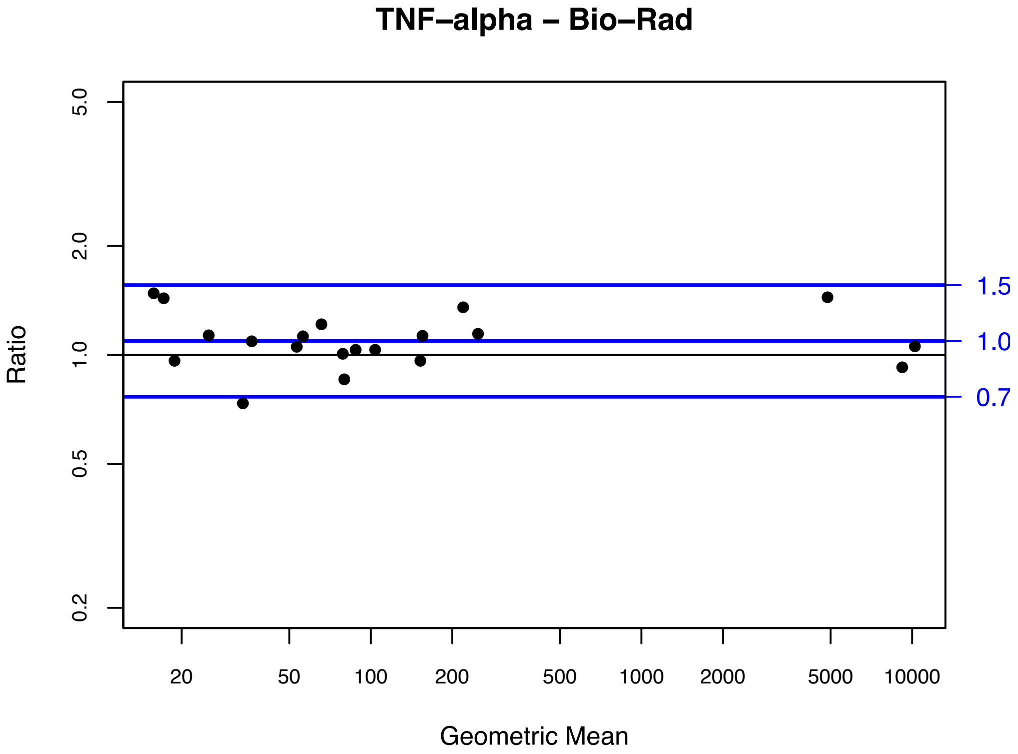

**C**

| | parameter | value |
|---|---|---|
| 1 | Cytokine | TNF-alpha |
| 2 | Vendor | Invitrogen |
| 3 | Samples included in this agreement | 5 |
| 4 | Proportion of both readings in range | 13.5 |
| 5 | Limits of agreement | 0.31 to 7.56 |
| 6 | Constant variance p.value | 0.154 |
| 7 | Constant ratio p.value | 0.065 |
| 8 | Ratio is 1 p.value | 0.290 |

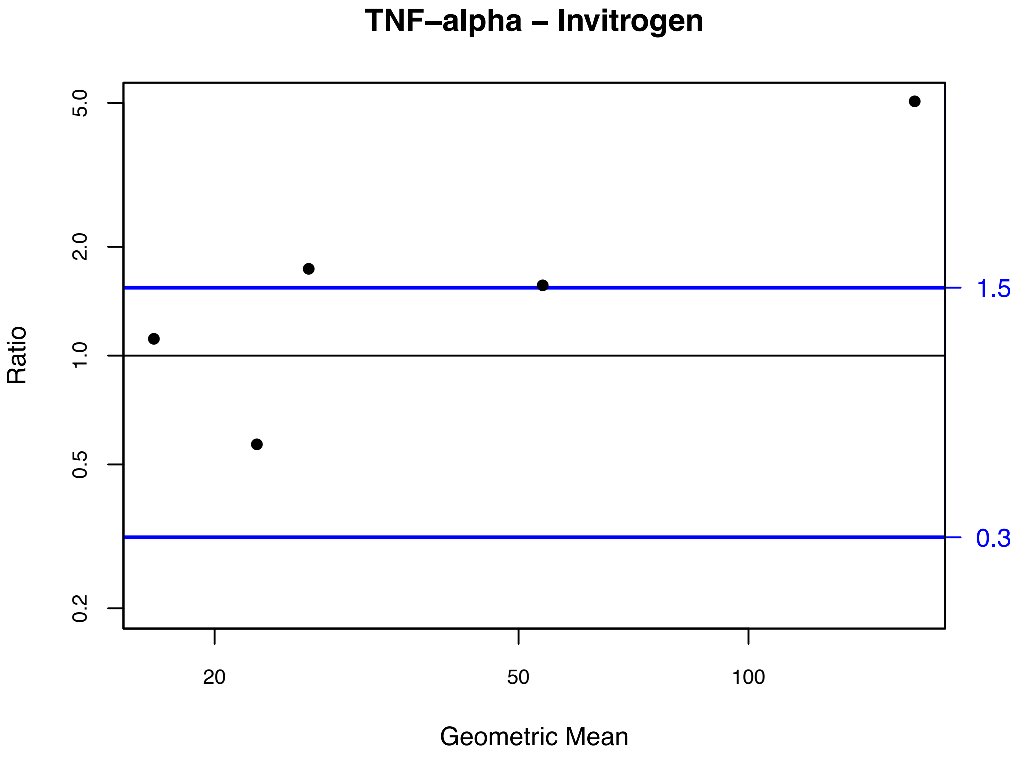

**D**

| | parameter | value |
|---|---|---|
| 1 | Cytokine | TNF-alpha |
| 2 | Vendor | INV_MAG |
| 3 | Samples included in this agreement | 24 |
| 4 | Proportion of both readings in range | 60.0 |
| 5 | Limits of agreement | 0.58 to 1.45 |
| 6 | Constant variance p.value | 0.064 |
| 7 | Constant ratio p.value | 0.518 |
| 8 | Ratio is 1 p.value | 0.090 |

**TNF–alpha – INV_MAG**

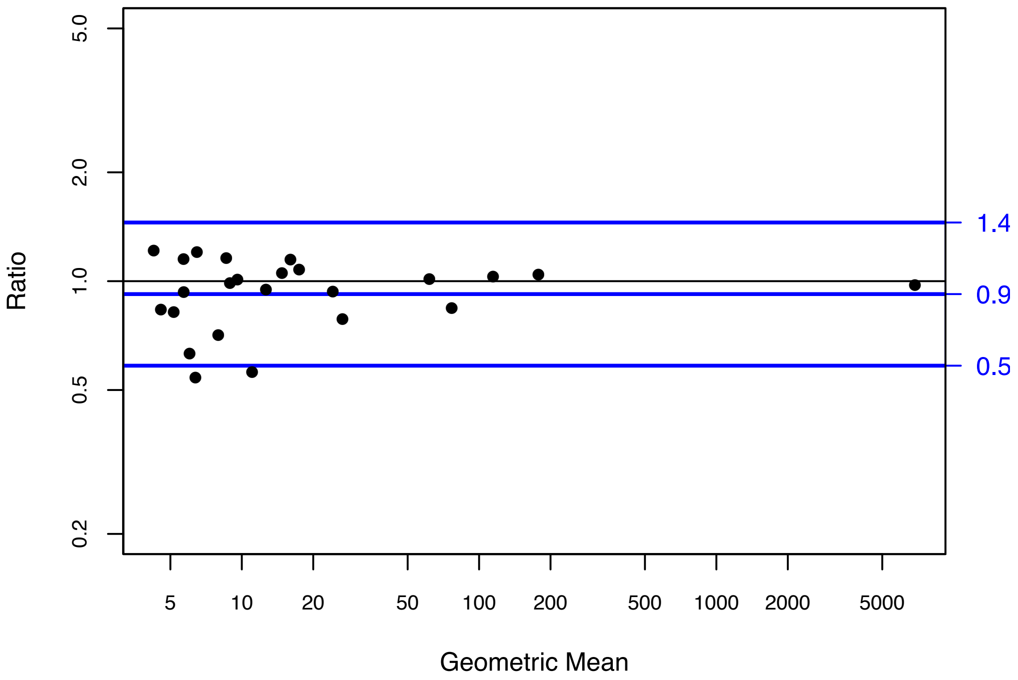

**E**

| | parameter | value |
|---|---|---|
| 1 | Cytokine | TNF-alpha |
| 2 | Vendor | Millipore |
| 3 | Samples included in this agreement | 28 |
| 4 | Proportion of both readings in range | 75.7 |
| 5 | Limits of agreement | 0.82 to 1.22 |
| 6 | Constant variance p.value | 0.595 |
| 7 | Constant ratio p.value | 0.388 |
| 8 | Ratio is 1 p.value | 0.972 |

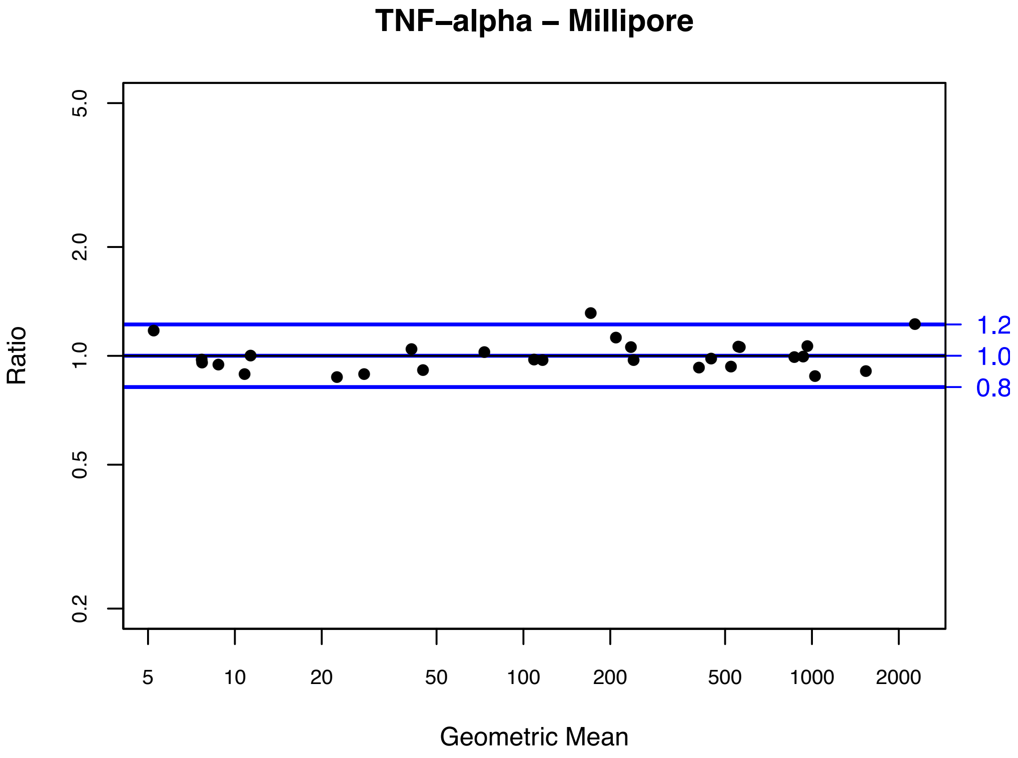

Supplement: Figure S28 — Mean difference dot plots of TNF-α for each kit tested. Disagreement plots show the difference between the duplicates against the geometric mean of both values of a sample tested with A) eBioscience® FlowCytomix™ (Bender), B) Bio-Rad® Bio-Plex Pro™ Human Cytokine Plex Assay (Bio-Rad), C) Human Cytokine 25-Plex panel from Invitrogen™ (non-magnetic beads), D) Invitrogen™ Human Cytokine Magnetic 30-Plex Panel (INV-MAG), and E) Millipore™ MILLIPLEX® MAP Plex Kit (Millipore). The middle line is the mean difference and the two extreme lines are the limits of agreement calculated by Bland-Altman test. (PDF) [file pone.0052587.s028.pdf]
