# Supplementary material for: Performance of Multiplex Commercial Kits to Quantify Cytokine and Chemokine Responses in Culture Supernatants from Plasmodium falciparum Stimulations
Source: PLoS One. 2013 Jan 2;8(1):e52587. doi: 10.1371/journal.pone.0052587 (PMC3534665; doi:10.1371/journal.pone.0052587)

Figure S29

	parameter	value
1	Cytokine	TNF-beta
2	Vendor	Millipore
3	Samples included in this agreement	4
4	Proportion of both readings in range	10.8
5	Limits of agreement	0.81 to 1.09
6	Constant variance p.value	0.505
7	Constant ratio p.value	0.879
8	Ratio is 1 p.value	0.209

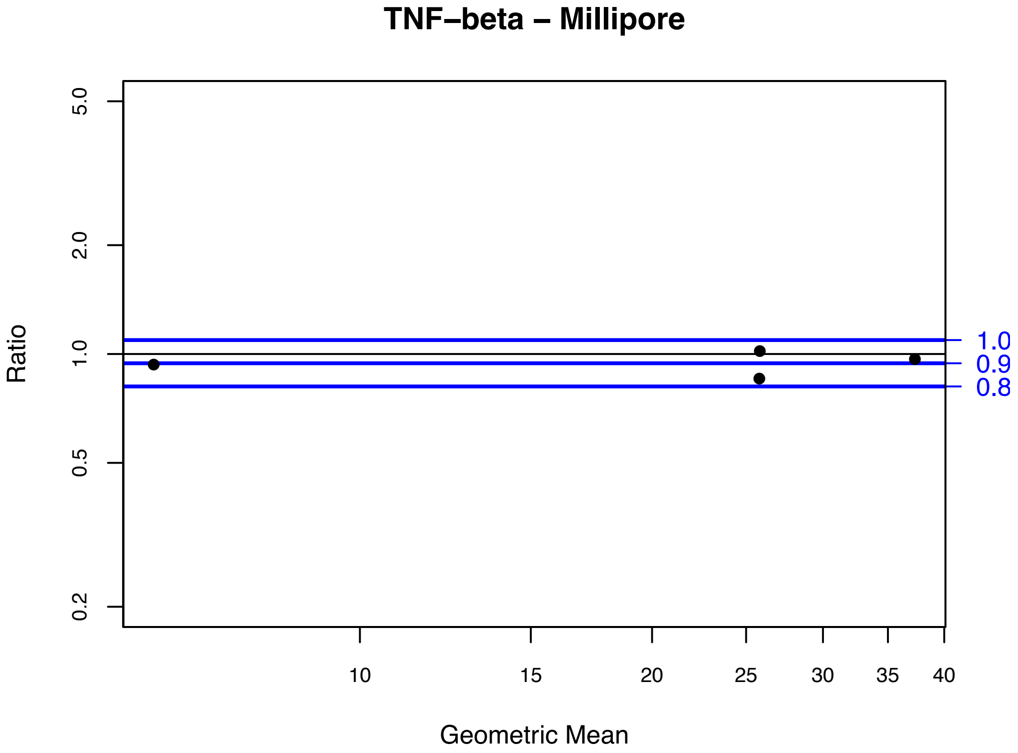

Supplement: Figure S29 — Mean difference dot plot of TNF-β. Disagreement plots show the difference between the duplicates against the geometric mean of both values of a sample tested with Millipore™ MILLIPLEX® MAP Plex Kit (Millipore). The middle line is the mean difference and the two extreme lines are the limits of agreement calculated by Bland-Altman test. (PDF) [file pone.0052587.s029.pdf]
