# Supplementary material for: Performance of Multiplex Commercial Kits to Quantify Cytokine and Chemokine Responses in Culture Supernatants from Plasmodium falciparum Stimulations
Source: PLoS One. 2013 Jan 2;8(1):e52587. doi: 10.1371/journal.pone.0052587 (PMC3534665; doi:10.1371/journal.pone.0052587)

Figure S30

	parameter	value
1	Cytokine	VEGF
2	Vendor	INV_MAG
3	Samples included in this agreement	39
4	Proportion of both readings in range	97.5
5	Limits of agreement	0.70 to 1.47
6	Constant variance p.value	0.084
7	Constant ratio p.value	0.025
8	Ratio is 1 p.value	0.669

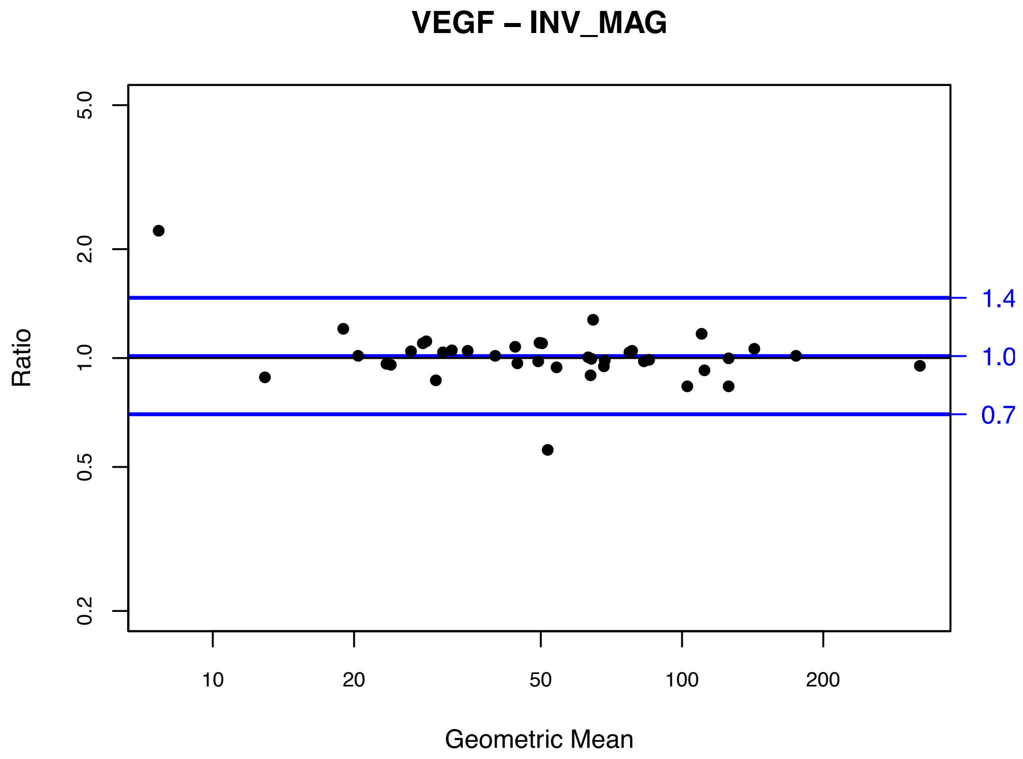

Supplement: Figure S30 — Mean difference dot plot of VEGF. Disagreement plots show the difference between the duplicates against the geometric mean of both values of a sample tested with Invitrogen™ Human Cytokine Magnetic 30-Plex Panel (INV-MAG). The middle line is the mean difference and the two extreme lines are the limits of agreement calculated by Bland-Altman test. (PDF) [file pone.0052587.s030.pdf]
